# A Novel Class of 7‐Membered Heterocyclic Compounds

**DOI:** 10.1002/ejoc.202000363

**Published:** 2020-05-06

**Authors:** Adriano Bauer, Eszter Borsos, Nuno Maulide

**Affiliations:** ^1^ Institute of Organic Chemistry University of Vienna Währinger Straße 38 1090 Vienna Austria

**Keywords:** Amide activation, Cyclization, Heterocycles, 7‐Membered rings, Synthetic methods

## Abstract

The work presented herein describes the synthesis of a formerly inaccessible class of heterocyclic compounds. The reaction relies on α‐phthalimido‐amides, which are readily prepared from amino acids in 2 simple reactions steps. Under amide activation conditions in which classical keteniminium ions are not formed, the nitrile solvent is incorporated into the new fused 7‐membered ring system. Due to the absence of a keteniminium intermediate, the stereogenic information in the α‐position is fully retained.

## Graphical abstract

We report that α‐phthalimido‐amides can be converted into unusual 7‐membered heterocycles by electrophilic amide activation. In this transformation, where the nitrile solvent is incorporated into the final product, a keteniminium intermediate appears not to be involved.

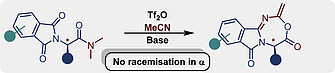

## Introduction

**Scheme 1:**
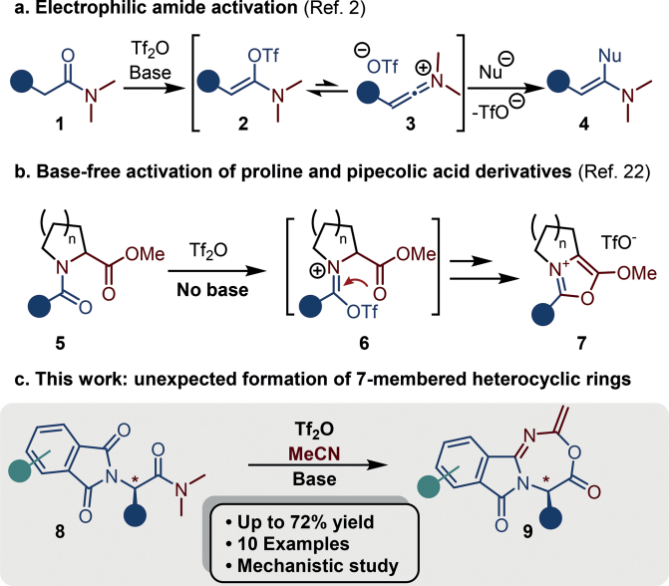
a) Modern approach to classical amide activation. b) Synthesis of alkyl‐substituted oxazolium salts by amide activation in absence of a base. c) Formation of a novel class of 7‐membered rings *via* amide activation in absence of keteniminium ions.

The enhanced nucleophilicity of amides in comparison to other carbonyl functionalities has been noted already in the 19^th^ century.^1^ This peculiar reactivity was later leveraged into so‐called electrophilic amide activation, a reactivity principle that relies on the treatment of the amide with a strong electrophile such as trifluoromethanesulfonic anhydride (triflic anhydride) and a base in modern approaches (Scheme 1 *a*).^2^ In doing so, the amide is transformed into an α‐trifloxyenamine **2**, which resides in a biased equilibrium with the keteniminium salt **3**. The latter, a highly electrophilic species, can be intercepted by several nucleophiles, including nucleophilic oxidants,^3^, ^4^, ^5^, ^6^, ^7^ and enables a range of unconventional reactivity profiles for the atoms surrounding the original amide functionality.^8^, ^9^, ^10^, ^11^, ^12^ The capture of the central electrophilic atom of the keteniminium by modestly nucleophilic entities such as alkenes (in formal [2+2] cycloaddition reactions^13^, ^14^ or other ring‐forming events)^15^ or ethers^16^, ^17^ is a well‐documented phenomenon. Although the chemistry of amide activation is dominated by keteniminium formation from tertiary amides, the use of secondary amide starting materials or the outright avoidance of keteniminium formation can be similarly rewarding.^18^, ^19^, ^20^, ^21^, ^22^ It is noteworthy that amide activation is generally a versatile tool for the construction of heterocyclic compounds.^19^, ^21^, ^23^, ^24^, ^25^, ^26^, ^27^, ^28^

We recently reported that capture of an activated amide, at the iminium triflate stage, by a proximal ester affords previously inaccessible oxazolium salts (Scheme 1 *b*).^25^ Omission of the base was crucial because it precluded keteniminium formation (and consequently prevented unproductive reaction pathways). Herein we would like to report a synthesis of structurally novel 7‐membered heterocyclic rings, which similarly relies on the activation of tertiary amides in the absence of keteniminium species (Scheme 1 *c*). This class of compounds has, to the best of our knowledge, not been accessible so far and represents an entirely novel scaffold.

## Results and Discussion

**Scheme 2:**
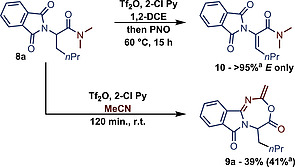
Serendipitous discovery of 7‐membered heterocycle **9a**. Yields refer to pure, isolated material unless otherwise stated. ^a^ NMR Yield. See SI for details.

In the course of our studies on α,β‐dehydrogenation of α‐branched amides we investigated the amino‐acid derivative **8a** under the typical conditions of amide activation (Scheme 2).^4^, ^29^ The expected desaturation (to product **10**) was observed when the activated amide was treated with pyridine *N*‐oxide in 1,2‐DCE upon heating. However, we noticed that when acetonitrile was used as the solvent, the main product of the transformation was the 7‐membered ring **9a**. Inspection of the structure reveals that the nitrile solvent was incorporated into the final product, and indeed nitriles are known to attack activated amides.^24^ However, in this case, the nitrile‐derived fragment is not attached to the former amide functionality. We further noticed that product **9a** was not particularly stable and decomposed slowly under air at room temperature upon standing.

During optimization, which was undertaken on substrate **8b**, we first noticed that the yield was considerably lower on this very similar substrate under the previously described conditions (Table 1 – Entry 1). Interestingly, the reaction proceeded in the absence of a base (Entry 2/3). Moreover, no desired product was observed when pyridine was used as the base (Entry 4). These results indicated that, similar to our previous study, (Scheme 1 *b*) the avoided formation of a keteniminium intermediate might be crucial to allowing the formation of **9b**. Indeed, when the sterically hindered proton scavenger 2,6‐*ditert*‐butylpyridine (DTBP) was applied, a base that is most likely unable to deprotonate the α‐methine of the activated amide under these conditions, the yield increased considerably (Entry 5). This is consistent with the fact that DTBP was only observed to promote keteniminium formation when less sterically hindered α‐methylene protons were abstracted,^30^ although the temperature might also play a decisive role.^31^ Concluding on the experiments of Table 1, the reaction might not need a base that directly participates in the formation of the product. It is more likely that DTPB acts as a proton scavenger to improve the yield. Proton scavengers have been indeed observed to improve the general efficiency of amide activation reactions.^32^

**Table 1:** Optimization of the 7‐membered ring formation

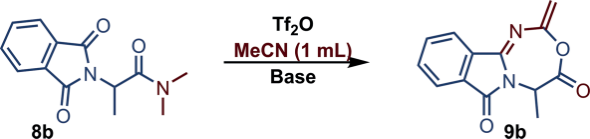						
Entry	Tf_2_O equiv.	Base	Base eq.	Time	Yield^a^	Yield
1	1.1	2‐Cl Py	2.2	0.5 h	15 %	/
2	3.0	/	/	0.5 h	41 %	/
3	1.1	/	/	2 h	15 %	/
4	1.1	Py	2.2	0.5 h	0 %	/
5	1.1	DTBP	2.2	0.5 h	55 %	48 %

a Yield determined by ^1^H NMR analysis of the crude product with mesitylene as the internal standard.

**Scheme 3:**
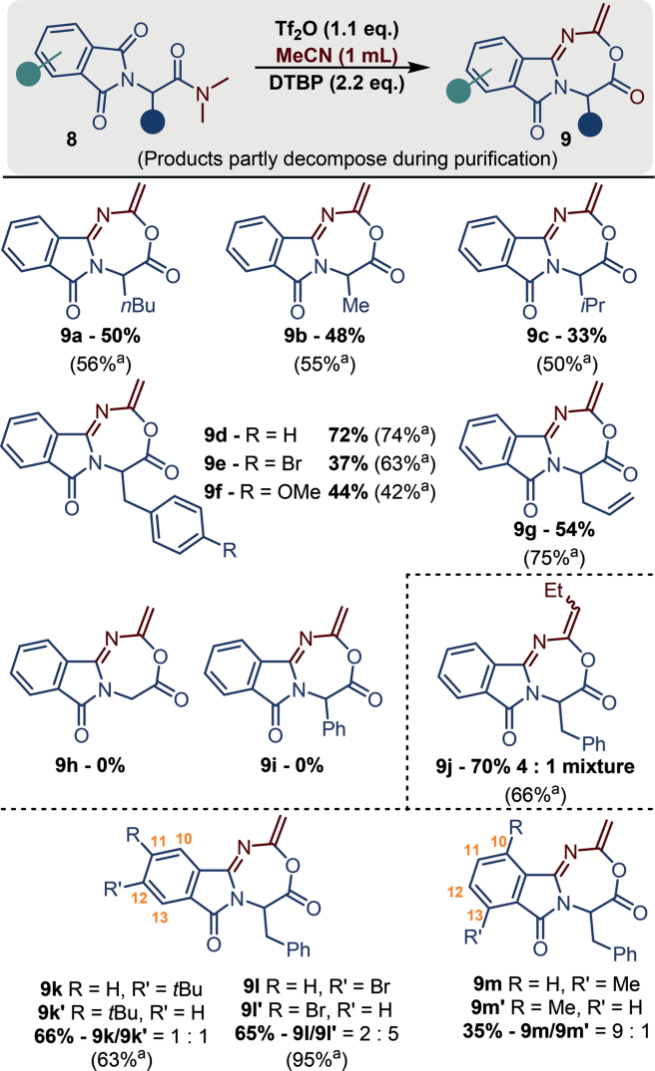
Product scope of the 7‐membered ring formation. The yields refer to pure, isolated material unless otherwise stated. ^a^ Yields determined by ^1^H NMR analysis of the crude reaction mixture with mesitylene as the internal standard.

With suitable conditions in hand, we investigated if other substrates undergo this unusual reactivity (Scheme 3). First, a range of substituents in the α‐position of the amide was investigated. As shown, variations on the structure of **8a/8b** were tolerated. It seems that a number of α‐benzyl or ‐allyl‐substituted amides were generally privileged substrates for this transformation (*cf*. **9d**–**g**). In many cases, the yields determined by ^1^H NMR analysis of the crude mixture with mesitylene as the internal standard resulted much higher than the isolated. This is most likely due to the decomposition of the unusual heterocycle during purification *via* column chromatography. Neither the unsubstituted **9h** nor its α‐phenyl‐substituted cognate **9i** could be generated by this approach, leading instead to complex mixtures. α‐Succinimide amides gave exclusively the starting material back.

Next, we decided to screen other nitrile solvents and elected to assay butyronitrile as the reaction partner for this transformation (*cf*. **9j**). The resulting product **9j** was formed in a good 70 % yield.

Last, we studied the influence of substituents on the phthalimide moiety. While an electron‐donating group on the distal position (carbon *11/12*) gave a 1:1 mixture of two regioisomers, an electron‐withdrawing bromide showed a moderate selectivity for the isomer **9l**'. A methyl group in the proximal position (carbon *10/13*) showed a rather distinct regioselectivity of 9:1. 2D‐NMR analysis of the mixture suggests that the major isomer is surprisingly **9m'**, *i.e*. with the methyl group in direct proximity to the amidine moiety.

The stability of these intriguing structures is, not surprisingly, limited. The remarkable concatenation of an enolester, a 2‐azadiene, and a phthalimide‐derived amidine is, to the best of our knowledge, unprecedented. Most of the products tended to decompose on silica during column chromatography, systematically resulting in diminished isolated yields when compared to crude NMR assays with an internal standard. When the silica was basified with 1 % of trimethylamine or neutral Al_2_O_3_ was used as the stationary phase, products decomposed completely. The use of Florisil® on the other hand, resulted in similar yields (compared to silica gel).

**Scheme 4:**
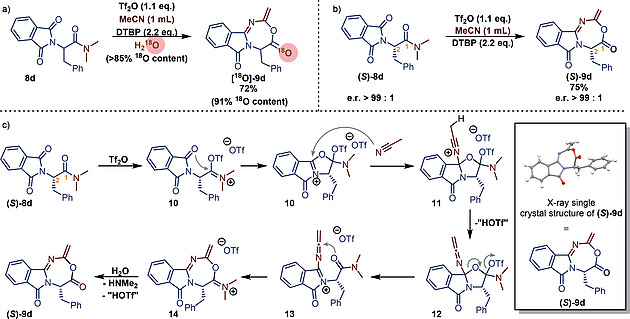
a) 18O‐labeling study. b) Evidence for the absence of keteniminium/vinyltrilfate formation c) Proposed mechanism.

Thereafter mechanistic investigations were undertaken. The reaction mixture becomes strongly colored after triflic anhydride is added and usually turns black after the reaction time of 30 minutes. However, during the aqueous quench, the black turbid color fades quickly to a clear red/orange/yellow solution (depending on the substrate). This observation led us to the assumption that an iminium ion might be transiently formed, which is quickly hydrolyzed to the observed lactone moiety. To support this hypothesis, we quenched the reaction with ^18^O‐labelled water and could indeed observe the full incorporation of the isotope by mass spectrometry (Scheme 4 *a*).

In an additional experiment, the enantioenriched (*e.r*. > 99 %) phenylalanine derivative (**8d**) was subjected to the reaction conditions. Interestingly, no racemization of the α‐position took place under the optimal reaction conditions (carbon 2 in Scheme 4 *b*), strongly suggesting that this reaction does not proceed *via* a keteniminium ion. Moreover, we were able to obtain a single crystal of the enantioenriched sample **(*S*)‐9d** in order to confirm the structure by X‐ray analysis.

Concluding from our experiments in Scheme 4a/b we propose the following mechanism for this transformation. Following the formation of *O*‐triflyl oxyiminium ion **10**, one of the neighboring phthalimido carbonyl oxygens attacks the highly electrophilic iminium carbon atom. The so generated oxazolinium ion **10** is then attacked by acetonitrile (the solvent) and, upon deprotonation, the ketenimine **12** is generated. This species then undergoes a 5→7 ring enlargement to yield the charged precursor of the final product (**14**), which is finally converted into the product by aqueous hydrolysis during the workup. The mechanism is somewhat reminiscent of the reversible ring‐opening of 4‐oxazolines to azomethine ylides which can act as 1,5‐dipoles,^33^ although 1,3‐dipolar cycloadditions are more commonly observed.^34^

## Conclusions

In this work, we showed that certain α‐phthalimido‐amides can be converted into unusual 7‐membered heterocyclic systems under electrophilic amide activation in acetonitrile. Noteworthy, our results strongly suggest that the reaction does not proceed *via* a keteniminium intermediate or analogous vinylic species. The formed products, with a concatenation of sensitive functional groups, can be isolated in yields up to 72 %.

## Experimental Section

**Reaction Procedure** (Scheme 3): The α‐phthalimido amide (1.0 equiv., 0.20 mmol) was dissolved in the nitrile (1 mL) and cooled to 0 °C. DTBP was added (2.2 equiv., 0.44 mmol, 99 µL), followed by trifluoromethanesulfonic anhydride (0.22 mmol, 37 µL). The reaction was then stirred at room temperature 30 minutes. Then, water (2–3 mL) and DCM (2–3 mL) were added to the mixture, resulting in a strong change in color. The aqueous phase was extracted with DCM (3 × 5 mL) and the united organic layers were dried with Na_2_SO_4_ and filtered. Volatiles were removed under reduced pressure and the crude product was purified by column chromatography using silica gel or Florisil® (eluent = heptane/EtOAc, 95:5 → 20:80 v/v%) to obtain the desired product usually as a shiny yellow solid.

## Supporting information

Supporting Information
