# Supplementary material for: A Novel Class of 7‐Membered Heterocyclic Compounds
Source: European J Org Chem. 2020 May 6;2020(26):3971–4. doi: 10.1002/ejoc.202000363 (PMC7496137; doi:10.1002/ejoc.202000363)
Supplement: Supplementary file 1 — Supporting Information [file EJOC-2020-3971-s001.pdf]

EurJOC
European Journal of Organic Chemistry

Supporting Information

A Novel Class of 7-Membered Heterocyclic Compounds

Adriano Bauer, Eszter Borsos, and Nuno Maulide*

Table of Contents

I. General information 1
II. Preparation of starting materials 2
Procedure A (Racemic starting materials) 2
Procedure B (Enantiopure starting material).. 2
III. Formation of 7-membered heterocycles 3
Procedure C 3
^{18}O labeling experiment 3
Use of enantiopure substrate (*S*)-8d 3
Further evidence for an iminium species 4
Major isomer assignment of 9m/9m' 6
IV. Characterization 7
V. Spectra..14
VI. X-ray Analysis 35

I. General information

Unless otherwise stated, all glassware was flame-dried before use and all reactions were performed under an atmosphere of argon. All solvents were distilled from appropriate drying agents prior to use. All reagents were used as received from commercial suppliers unless otherwise stated. Trifluoromethane sulfonic anhydride (triflic anhydride) was dried over P_2O_5 and distill under argon prior use. Reaction progress was monitored by thin layer chromatography (TLC) performed on aluminium plates coated with silica gel F254 with 0.2 mm thickness. Chromatograms were visualized by fluorescence quenching with UV light at 254 nm or by staining using potassium permanganate. Flash column chromatography was performed using silica gel 60 (230-400 mesh, Merck and co.). Neat infrared spectra were recorded using a Perkin-Elmer Spectrum 100 FT-IR spectrometer. Wavenumbers (vmax) are reported in cm^{-1}. Mass spectra were obtained using a Finnigan MAT 8200 or (70 eV) or an Agilent 5973 (70 eV) spectrometer, using electrospray ionization (ESI). All ^{1}H NMR and ^{13}C NMR spectra were recorded using a Bruker AV-400, AV-600 or AV-700 spectrometer at 300K. Chemical shifts were given in parts per million (ppm, δ), referenced to the solvent peak of CDCl3, defined at δ = 7.26 ppm (^{1}H-NMR) and δ = 77.16 (^{13}C NMR). Coupling constants are quoted in Hz (J). ^{1}H NMR splitting patterns were designated as singlet (s), doublet (d), triplet (t), quartet (q) as they appeared in the spectrum. Splitting patterns that could not be interpreted or easily visualized were designated as multiplet (m) or broad (br).

II. Preparation of starting materials

Procedure A (Racemic starting materials)

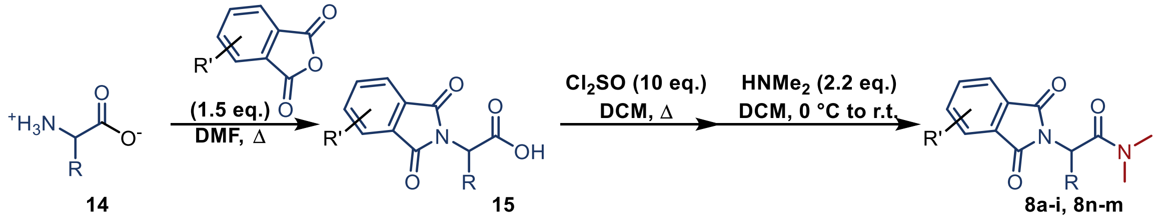

Scheme S 1

The aminoacid (1.0 eq.) was suspended in DMF (0.40 M) and the phthalic anhydride **14** (1.5 eq.) was added. The reaction was heated to reflux for 15 h. After cooling to room temperature, the crude mixture was poured into an equal volume of an aqueous HCl solution (1 M) and extracted with EtOAc (3x). The combined organic layer was dried over Na_2SO_4 and filtered. Volatiles were removed under reduced pressure and the crude product was purified by column chromatography using silica gel (typical eluent = heptane : EtOAc : AcOH – 93 : 5 : 2 → 20 : 78 : 2 v/v%) to obtain the acid.

The acid **15** was dissolved in DCM (0.13 M) and thionyl chloride (10 eq.) was added to the mixture. The reaction was heated to reflux for 4 h. Then, the volatiles were removed under reduced pressure to obtain the crude acyl chloride. The acyl chloride was dissolved in DCM (0.20 M assuming quantitative yield from the acid) and cooled to 0 °C. Then, a solution of $HNMe_2$ in THF (2 M, 2.2 eq.) was added dropwise. Thereafter, the cooling bath was removed and the reaction was stirred for 14 h. The reaction was quenched with an aqueous HCl (1 M) solution and extracted with DCM. The combined organic layer was washed with saturated aqueous $NaHCO_3$ solution once. The organic layer was dried over Na_2SO_4 and filtered. Volatiles were removed under reduced pressure and the crude product was purified by column chromatography using silica gel (typical eluent = heptane : EtOAc – 95 : 5 → 20 : 80 v/v%) to obtain the pure amide. Often a simple filtration of a solution of the amide in EtOAc was sufficient to yield the amide in sufficient purity.

Procedure B (Enantiopure starting material)

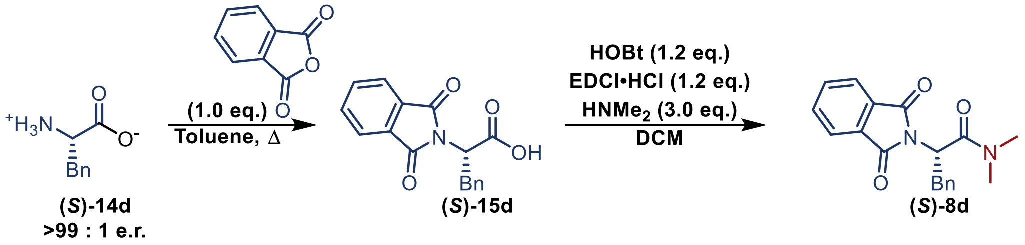

Scheme S 2

Enantiopure (*S*)-phenylalanine (1.0 eq., 10 mmol, 1.65 g) was suspended in toluene (20 mL) and phthalic anhydride (1.0 eq., 10 mmol, 1.48 g) was added to the mixture. The reaction was heated to reflux for 4 h, with a Dean-Stark apparatus attached. After cooling down, the percipitate was filtered,washed with toluene (3x10 mL) and dried on a vacuum pump to obtain the product as a white to yellowish crystalline solid ((*S*)-15d; yield 89%, 2.64 g).

Dimethylamine in THF (2 M, 3.0 eq., 3 mL) was diluted with DCM (27 mL) and cooled to 0 °C. **1-**Hydroxybenzotriazole hydrate (1.2 eq., 2.4 mmol, 324 mg) and 1-(3-Dimethylaminopropyl)-3-ethylcarbodiimide Hydrochloride (EDCI•HCl, 1.2 eq., 2.4 mmol, 460 mg) were added, followed by the enantiopure acid **15d** (1.0 eq., 2.0 mmol, 591 mg). The reaction was stirred at room temperature for 14 h,after which it was quenched with aqueous HCl (1 M, 20 mL) and then washed with a saturated aqueous $NaHCO_3$ solution (20 mL). The organic

phase was dried over dried over Na_2SO_4 and filtered. Volatiles were removed under reduced pressure and the crude product was purified by column chromatography using silica gel (eluent = heptane : EtOAc – 95 : 5 → 20 : 80 v/v%) to obtain the pure amide (yield 79%, 510 mg).

III. Formation of 7-membered heterocycles

Procedure C

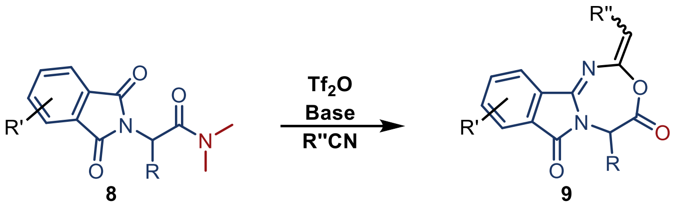

8 **9**

The α-phthalimido amide (1.0 eq., 0.20 mmol) was dissolved in the nitrile (1 mL) and cooled to 0 °C. The base was added, followed by trifluoromethanesulfonic anhydride. The reaction was then stirred at room temperature for a given amount of time (see characterization section for stoichiometry and reaction time). Then, water (2 - 3 mL) and DCM (2 – 3 mL) were added to the mixture, resulting in a strong change in color. The aqueous phase was extracted with DCM (3 x 5 mL) and the united organic layers were dried over Na_2SO_4 and filtered. Volatiles were removed under reduced pressure and the crude product was purified by column chromatography using silica gel or Florisil® (eluent = heptane : EtOAc – 95 : 5 → 20 : 80 v/v%) to obtain the desired product usually as a shiny yellow solid.

^{18}O labeling experiment

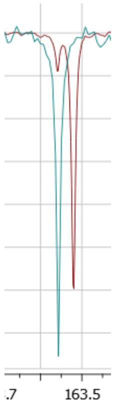

Figure S 1

The reaction with $H_2{}^{18}O$ as the quenching reagent (*Scheme 4a*) was set up in an analogy to **procedure C** only that 0.5 mL of the isotopically labelled water was used. In *Figure S 1* the peak of the carbonyl attached to the ^{18}O is shown in the ^{13}C NMR spectrum of compound **9d**. The blue trace shows the peak of the non-isotopically labeled isomer. *Figure S 2* shows the ratio found by mass spectrometry.

With a relative intensity of [^{18}O]-**9d**/([^{18}O]-**9d**+[^{16}O]-**9d**) = 100.0/110.5 = 90.5% for the ^{18}O labeled compound in the MS spectrum (*Figure S 2* – data points marked in red).

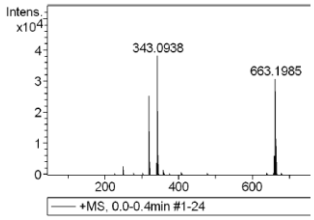

#	m/z	I	I %
1	249.1017	2769	7.3
2	250.1048	467	1.2
3	279.1006	545	1.4
4	304.2606	459	1.2
5	319.1072	2967	7.8
6	320.1105	683	1.8
7	321.1117	25258	66.2
8	322.1147	4151	10.9
9	323.1171	470	1.2
10	341.0891	4009	10.5
11	342.0924	975	2.6
12	343.0938	38143	100.0

Use of enantiopure substrate (*S*)-8d

The enantiopure sample gave a >99 : 1 e.r., which is the same as the amino acid starting material provided by TCI. The enantiomers of product **9d** can be separated using a "Chiralpak IH-3 150x4.6 mm" HPLC column with the following parameters:

Eluent = *n*-heptane : EtOH : IPA - 79.95 : 19.95 : 0.10 v/v%)
Flow = 1 mL/min
T = 25 °C

Chromatogram racemate:

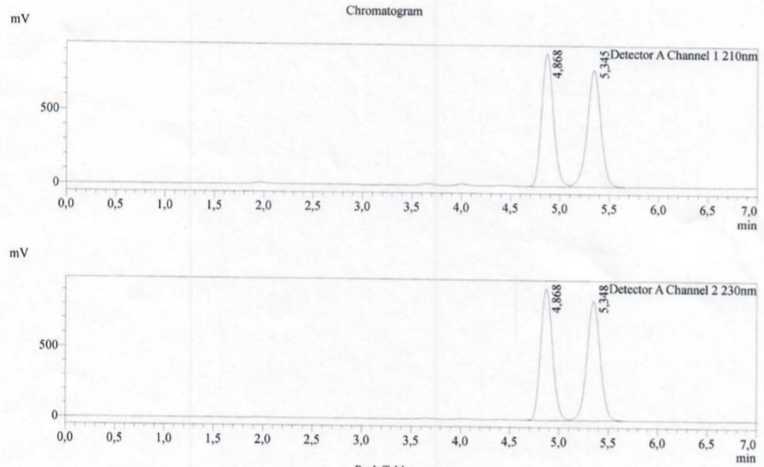

Peak Table

Detector A Channel 1 210nm

Peak#	Ret. Time	Area	Area%
1	4,868	7581541	49,858
2	5,345	7624633	50,142
Total		15206174	100,000

Detector A Channel 2 230nm

Peak#	Ret. Time	Area	Area%
1	4,868	7916555	49,752
2	5,348	7995582	50,248
Total		15912137	100,000

Chromatogram enantiopure sample

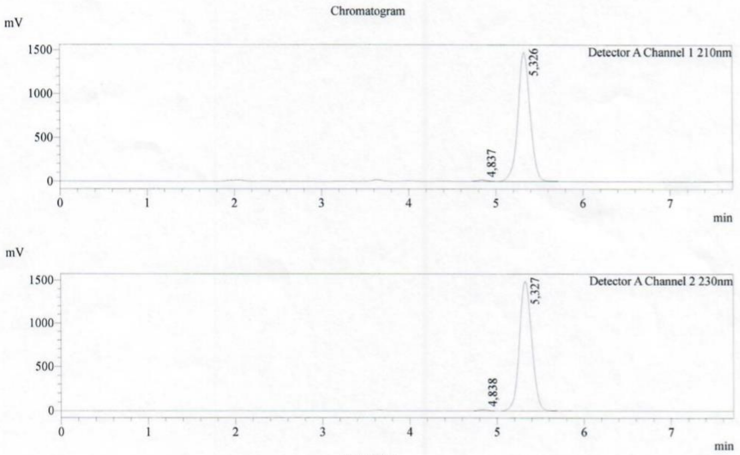

Peak Table

Detector A Channel 1 210nm

Peak#	Ret. Time	Area	Area%
1	4,837	121615	0,834
2	5,326	14452988	99,166
Total		14574603	100,000

Detector A Channel 2 230nm

Peak#	Ret. Time	Area	Area%
1	4,838	136311	0,916
2	5,327	14743007	99,084
Total		14879318	100,000

Further evidence for an iminium species

The proposed iminium species in the mechanism (scheme 4c) was also consistent with the ^{1}H NMR spectrum of a crude reaction mixture which was not treated with water at the end but where the volatiles were just evaporated under reduced pressure. Figure S3 shows the

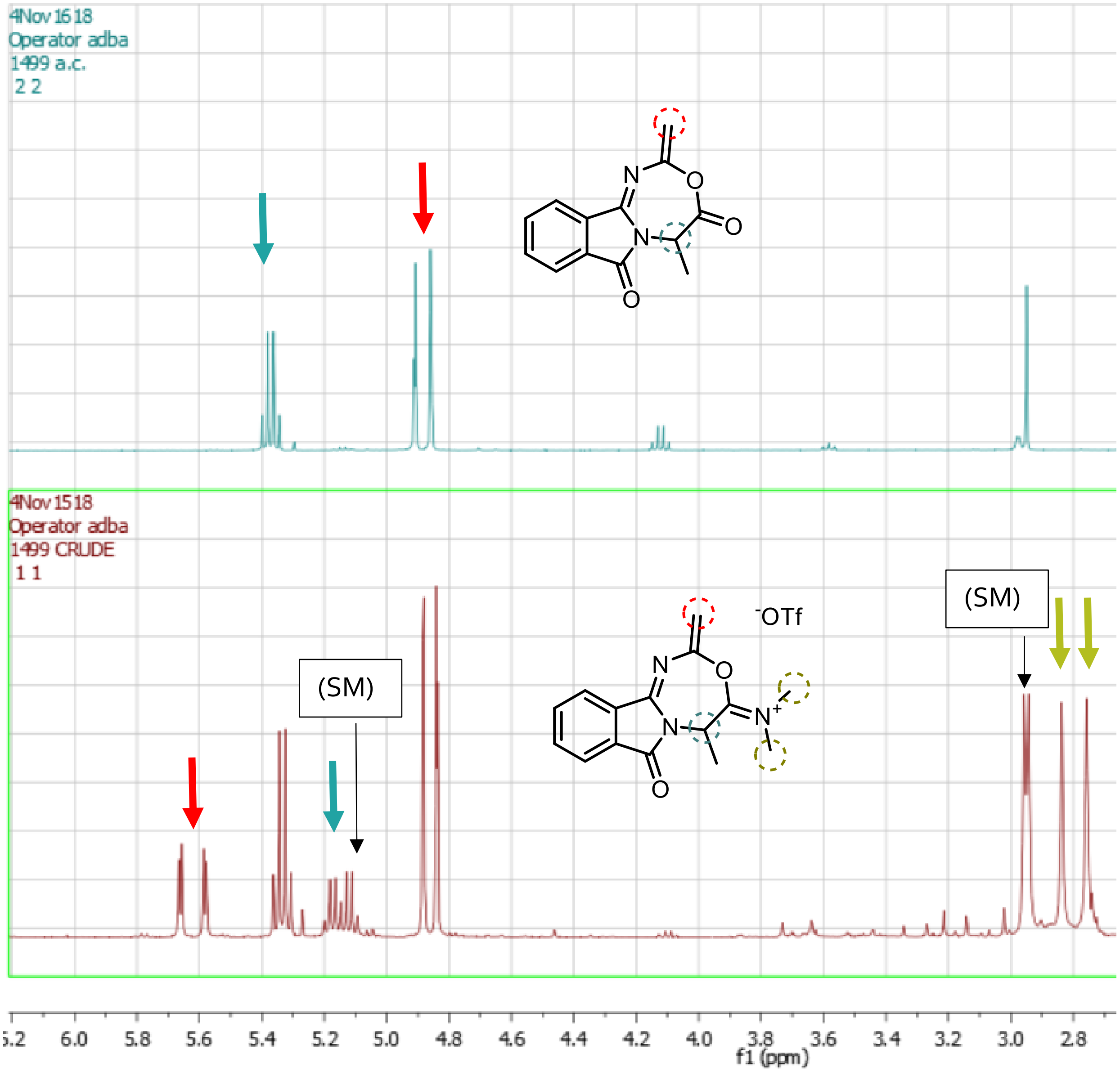
4Nov 16 18
Operator adba
1499 a.c.
2 2
4Nov 15 18
Operator adba
1499 CRUDE
1 1
-OTf
(SM)
(SM)
f1 (ppm)

Major isomer assignment of 9m/9m'

The major isomer of **9m/9m'** was determined by 2D NMR analysis. The HMBC spectrum showed a pronounced ^3J coupling between the "amide" carbonyl and the highlighted *ortho* proton:

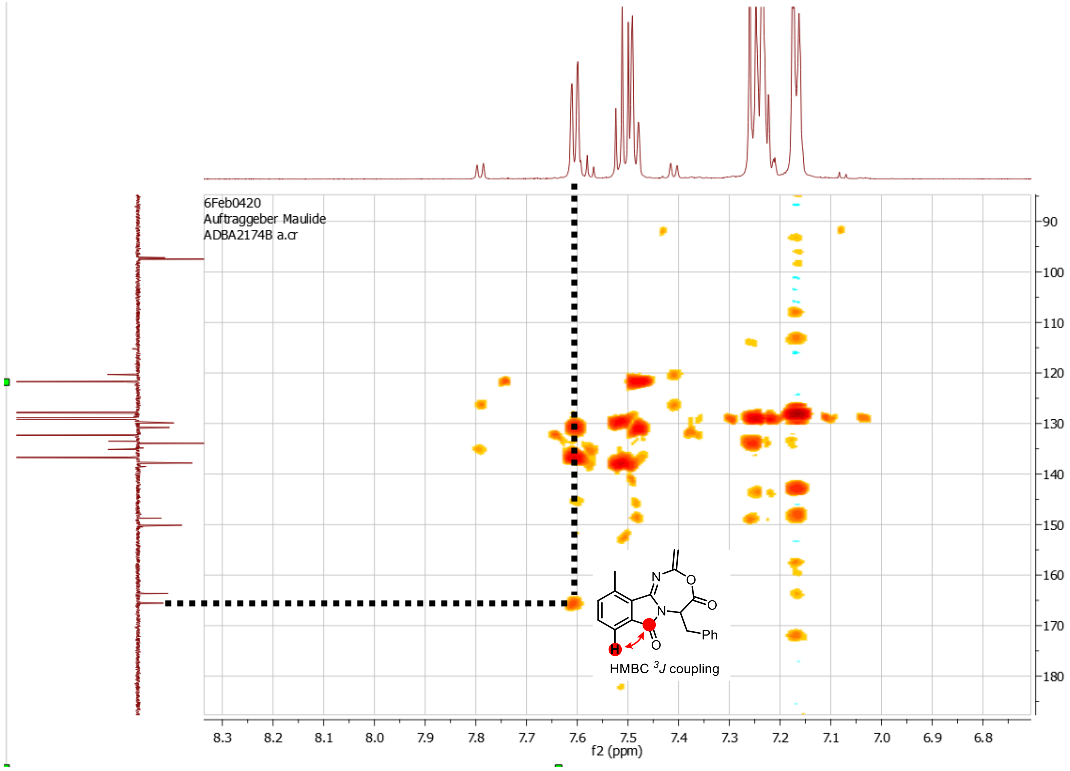

This is also consistent with the fact that that the unsubstituted cognate shows the same coupling with the less deshielded proton, while the "amidine" carbon couples strongly to the other "*ortho*" proton:

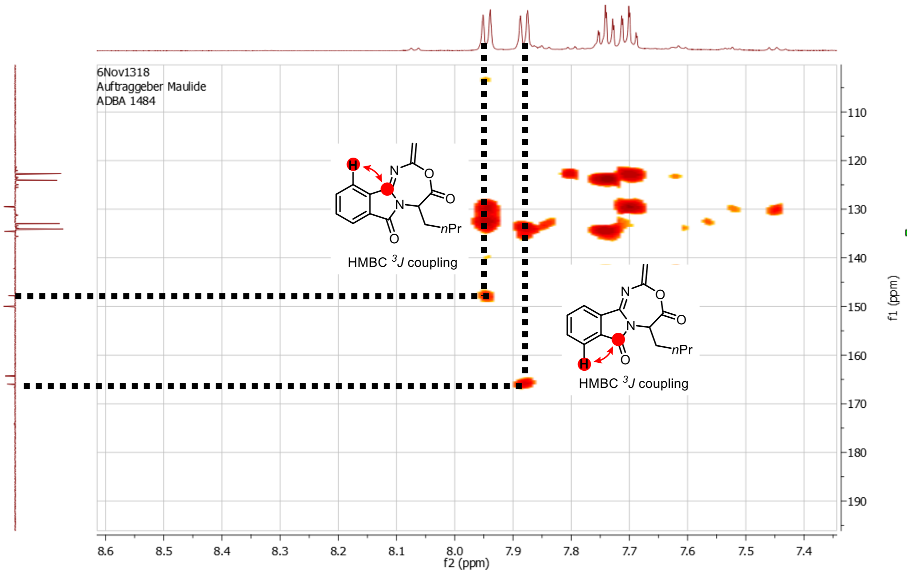

IV. Characterization

2-(1,3-dioxoisoindolin-2-yl)-*N*,*N*-dimethylhexanamide (**8a**)

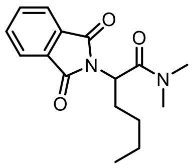

Synthesized following the general procedure **A**.

Isolated yield: 1445 mg, 66%. ^{1}**H NMR** (700 MHz, $CDCl_3$) δ 7.91 – 7.76 (m, 2H), 7.76 – 7.69 (m, 2H), 5.02 (dd, *J* = 10.6, 4.8 Hz, 1H), 3.00 (s, 3H), 2.95 (s, 3H), 2.52 – 2.43 (m, 1H), 2.07 – 1.97 (m, 1H), 1.42 – 1.21 (m, 4H), 0.87 (t, *J* = 6.8 Hz, 3H). ^{13}**C NMR** (176 MHz, $CDCl_3$) δ 169.04, 168.21 (2xC), 134.21 (2xC), 131.83 (2xC), 123.56 (2xC), 52.02, 37.17, 36.29, 28.82, 28.48, 22.33, 14.04. **HRMS (ESI)** exact mass calculated for $[M+Na]^+$ requires 311.3368 m/z found 311.3360 m/z. **ATR-FTIR** (cm^{-1}): 2956, 2930, 2860, 1777, 1709, 1654, 1612, 1495, 1467, 1381, 1358, 1336, 1288, 1261, 1191, 1171, 1129, 1111, 1070, 951, 882, 791, 718, 698, 622.

2-(1,3-dioxoisoindolin-2-yl)-*N*,*N*-dimethylpropanamide (**8b**)

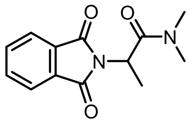

Synthesized following the general procedure **A**.

Isolated yield: 430 mg, 35%. ^{1}**H NMR** (600 MHz, $CDCl_3$) δ 7.87 – 7.82 (m, 2H), 7.75 – 7.69 (m, 2H), 5.14 (q, *J* = 7.2 Hz, 1H), 2.98 (s, 3H), 2.97 (s, 3H), 1.71 (d, *J* = 7.2 Hz, 3H). ^{13}**C NMR** (151 MHz, $CDCl_3$) δ 169.15 (2xC), 167.89 (2xC), 134.25 (2xC), 131.93 (2xC), 123.59 (2xC), 47.33, 37.12, 36.37, 15.58. **HRMS** (ESI^+): exact mass calculated for $[M+Na]^+$ requires 269.0897 m/z, found 269.0900 m/z. **ATR-FTIR** (cm^{-1}): 2935, 1778, 1713, 1657, 1612, 1501, 1468, 1385, 1260, 1174, 1130, 1085, 1052, 1018, 896, 881, 721.

2-(1,3-dioxoisoindolin-2-yl)-*N*,*N*,3-trimethylbutanamide (**8c**)

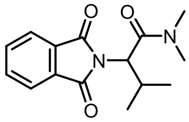

Synthesized following the general procedure **A**.

Isolated yield: 320 mg, 11%. ^{1}**H NMR** (600 MHz, $CDCl_3$) δ 7.84 (dd, *J* = 5.3, 3.1 Hz, 2H), 7.72 (dd, *J* = 5.4, 3.0 Hz, 2H), 4.68 (d, *J* = 10.2 Hz, 1H), 3.19 – 3.07 (m, 1H), 3.03 (s, 3H), 2.95 (s, 3H), 1.05 (d, *J* = 6.6 Hz, 3H), 0.88 (d, *J* = 6.7 Hz, 3H). ^{13}**C NMR** (151 MHz, $CDCl_3$) δ 168.39, 167.98 (2xC), 134.32 (2xC), 131.60 (2xC), 123.64 (2xC), 56.78, 37.38, 36.24, 27.80, 20.53, 19.35. **HRMS (ESI):** exact mass calculated for $[M+Na]^+$ requires 297.1216 m/z, found 297.1210 m/z. **ATR-FTIR** (cm^{-1}): 2964, 2932, 1770, 1710, 1652, 1613, 1494, 1468, 1379, 1354, 1331, 1269, 1254, 1174, 1132, 1107, 1069, 989, 917, 892, 794, 752, 719, 698, 665, 635.

2-(1,3-dioxoisoindolin-2-yl)-*N*,*N*-dimethyl-3-phenylpropanamide (**8d**)

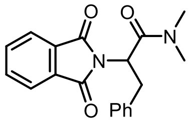

Synthesized following the general procedure **A**.

Isolated yield: 895 mg, 78%. **^{1}H NMR** (400 MHz, $CDCl_3$) δ 7.85 – 7.75 (m, 2H), 7.74 – 7.65 (m, 2H), 7.25 – 7.09 (m, 5H), 5.29 (dd, *J* = 10.0, 5.8 Hz, 1H), 3.65 (dd, *J* = 14.2, 10.1 Hz, 1H), 3.48 (dd, *J* = 14.2, 5.8 Hz, 1H), 2.97 (s, 3H), 2.93 (s, 3H). ^{13}C NMR (101 MHz, $CDCl_3$) δ 168.34, 167.85 (2xC), 137.34, 134.23 (2xC), 131.63 (2xC), 129.27 (2xC), 128.65 (2xC), 126.95, 123.57 (2xC), 53.06, 37.16, 36.41, 35.11. HRMS (ESI) m/z calculated for [M+H]+ 323.1390 found 323.1388. **HRMS (ESI):** exact mass calculated for $[M+Na]^+$ requires 323.1390 m/z, found 323.1388 m/z. **ATR-FTIR** (cm^{-1})**:** 3062, 3028, 2933, 1774, 1711, 1656, 1612, 1496, 1468, 1455, 1381, 1361, 1336, 1258, 1187, 1173, 1141, 1101, 993, 957, 916, 891, 877, 788, 751, 720, 700, 666, 624.

3-(4-bromophenyl)-2-(1,3-dioxoisoindolin-2-yl)-*N,N*-dimethylpropanamide (**8e**)

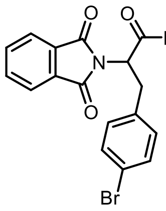

Synthesized following the general procedure **A**.

Isolated yield: 602 mg, 73%. **^{1}H NMR** (400 MHz, $CDCl_3$) δ 7.79 (dd, *J* = 5.5, 3.1 Hz, 2H), 7.70 (dd, *J* = 5.5, 3.0 Hz, 2H), 7.32 (d, *J* = 8.4 Hz, 2H), 7.09 (d, *J* = 8.3 Hz, 2H), 5.25 (dd, *J* = 9.9, 5.9 Hz, 1H), 3.58 (dd, *J* = 14.3, 9.9 Hz, 1H), 3.43 (dd, *J* = 14.3, 5.9 Hz, 1H), 2.96 (s, 3H), 2.94 (s, 3H). **^{13}C NMR** (101 MHz, $CDCl_3$) δ 167.92, 167.74 (2xC), 136.34 (2xC), 134.39 (2xC), 131.75 (2xC), 131.49 (2xC), 131.05 (2xC), 123.69 (2xC), 120.90, 52.60, 37.15, 36.45, 34.64. **HRMS (ESI):** exact mass calculated for $[M+Na]^+$ requires 423.0315 m/z, found 423.0325 m/z. **ATR-FTIR** (cm^{-1})**:** 3011, 2933, 1775, 1713, 1656, 1613, 1488, 1468, 1381, 1359, 1335, 1259, 1216, 1173, 1141, 1108, 1085, 1071, 1012, 993, 958, 880, 863, 808, 789, 749, 718, 698, 666, 645, 611, 590.

2-(1,3-dioxoisoindolin-2-yl)-3-(4-methoxyphenyl)-*N,N*-dimethylpropanamide (**8f**)

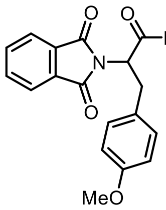

Synthesized following the general procedure **A**.

Isolated yield: 692 mg, 61%. **^{1}H NMR** (600 MHz, $CDCl_3$) δ 7.78 (dd, *J* = 5.4, 3.1 Hz, 2H), 7.68 (dd, *J* = 5.4, 3.0 Hz, 2H), 7.12 (d, *J* = 8.5 Hz, 2H), 6.73 (d, *J* = 8.5 Hz, 2H), 5.25 (dd, *J* = 10.0, 5.9 Hz, 1H), 3.72 (s, 3H), 3.59 (dd, *J* = 14.4, 10.0 Hz, 1H), 3.42 (dd, *J* = 14.4, 5.9 Hz, 1H), 2.96 (s, 3H), 2.94 (s, 3H). **^{13}C NMR** (151 MHz, $CDCl_3$) δ 168.45, 167.90 (2xC), 158.53, 134.21 (2xC), 131.66, 130.26 (2xC), 129.27, 123.57 (2xC), 114.05 (2xC), 55.30, 53.20, 37.18, 36.39, 34.20. **HRMS (ESI):** exact mass calculated for $[M+Na]^+$ requires 375.1315 m/z, found 375.1318 m/z. **ATR-FTIR** (cm^{-1})**:** 2942, 2165, 2141, 1989, 1774, 1715, 1658, 1612, 1513, 1468, 1384, 1336, 1302, 1248, 1179, 1143, 1115, 1087, 1034, 877, 817.

2-(1,3-dioxoisoindolin-2-yl)-*N,N*-dimethylpent-4-enamide (**8g**)

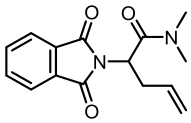

Synthesized following the general procedure **A**.

Isolated yield: 468 mg, 53%. **^{1}H NMR** (600 MHz, $CDCl_3$) δ 7.83 (dd, *J* = 5.3, 3.1 Hz, 2H), 7.71 (dd, *J* = 5.4, 3.0 Hz, 2H), 5.83 – 5.71 (m, 1H), 5.12 – 5.05 (m, 2H), 4.98 (d, *J* = 10.1 Hz, 1H), 3.14 (dt, *J* = 14.7, 9.6 Hz, 1H), 2.96 (s, *J* = 5.5 Hz, 3H), 2.95 (s, 3H), 2.78 (dt, *J* = 14.6, 5.2 Hz, 1H). **^{13}C NMR** (151 MHz, $CDCl_3$) δ 168.17, 167.85 (2xC), 134.27 (2xC), 133.78, 131.65 (2xC), 123.60 (2xC), 118.75, 51.23, 37.12, 36.35, 33.53. **HRMS (ESI):** exact mass calculated for $[M+Na]^+$ requires 295.1053 m/z, found 295.1059 m/z. **ATR-FTIR** (cm^{-1})**:** 2927, 1775, 1710, 1655, 1495, 1468, 1438, 1380, 1356, 1336, 1258, 1190, 1169, 1125, 1077, 994, 956, 922, 880, 841, 794, 752, 718, 665, 619.

2-(5-(*tert*-butyl)-1,3-dioxoisoindolin-2-yl)-*N,N*-dimethyl-3-phenylpropanamide (**8k**)

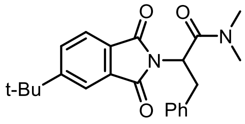

Synthesized following the general procedure **A**.

Isolated yield: 1280 mg, 44%. **^{1}H NMR** (600 MHz, $CDCl_3$) δ 7.80 (s, 1H), 7.68 (s, 2H), 7.23 – 7.10 (m, 5H), 5.26 (dd, *J* = 10.0, 5.7 Hz, 1H), 3.62 (dd, *J* = 14.3, 10.1 Hz, 1H), 3.47 (dd, *J* = 14.3, 5.7 Hz, 1H), 2.95 (s, 3H), 2.92 (s, 3H), 1.34 (s, 9H). **^{13}C NMR** (151 MHz, $CDCl_3$) δ 168.35, 168.21, 167.77, 158.79, 137.38, 131.72, 131.19, 129.21 (2xC), 128.82, 128.54 (2xC), 126.81, 123.30, 120.67, 52.89, 37.07, 36.33, 35.78, 35.09, 31.17. **HRMS (ESI):** exact mass calculated for $[M+Na]^+$ requires 401.1846 m/z, found 401.1836 m/z. **ATR-FTIR** (cm^{-1})**:** 3014, 2964, 1772, 1711, 1655, 1620, 1494, 1455, 1430, 1373, 1257, 1216, 1181, 1141, 1098, 1076, 1030, 998, 961, 908, 850, 829, 746, 694, 666, 634, 321, 594.

2-(5-bromo-1,3-dioxoisoindolin-2-yl)-*N,N*-dimethyl-3-phenylpropanamide (**8l**)

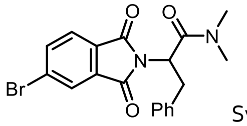

Synthesized following the general procedure **A**.

Isolated yield: 416 mg, 39%. **^{1}H NMR** (600 MHz, $CDCl_3$) δ 7.90 (s, 1H), 7.84 – 7.78 (m, 1H), 7.63 (d, *J* = 7.9 Hz, 1H), 7.23 – 7.12 (m, 5H), 5.29 (dd, *J* = 10.3, 5.7 Hz, 1H), 3.67 (dd, *J* = 14.3, 10.4 Hz, 1H), 3.45 (dd, *J* = 14.3, 5.6 Hz, 1H), 2.96 (s, 3H), 2.94 (s, 3H). **^{13}C NMR** (151 MHz, $CDCl_3$) δ 168.10, 167.05, 166.50, 137.22, 137.05, 133.20, 130.10, 129.15, 129.13, 128.68 (2xC), 127.03 (2xC), 126.86, 124.89, 53.31, 37.15, 36.37, 34.84. **HRMS (ESI):** exact mass calculated for $[M+Na]^+$ requires 423.0325 m/z, found 423.0326 m/z. **ATR-FTIR** (cm^{-1})**:** 3064, 3015, 2934, 1775, 1713, 1653, 1605, 1495, 1455, 1418, 1399, 1371, 1256, 1216, 1188, 1169, 1141, 1099, 1075, 1049, 995, 957, 894, 874, 843, 792, 739, 697, 664, 622, 597.

N,N-dimethyl-2-(4-methyl-1,3-dioxoisoindolin-2-yl)-3-phenylpropanamide (**8m**)

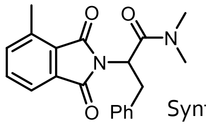

Synthesized following the general procedure **A**.

Isolated yield: 654 mg, 80%. **^{1}H NMR** (600 MHz, $CDCl_3$) δ 7.60 (d, *J* = 7.3 Hz, 1H), 7.52 (dd, *J* = 9.4, 5.6 Hz, 1H), 7.42 (d, *J* = 7.7 Hz, 1H), 7.24 – 7.10 (m, 5H), 5.27 (dd, *J* = 9.7, 6.0 Hz, 1H), 3.62 (dd, *J* = 14.2, 9.7 Hz, 1H), 3.49 (dd, *J* = 14.2, 6.0 Hz, 1H), 2.96 (s, 3H), 2.92 (s, 3H), 2.63 (s, 3H). **^{13}C NMR** (151 MHz, $CDCl_3$) δ 168.61, 168.41, 167.83, 138.23, 137.48, 136.61, 133.69, 132.04, 129.28 (2xC), 128.58 (2xC), 128.26, 126.85, 121.18, 52.82, 37.10, 36.36, 35.16, 17.63. **HRMS (ESI):** exact mass calculated for $[M+Na]^+$ requires 359.1380 m/z, found 359.1366 m/z. **ATR-FTIR** (cm^{-1})**:** 3063, 3010, 2929, 1768, 1707, 1652, 1620, 1604, 1495, 1455, 1381, 1338, 1260, 1234, 1217, 1177, 1141, 1101, 1072, 1012, 990, 958, 933, 886, 874, 874, 833, 816, 788, 737, 699, 666, 634, 618.

5-butyl-2-methylene-[1,3,5]oxadiazepino[4,5-*a*]isoindole-4,7(2*H*,5*H*)-dione (**9a**)

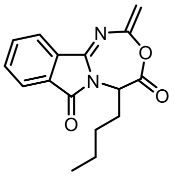

Synthesized following the general procedure **C**. (Reaction time = 0.5 h. 1.1 eq. Tf_2O used (0.22 mmol, 37 µL). Base = DTBP (2.2 eq., 0.44 mmol, 99 µL). SiO_2 used for purification)

Isolated yield: 28.3 mg, 50%. **^{1}H NMR** (600 MHz, $CDCl_3$) δ 7.97 – 7.93 (m, 1H), 7.90 – 7.87 (m, 1H), 7.76 – 7.72 (m, 1H), 7.72 – 7.68 (m, 1H), 5.21 (dd, *J* = 8.7, 8.2 Hz, 1H), 4.88 (d, *J* = 1.6 Hz, 1H), 4.83 (d, *J* = 1.6 Hz, 1H), 2.12 – 1.98 (m, 1H), 1.95 – 1.83 (m, 1H), 1.40 – 1.32 (m, 4H), 0.89 – 0.85 (m,3H). **^{13}C NMR** (151 MHz, $CDCl_3$) δ 166.11, 164.46, 150.14, 147.95, 134.75, 134.22, 133.09, 129.63, 124.20, 122.85, 97.08, 56.39, 32.43, 27.64, 22.08, 13.79. **HRMS (ESI)** exact mass calculated for $[M+Na]^+$ requires 307.1059 m/z

found 307.1054 m/z. **ATR-FTIR** (cm^{-1}): 2924, 2854, 1742, 1716, 1658, 1469, 1389, 1357, 1295, 1227, 1175, 1142, 1062, 1017, 1006, 978, 930, 917, 895, 881, 848, 778, 704, 653, 633.

5-methyl-2-methylene-[1,3,5]oxadiazepino[4,5-*a*]isoindole-4,7(2*H*,5*H*)-dione (**9b**)

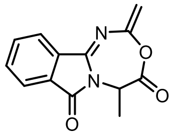

Synthesized following the general procedure **C**. (Reaction time = 0.5 h. 1.1 eq. Tf_2O used (0.22 mmol, 37 µL). Base = DTBP (2.2 eq., 0.44 mmol, 99 µL). SiO_2 used for purification)

Isolated yield: 23.0 mg, 48%. **^{1}H NMR** (400 MHz, $CDCl_3$): δ 7.91 – 7.86 (m, 1H), 7.81 (dd, *J* = 6.4, 1.0 Hz, 1H), 7.72 – 7.60 (m, 2H), 5.30 (q, *J* = 7.4 Hz, 1H), 4.81 (dd, *J* = 20.7, 1.5 Hz, 2H), 1.62 (d, *J* = 7.4 Hz, 3H). **^{13}C NMR** (151 MHz, $CDCl_3$): δ 165.69, 164.77, 150.03, 147.73, 134.65, 134.09, 132.95, 129.60, 123.99, 122.72, 97.15, 51.73, 18.51. **HRMS (ESI)** exact mass calculated for $[M+Na]^+$ requires 243.0770 m/z; found 243.0761 m/z. **ATR-FTIR** (cm^{-1}): 2924, 2853, 1768, 1742, 1657, 1626, 1611, 1471, 1452, 1388, 1353, 1293, 1264, 1227, 1174, 1141, 1062, 1017, 1006, 977, 932, 917, 895, 854, 797, 778, 730, 703, 674, 655, 610.

5-isopropyl-2-methylene-[1,3,5]oxadiazepino[4,5-*a*]isoindole-4,7(2*H*,5*H*)-dione (**9c**)

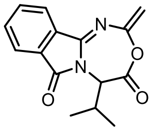

Synthesized following the general procedure **C**. (Reaction time = 0.5 h. 1.1 eq. Tf_2O used (0.22 mmol, 37 µL). Base = DTBP (2.2 eq., 0.44 mmol, 99 µL). Florisil® used for purification)

Isolated yield: 17.9 mg, 33%. **^{1}H NMR** (400 MHz, $CDCl_3$) δ 7.95 (d, *J* = 7.0 Hz, 1H), 7.89 (d, *J* = 6.9 Hz, 1H), 7.79 – 7.66 (m, 2H), 4.88 (d, *J* = 1.3 Hz, 1H), 4.84 – 4.77 (m, 2H), 2.43 – 2.29 (m, 1H), 1.16 (d, *J* = 6.7 Hz, 3H), 0.87 (d, *J* = 6.6 Hz, 3H). **^{13}C NMR** (101 MHz, $CDCl_3$) δ 166.35, 164.03, 150.10, 148.03, 134.62, 134.25, 133.12, 129.51, 124.30, 122.87, 96.87, 62.81, 31.74, 19.41, 18.76. **HRMS (ESI):** exact mass calculated for $[M+Na]^+$ requires 271.1077 m/z, found 271.1078 m/z. **ATR-FTIR** (cm^{-1}): 2968, 2935, 1743, 1657, 1628, 1612, 1470, 1386, 1356, 1293, 1258, 1220, 1169, 1111, 1084, 1021, 1001, 963, 938, 912, 863, 774, 726, 708, 671, 648, 616.

5-benzyl-2-methylene-[1,3,5]oxadiazepino[4,5-*a*]isoindole-4,7(2*H*,5*H*)-dione (**9d**)

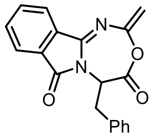

Synthesized following the general procedure **C**. (Reaction time = 0.5 h. 1.1 eq. Tf_2O used (0.22 mmol, 37 µL). Base = DTBP (2.2 eq., 0.44 mmol, 99 µL). SiO_2 used for purification)

Isolated yield: 46.1 mg, 72%. **^{1}H NMR** (400 MHz, $CDCl_3$) δ 7.97 (d, *J* = 7.4 Hz, 1H), 7.78 – 7.71 (m, 2H), 7.70 – 7.63 (m, 1H), 7.25 – 7.20 (m, 3H), 7.18 – 7.12 (m, 2H), 5.44 (dd, *J* = 9.3, 8.0 Hz, 1H), 5.00 (d, *J* = 1.4 Hz, 1H), 4.97 (d, *J* = 1.4 Hz, 1H), 3.42 (dd, *J* = 13.7, 8.0 Hz, 1H), 3.21 (dd, *J* = 13.7, 9.3 Hz, 1H). **^{13}C NMR** (101 MHz, $CDCl_3$) δ 165.53, 163.59, 150.41, 147.91, 134.59, 134.17, 133.88, 133.10, 129.46 (2xC), 129.24 (2xC), 129.05, 127.98, 124.12, 122.85, 97.83, 57.73, 38.72. 1072 **HRMS (ESI):** exact mass calculated for $[M+H]^+$ requires 319.1080 m/z, found 319.1077 m/z. **ATR-FTIR** (cm^{-1}): 3031, 2928, 1771, 1743, 1656, 1622, 1610, 1497, 1470, 1456, 1387, 1354, 1307, 1292, 1260, 1220, 1181, 1158, 1143, 1105, 1091, 1077, 1032, 1002, 981, 942, 925, 889, 848, 796, 777, 752, 723, 698, 671, 665, 609.

5-(4-bromobenzyl)-2-methylene-[1,3,5]oxadiazepino[4,5-*a*]isoindole-4,7(2*H*,5*H*)-dione (**9e**)

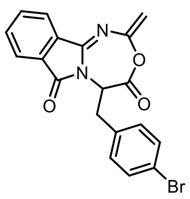

Synthesized following the general procedure **C**. (Reaction time = 0.5 h. 1.1 eq. Tf_2O used (0.22 mmol, 37 µL). Base = DTBP (2.2 eq., 0.44 mmol, 99 µL))

Isolated yield: 29.5 mg, 37%. **^{1}H NMR** (400 MHz, $CDCl_3$) δ 7.96 (d, *J* = 7.4 Hz, 1H), 7.83 – 7.65 (m, 3H), 7.36 (d, *J* = 8.3 Hz, 2H), 7.03 (d, *J* = 8.3 Hz, 2H), 5.42 (dd, *J* = 9.1, 8.1 Hz, 1H), 4.99 (d, *J* = 1.4 Hz, 1H), 4.96 (d, *J* = 1.4 Hz, 1H), 3.36 (dd, *J* = 13.8, 7.9 Hz, 1H), 3.17 (dd, *J* = 13.8, 9.3 Hz, 1H). **^{13}C NMR (**101 MHz, $CDCl_3$) δ 165.53, 163.30, 150.27, 147.82, 134.49, 134.31, 133.24, 132.86, 132.22 (2xC), 130.90 (2xC), 129.33, 124.26, 122.91, 122.13, 97.86, 57.34, 38.17. **HRMS (ESI):** exact mass calculated for $[M\text{-}C_2\text{+}Na]^+$ requires 395.0002 m/z, found 394.9996 m/z. **ATR-FTIR** (cm^{-1}): 3185, 3025, 2955, 2867, 1716, 1672, 1591, 1576, 1488, 1470, 1387, 1217, 1146, 1116, 1091, 1071, 1012, 954, 883, 815, 760, 720, 707.

5-(4-methoxybenzyl)-2-methylene-[1,3,5]oxadiazepino[4,5-*a*]isoindole-4,7(2*H*,5*H*)-dione (**9f**)

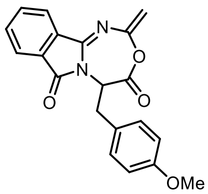

Synthesized following the general procedure **C**. (Reaction time = 0.5 h. 1.1 eq. Tf_2O used (0.22 mmol, 37 µL). Base = DTBP (2.2 eq., 0.44 mmol, 99 µL). SiO_2 used for purification))

Isolated yield: 30.3 mg, 44%. **^{1}H NMR** (600 MHz, $CDCl_3$) δ 7.96 (d, *J* = 7.5 Hz, 1H), 7.77 (d, *J* = 7.5 Hz, 1H), 7.73 (td, *J* = 7.5, 0.9 Hz, 1H), 7.66 (td, *J* = 7.5, 0.8 Hz, 1H), 7.06 (d, *J* = 8.6 Hz, 2H), 6.76 (d, *J* = 8.6 Hz, 2H), 5.40 (dd, *J* = 9.1, 8.2 Hz, 1H), 4.99 (d, *J* = 1.5 Hz, 1H), 4.96 (d, *J* = 1.5 Hz, 1H), 3.74 (s, 3H), 3.35 (dd, *J* = 13.9, 8.1 Hz, 1H), 3.15 (dd, *J* = 13.9, 9.3 Hz, 1H). **^{13}C NMR** (151 MHz, $CDCl_3$) δ 165.56, 163.69, 159.26, 150.41, 147.90, 134.59, 134.15, 133.07, 130.29 (2xC), 129.48, 125.75, 124.13, 122.82, 114.44 (2xC), 97.74, 57.85, 55.32, 37.86. **HRMS (ESI+):** exact mass calculated for $[M\text{+}Na]^+$ requires 371.1002 m/z, found 371.0992 m/z. **ATR-FTIR** (cm^{-1}): 3038, 3001, 2956, 2933, 2837, 1743, 1657, 1611, 1586, 1513, 1470, 1443, 1354, 1301, 1248, 1222, 1179, 1165, 1147, 1114, 1089, 1032, 1003, 980, 938, 911.

5-allyl-2-methylene-[1,3,5]oxadiazepino[4,5-*a*]isoindole-4,7(2*H*,5*H*)-dione (**9g**)

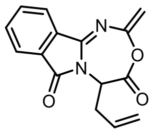

Synthesized following the general procedure **C**. (Reaction time = 0.5 h. 1.1 eq. Tf_2O used (0.22 mmol, 37 µL). Base = DTBP (2.2 eq., 0.44 mmol, 99 µL). SiO_2 used for purification)

Isolated yield: 28.3 mg, 54%. **^{1}H NMR** (600 MHz, $CDCl_3$) δ 7.94 (d, *J* = 7.4 Hz, 1H), 7.86 (d, *J* = 7.4 Hz, 1H), 7.74 (dt, *J* = 7.4, 3.8 Hz, 1H), 7.70 (dd, *J* = 10.8, 4.0 Hz, 1H), 5.71 (ddt, *J* = 17.1, 10.1, 7.2 Hz, 1H), 5.29 (dd, *J* = 9.4, 7.7 Hz, 1H), 5.14 – 5.09 (m, 2H), 4.91 (d, *J* = 1.4 Hz, 1H), 4.88 (d, *J* = 1.4 Hz, 1H), 2.90 – 2.81 (m, 1H), 2.74 – 2.63 (m, 1H). **^{13}C NMR** (151 MHz, $CDCl_3$) δ 166.02, 163.78, 150.08, 147.76, 134.63, 134.22, 133.10, 130.59, 129.60, 124.20, 122.87, 120.91, 97.55, 55.96, 37.17. **HRMS (ESI):** exact mass calculated for $[M\text{+}Na]^+$ requires 291.0740 m/z, found 291.0742 m/z. **ATR-FTIR** (cm^{-1})**:** 3081, 2927, 1744, 1658, 1625, 1612, 1471, 1440, 1388, 1356, 1293, 1267, 1224, 1168, 1134, 1084, 1020, 1005, 976, 934, 859, 795, 777, 729, 706, 672, 664, 612.

5-benzyl-2-propylidene-[1,3,5]oxadiazepino[4,5-*a*]isoindole-4,7(2*H*,5*H*)-dione (**9j**)

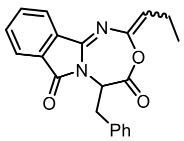

Synthesized following the general procedure **C**. (Reaction time = 0.5 h. 1.1 eq. Tf_2O used (0.22 mmol, 37 µL). Base = DTBP (2.2 eq., 0.44 mmol, 99 µL). SiO_2 used for purification). Isolated as a 1:1 mixture of isomers.

Isolated yield: 50.3 mg, 70%. **^{1}H NMR** (400 MHz, $CDCl_3$) δ 8.02 – 7.91 (m, 1H), 7.78 – 7.69 (m, 2H), 7.68 – 7.59 (m, 1H), 7.25 – 7.18 (m, 3H), 7.16 – 7.07 (m, 2H), 3.40 – 3.30 (m, 1H), 3.14 (dd, *J* = 13.6, 9.4 Hz, 1H), 2.55 – 2.25 (m, 2H), 1.22 – 1.08 (m, 3H). **^{13}C NMR** (101 MHz, $CDCl_3$) δ 165.57, 165.54, 164.56, 164.17, 146.68, 145.87, 144.24, 143.75, 134.78, 134.74, 134.02, 133.92, 132.76, 132.63, 129.53, 129.52, 129.21, 129.00, 128.97, 127.89, 123.96, 122.70, 122.48, 117.57, 115.72, 57.71, 57.64, 38.60, 38.55, 20.21, 19.89, 14.16, 13.94. **HRMS (ESI):** exact mass calculated for $[M+Na]^+$ requires 369.1210 m/z, found 369.1212 m/z. **ATR-FTIR** (cm^{-1}): 3030, 2965, 2932, 2873, 1823, 1765, 1741, 1659, 1613, 1497, 1472, 1456, 1385, 1346, 1294, 1271, 1225, 1180, 1146, 1121, 1093, 1074, 1031, 1013, 987, 911, 836, 775, 731, 700, 649, 621, 608, 585.

5-benzyl-9(/10)-(*tert*-butyl)-2-methylene-[1,3,5]oxadiazepino[4,5-*a*]isoindole-4,7(2*H*,5*H*)-dione (**9k/9k'**)

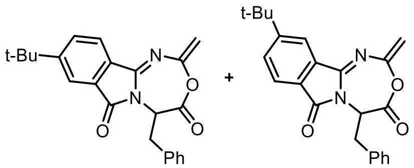

Synthesized following the general procedure **C**. (Reaction time = 0.5 h. 1.1 eq. Tf_2O used (0.22 mmol, 37 µL). Base = DTBP (2.2 eq., 0.44 mmol, 99 µL). SiO_2 used for purification). Isolated as a 1:1 mixture of isomers.

Isolated yield: 47.4 mg, 63%. **^{1}H NMR** (600 MHz, $CDCl_3$) δ 8.01 – 7.86 (m, 1H), 7.80 – 7.75 (m, 1H), 7.71 – 7.67 (m, 1H), 7.25 – 7.22 (m, 3H), 7.20 – 7.14 (m, 2H), 5.48 – 5.38 (m, 1H), 5.02 – 4.93 (m, 2H), 3.45 – 3.38 (m, 1H), 3.25 – 3.14 (m, 1H), 1.42 – 1.35 (m, 9H). **^{13}C NMR** (151 MHz, $CDCl_3$) δ 166.01, 165.53, 163.64, 158.87, 157.71, 150.50, 150.46, 148.49, 148.07, 134.68, 133.98, 131.89, 131.50, 130.44, 129.58, 129.22, 129.04, 127.94, 126.81, 123.90, 122.62, 121.10, 119.84, 97.38, 97.36, 57.71, 57.67, 38.80, 38.74, 31.33, 31.30. **HRMS (ESI):** exact mass calculated for $[M+Na]^+$ requires 397.1523 m/z, found 397.1513 m/z. **ATR-FTIR** (cm^{-1}): 3027, 2965, 1773, 1714, 1666, 1620, 1496, 1455, 1432, 1384, 1259, 1218, 1184, 1106, 1061, 848, 752, 700, 671.
848, 824, 794, 777, 728, 704, 672, 649, 609.

5-benzyl-10-bromo-2-methylene-[1,3,5]oxadiazepino[4,5-*a*]isoindole-4,7(2*H*,5*H*)-dione (**9l'**)

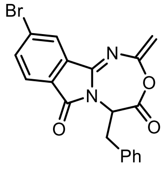

Synthesized following the general procedure **C**. (Reaction time = 0.5 h. 1.1 eq. Tf_2O used (0.22 mmol, 37 µL). Base = DTBP (2.2 eq., 0.44 mmol, 99 µL). SiO_2 used for purification). Isolated as a 5:2 mixture of isomers.

Isolated yield: 51.6 mg, 65%. (Major) **^{1}H NMR** (600 MHz, $CDCl_3$) δ 7.90 – 7.80 (m, 3H), 7.25 – 7.21 (m, 3H), 7.15 – 7.09 (m, 2H), 5.42 (t, *J* = 8.6 Hz, 1H), 5.03 (s, 1H), 4.99 (s, 1H), 3.40 (dd, *J* = 13.6, 7.6 Hz, 1H), 3.19 (dd, *J* = 13.4, 9.9 Hz, 1H). **^{13}C NMR** (151 MHz, $CDCl_3$) δ 164.12, 163.30, 150.15, 146.96, 137.22, 133.66, 133.12, 131.03, 129.20 (2xC), 129.08 (2xC), 128.07, 127.79, 127.28, 124.20, 98.48, 57.85, 38.60. **HRMS (ESI):** exact mass calculated for $[M-C_2+Na]^+$ requires 395.0002 m/z, found 394.9996 m/z. **ATR-FTIR** (cm^{-1}): 3316, 3280, 3261, 3243, 3211, 3143, 3128, 3060, 3028, 2985, 2926, 2855, 1720, 1663, 1606, 1497, 1455, 1422, 1381, 1260, 1216, 1177, 1107, 1075, 1031, 1003, 983, 912, 840, 754, 700, 667, 583.

5-benzyl-9-bromo-2-methylene-[1,3,5]oxadiazepino[4,5-*a*]isoindole-4,7(2*H*,5*H*)-dione (**9l**)

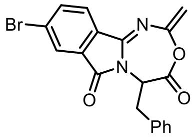

(Minor) ^{1}**H NMR** (600 MHz, $CDCl_3$) δ 8.12 (s, 1H), 7.78 (d, *J* = 8.0 Hz, 1H), 7.59 (d, *J* = 8.0 Hz, 1H), 7.25 – 7.21 (m, 3H), 7.15 – 7.09 (m, 2H), 5.42 (t, *J* = 8.6 Hz, 1H), 5.03 (s, 1H), 4.99 (s, 1H), 3.40 (dd, *J* = 13.6, 7.6 Hz, 1H), 3.19 (dd, *J* = 13.4, 9.9 Hz, 1H). ^{13}**C NMR** (151 MHz, $CDCl_3$) δ 164.65, 163.30, 150.10, 146.52, 136.20, 133.66, 133.12, 131.03, 129.20, 129.08 (2xC), 127.79 (2xC), 126.17, 125.35, 124.20, 98.64, 57.82, 38.60.

5-benzyl-11-methyl-2-methylene-[1,3,5]oxadiazepino[4,5-*a*]isoindole-4,7(2*H*,5*H*)-dione (**9m,9m'**)

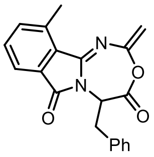

Synthesized following the general procedure **C**. (Reaction time = 0.5 h. 1.1 eq. Tf_2O used (0.22 mmol, 37 µL). Base = DTBP (2.2 eq., 0.44 mmol, 99 µL). SiO_2 used for purification). Isolated as a 9:1 mixture of isomers.

Isolated yield: 23.1 mg, 35%. (Major) ^{1}**H NMR** (600 MHz, $CDCl_3$) δ 7.63 – 7.59 (m, 1H), 7.54 – 7.40 (m, 2H), 7.25 – 7.15 (m, 5H), 5.45 (t, *J* = 8.7 Hz, 1H), 4.98 (d, *J* = 1.3 Hz, 1H), 4.94 (d, *J* = 1.3 Hz, 1H), 3.41 (dd, *J* = 13.7, 8.3 Hz, 1H), 3.21 (dd, *J* = 13.7, 9.1 Hz, 1H), 2.77 (s, 3H). ^{13}C NMR (101 MHz, $CDCl_3$) δ 165.68, 163.75, 150.26, 148.88, 137.96, 136.81, 134.05, 132.46, 130.92, 130.03, 129.22, 129.04 (2xC), 127.94 (2xC), 121.87, 97.57, 57.64, 38.68, 19.04. **HRMS (ESI):** exact mass calculated for $[M+Na]^+$ requires 355.1053 m/z, found 355.1050 m/z. **ATR-FTIR** (cm^{-1}): 3088, 3062, 3030, 2955, 2926, 2349, 2330, 2322, 1769, 1742, 1651, 1620, 1555, 1537, 1511, 1487, 1455, 1423, 1384, 1355, 1260, 1217, 1162, 1113, 1077, 1031, 999, 978, 943, 932, 853, 812, 772, 729, 700, 671, 665, 654, 623, 614, 605, 586.

5-benzyl-8-methyl-2-methylene-[1,3,5]oxadiazepino[4,5-*a*]isoindole-4,7(2*H*,5*H*)-dione (**9m'**)

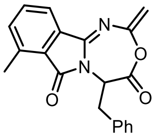

(Minor) ^{1}**H NMR** (600 MHz, $CDCl_3$) δ 7.79 (d, *J* = 7.5 Hz, 1H), 7.58 (t, *J* = 7.7 Hz, 1H), 7.41 (d, *J* = 7.6 Hz, 1H), 7.25 – 7.15 (m, 5H), 5.42 (t, *J* = 8.7 Hz, 1H), 4.98 (d, *J* = 1.3 Hz, 1H), 4.94 (d, *J* = 1.3 Hz, 1H), 3.40 (dd, *J* = 13.7, 8.3 Hz, 1H), 3.19 (dd, *J* = 13.7, 9.1 Hz, 1H), 2.58 (s, 3H). ^{13}**C NMR** (101 MHz, $CDCl_3$) δ 165.68, 163.71, 150.26, 148.88, 138.65, 135.27, 134.05, 133.64, 130.92, 130.03, 129.25, 129.04 (2xC), 127.90 (2xC), 120.44, 97.31, 57.52, 38.78, 17.55.

V. Spectra

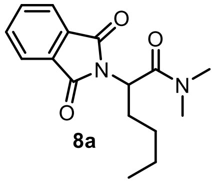

8a

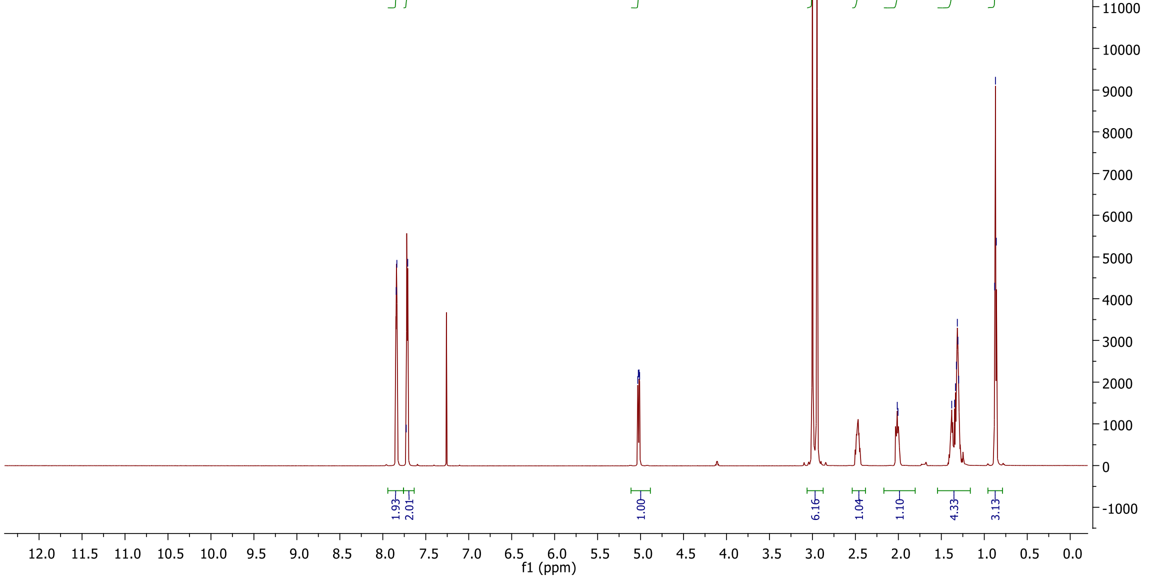
7.84
7.84
7.73
7.71
5.03
5.03
5.02
5.01
3.00
2.95
2.01
2.00
1.32
1.31
1.31
1.30
0.88
0.87
0.86
1.93
2.01
1.00
6.16
1.04
1.10
4.33
3.13
f1 (ppm)

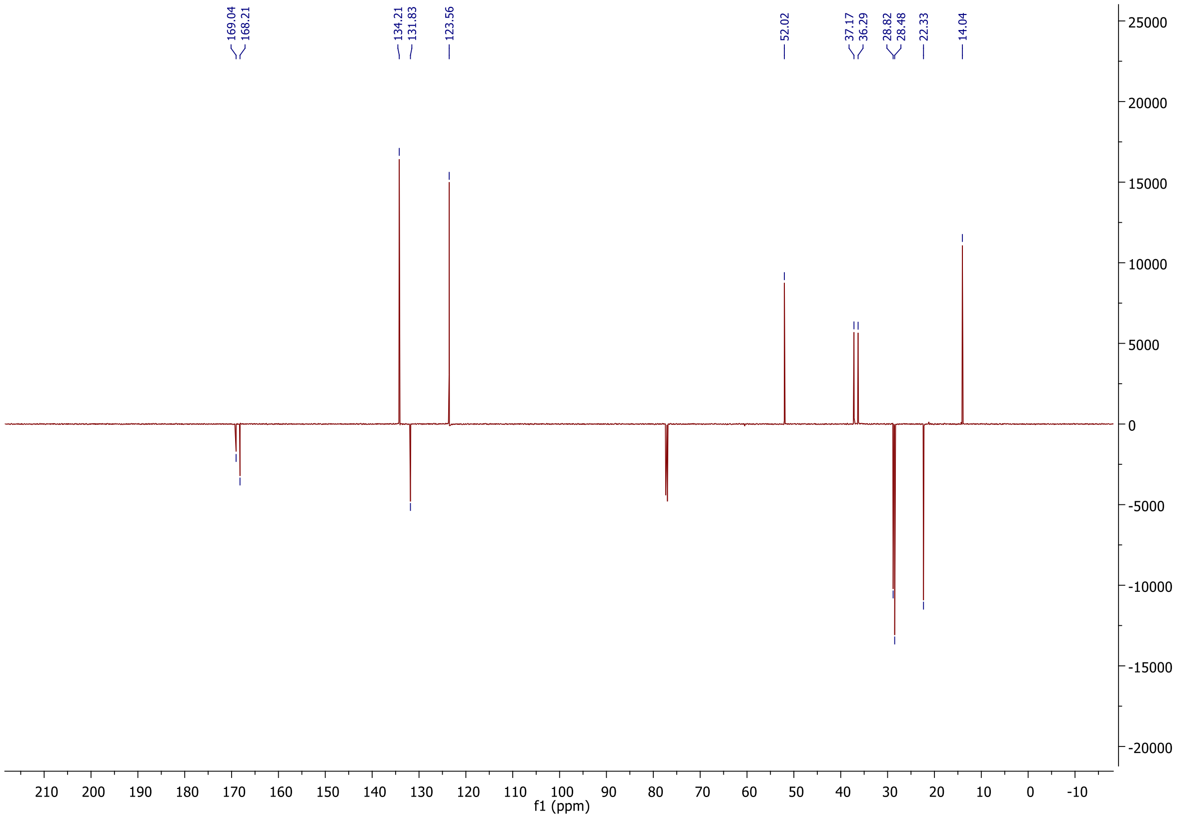
169.04
168.21
134.21
131.83
123.56
52.02
37.17
36.29
28.82
28.48
22.33
14.04
f1 (ppm)

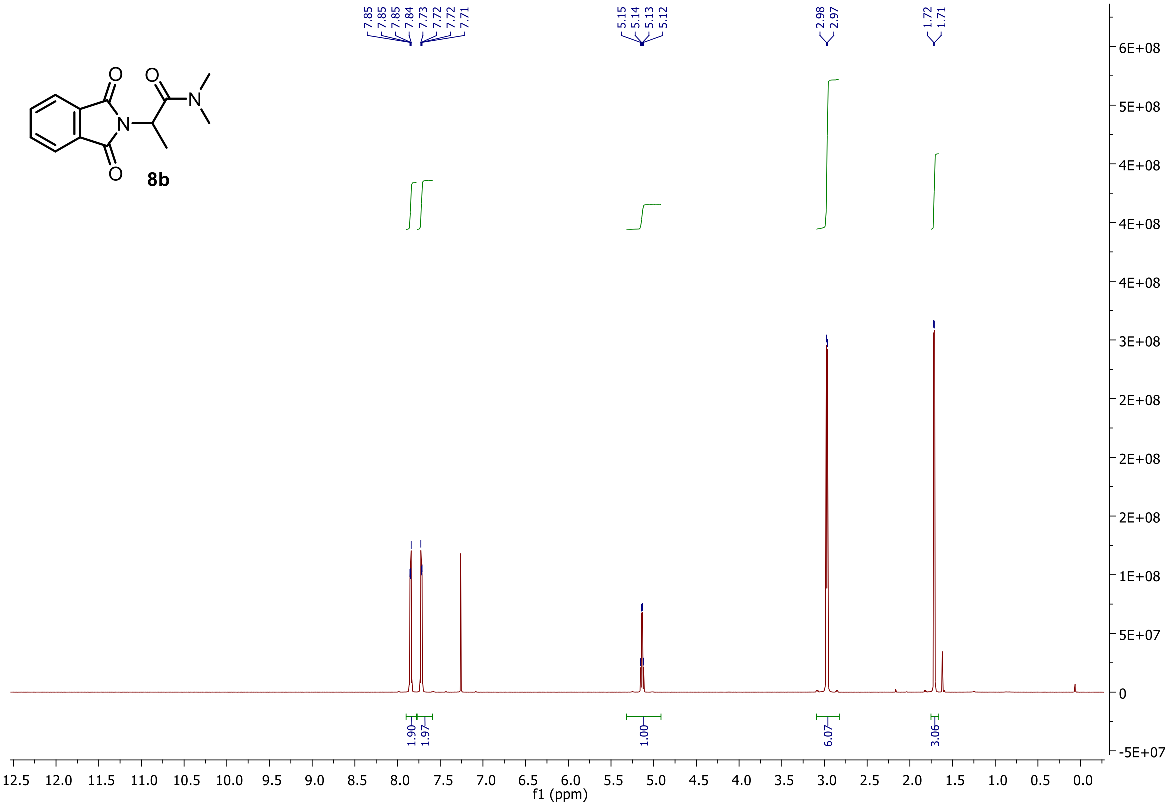
O
O
N
N
O
7.85
7.85
7.85
7.84
7.73
7.72
7.72
7.71
5.15
5.14
5.13
5.12
2.98
2.97
1.72
1.71
1.90
1.97
1.00
6.07
3.06
f1 (ppm)

8b

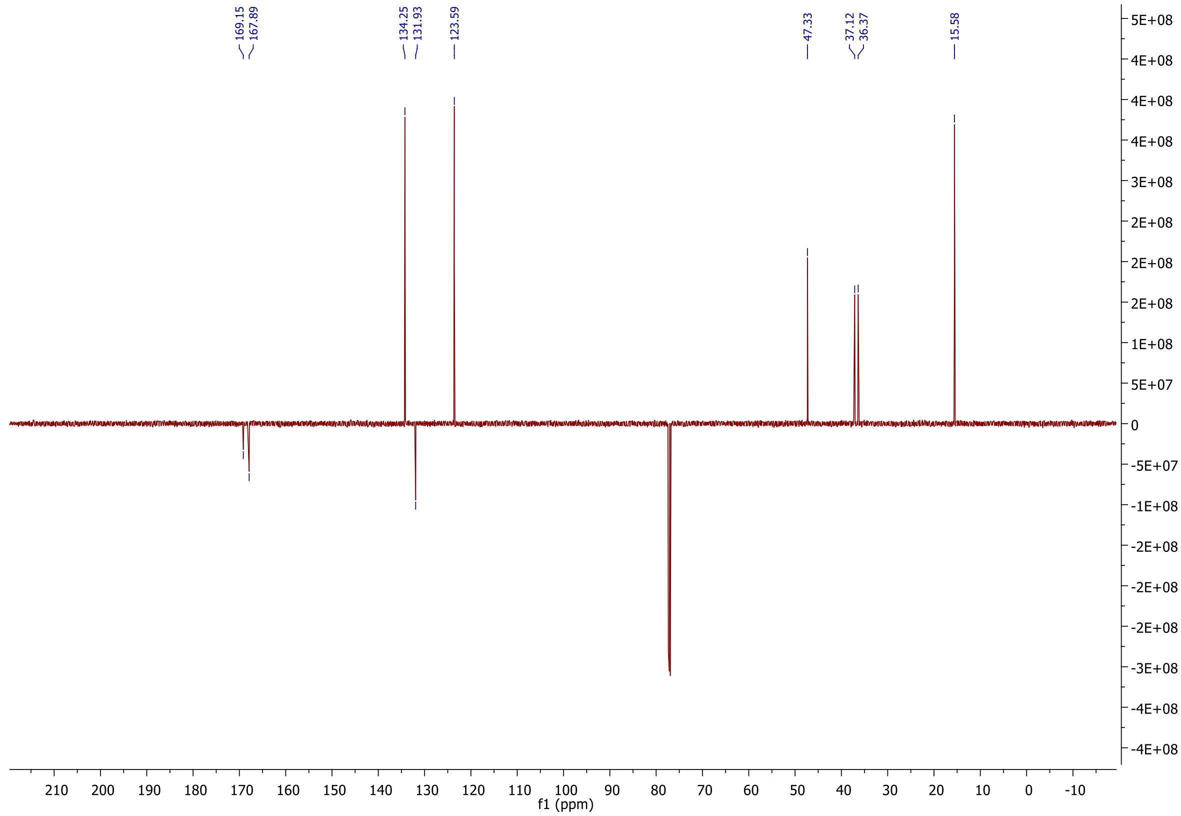
169.15
167.89
134.25
131.93
123.59
47.33
37.12
36.37
15.58
f1 (ppm)

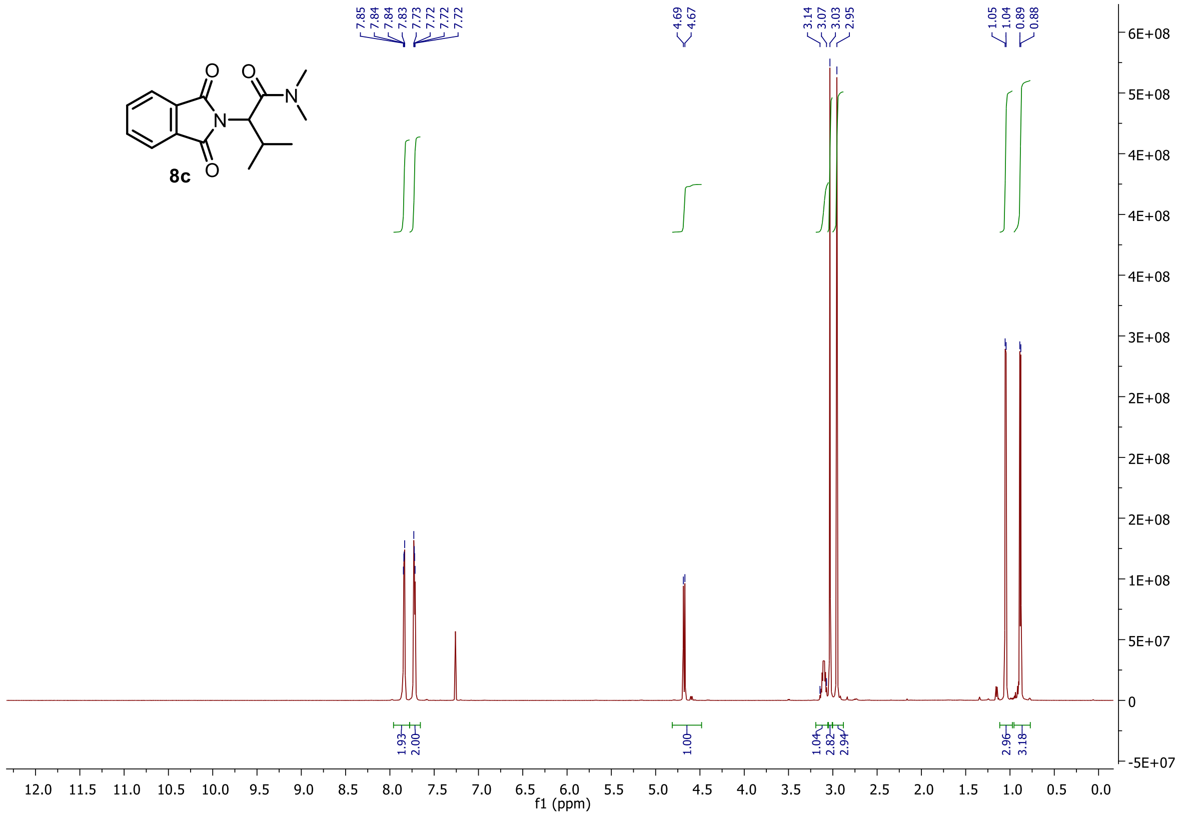
8c
7.85
7.84
7.84
7.83
7.73
7.72
7.72
7.72
4.69
4.67
3.14
3.07
3.03
2.95
1.05
1.04
0.89
0.88
1.93
2.00
1.00
1.04
2.82
2.94
2.96
3.18
f1 (ppm)

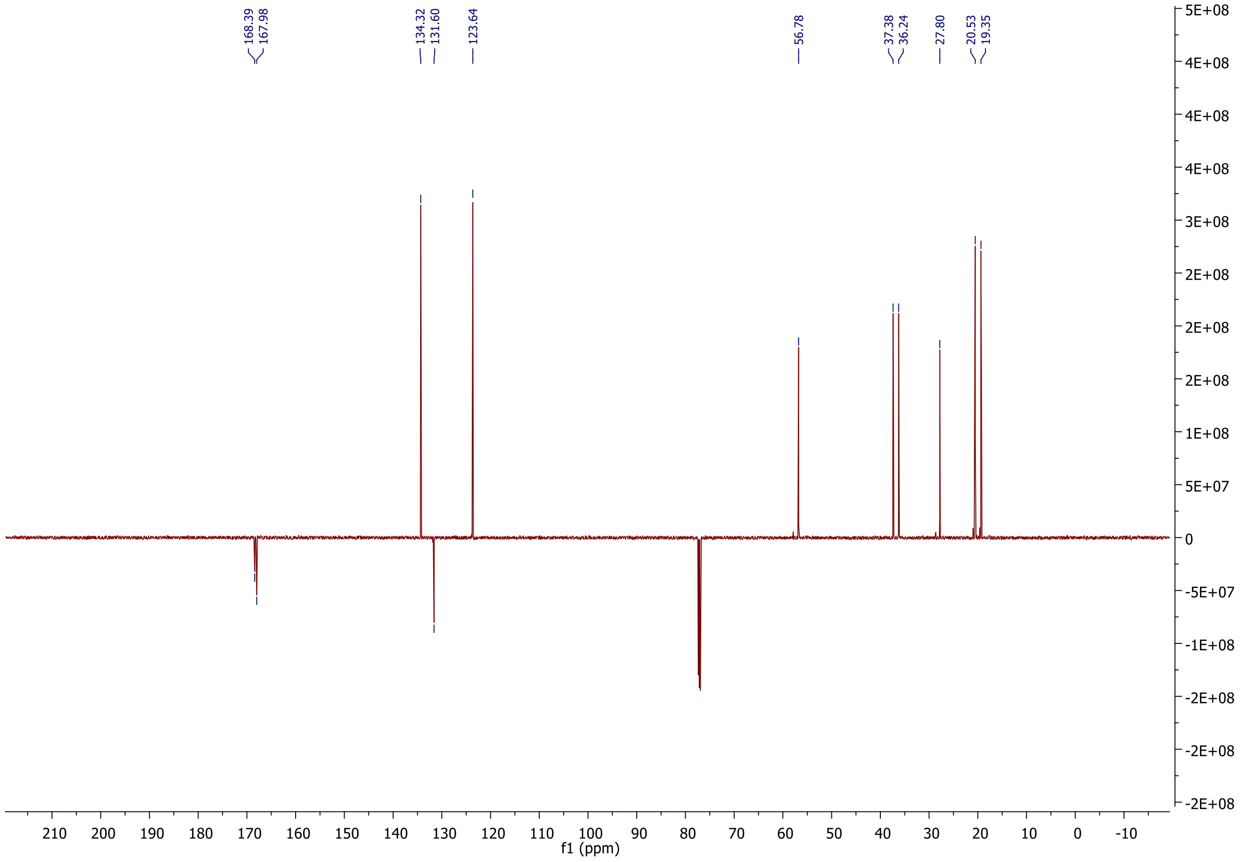
168.39
167.98
134.32
131.60
123.64
56.78
37.38
36.24
27.80
20.53
19.35
f1 (ppm)

8d

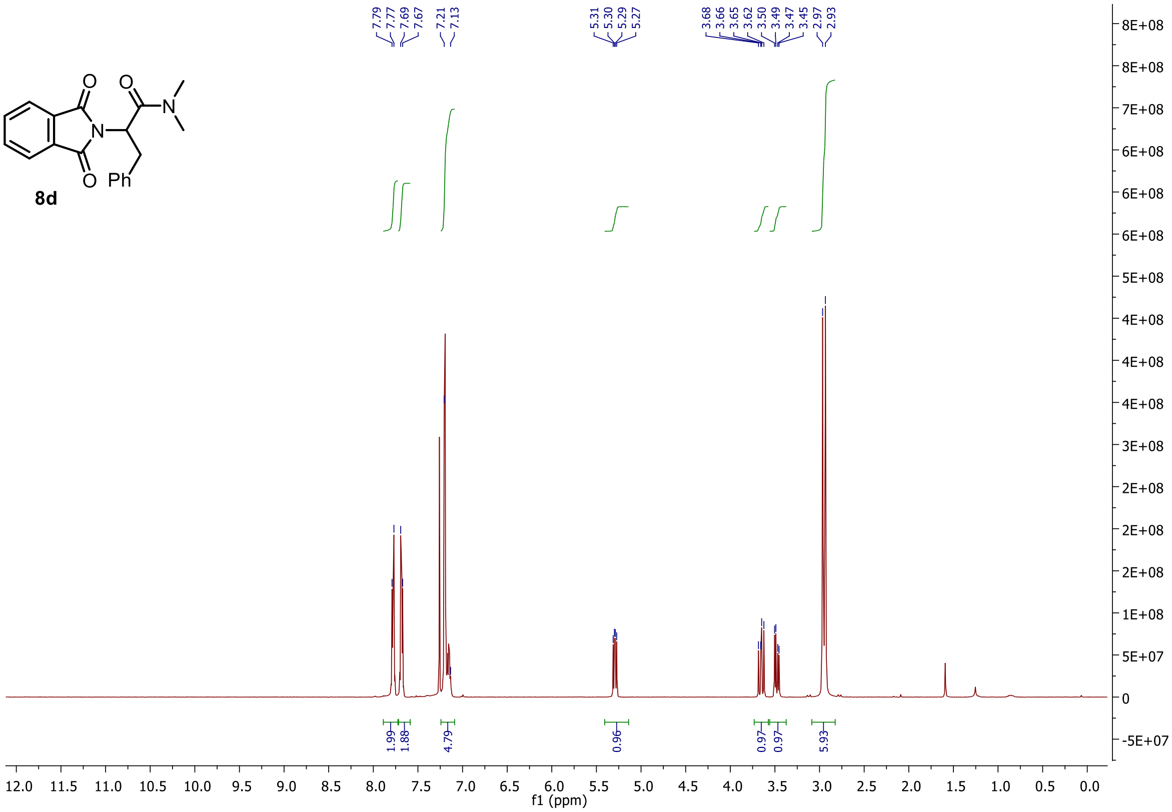
O
O
N
O
N
Ph
7.79
7.77
7.69
7.67
7.21
7.13
5.31
5.30
5.29
5.27
3.68
3.66
3.65
3.62
3.50
3.49
3.47
3.45
2.97
2.93
1.99
1.88
4.79
0.96
0.97
0.97
5.93
f1 (ppm)

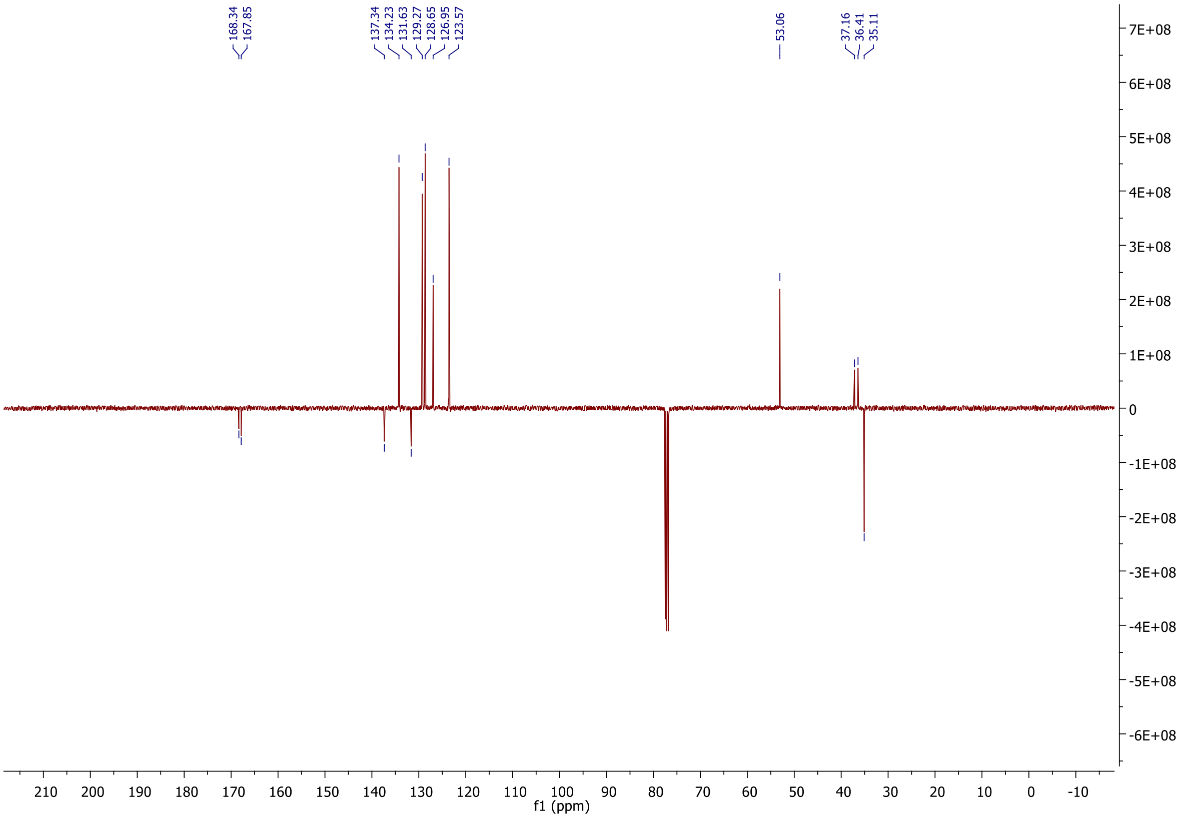
168.34
167.85
137.34
134.23
131.63
129.27
128.65
126.95
123.57
53.06
37.16
36.41
35.11
f1 (ppm)

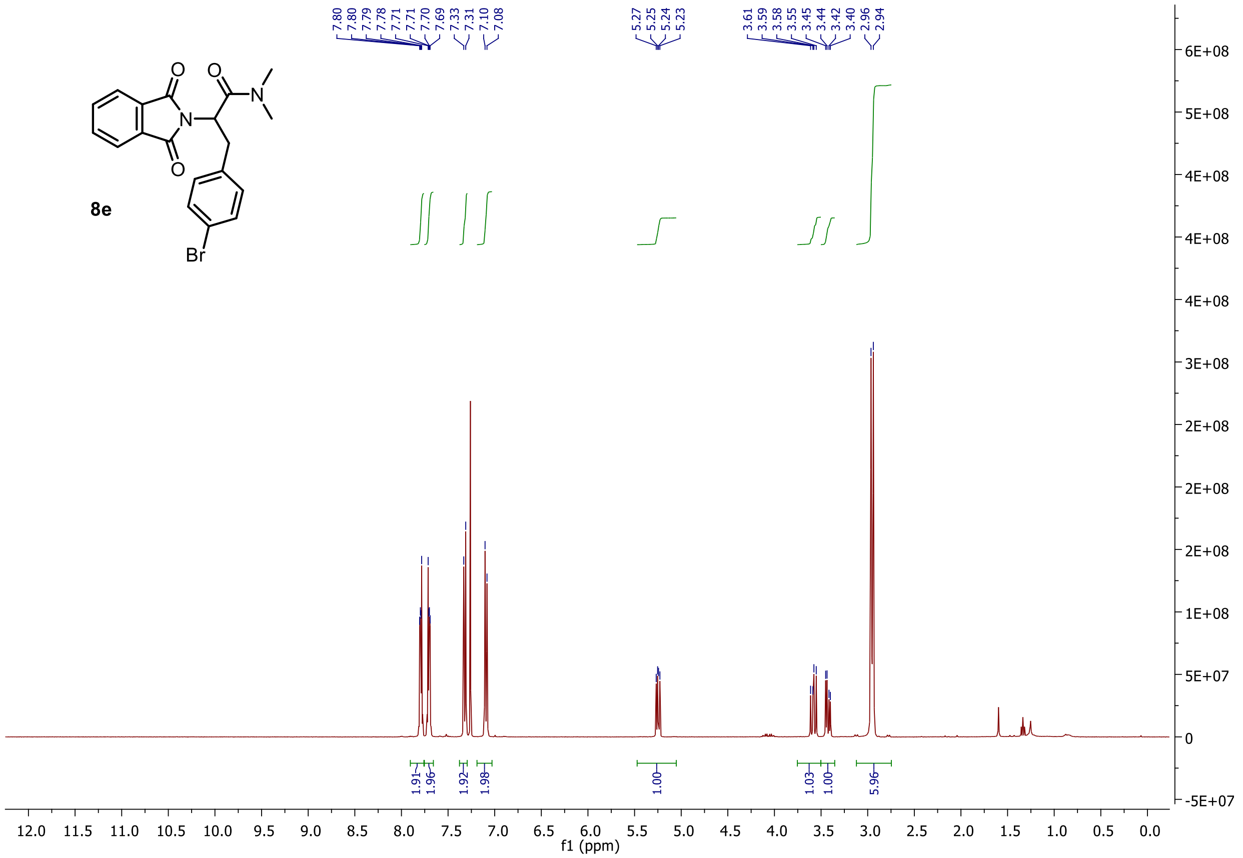
O
O
N
N
O
Br
8e
7.80
7.80
7.79
7.78
7.71
7.71
7.70
7.69
7.33
7.31
7.10
7.08
5.27
5.25
5.24
5.23
3.61
3.59
3.58
3.55
3.45
3.44
3.42
3.40
2.96
2.94
1.91
1.96
1.92
1.98
1.00
1.03
1.00
5.96
f1 (ppm)

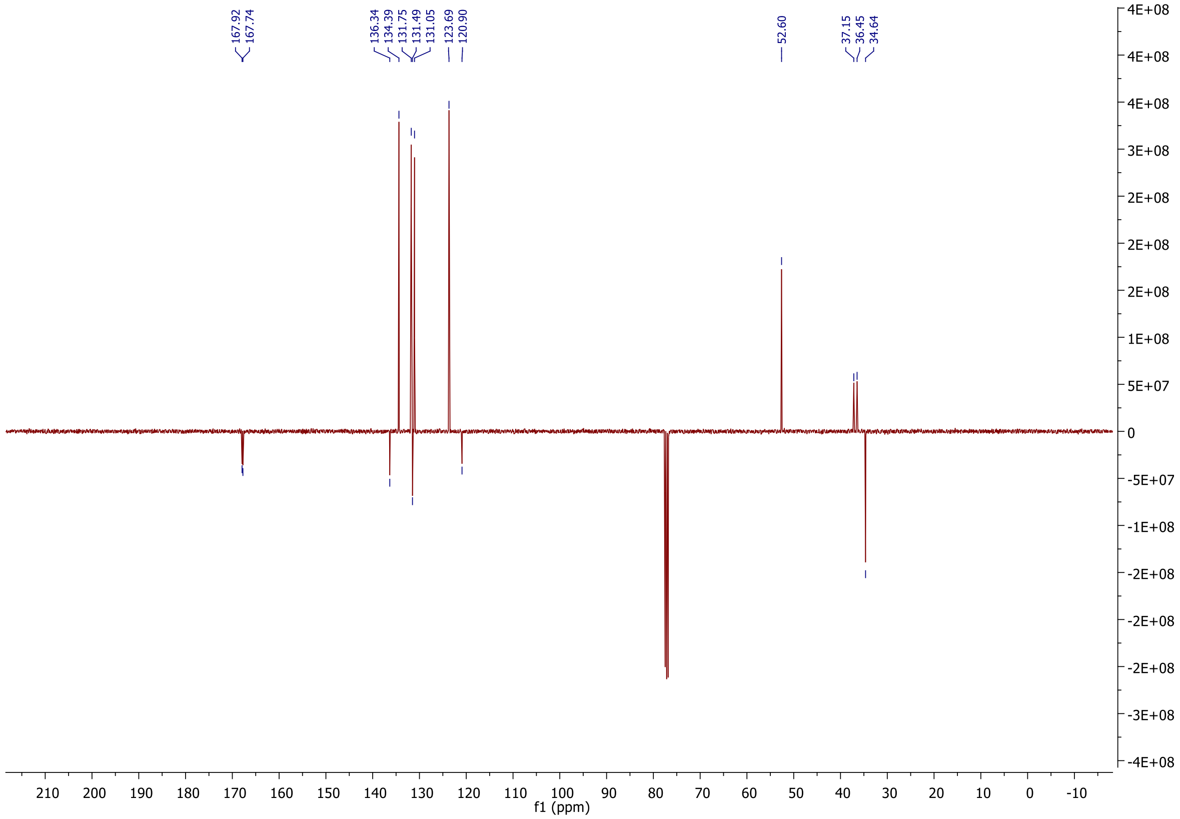
167.92
167.74
136.34
134.39
131.75
131.49
131.05
123.69
120.90
52.60
37.15
36.45
34.64
f1 (ppm)

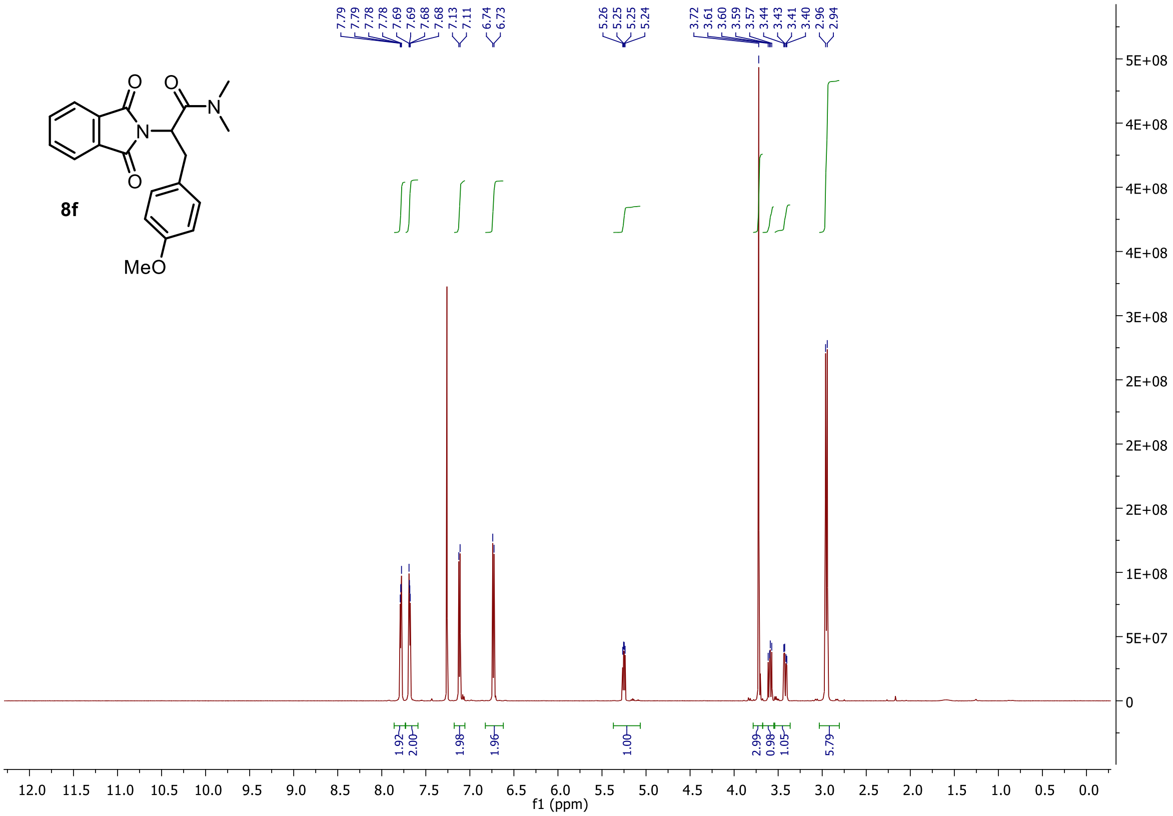
8f
MeO
7.79
7.79
7.78
7.78
7.69
7.69
7.68
7.68
7.13
7.11
6.74
6.73
5.26
5.25
5.25
5.24
3.72
3.61
3.60
3.59
3.57
3.44
3.43
3.41
3.40
2.96
2.94
1.92
2.00
1.98
1.96
1.00
2.99
0.98
1.05
5.79
f1 (ppm)

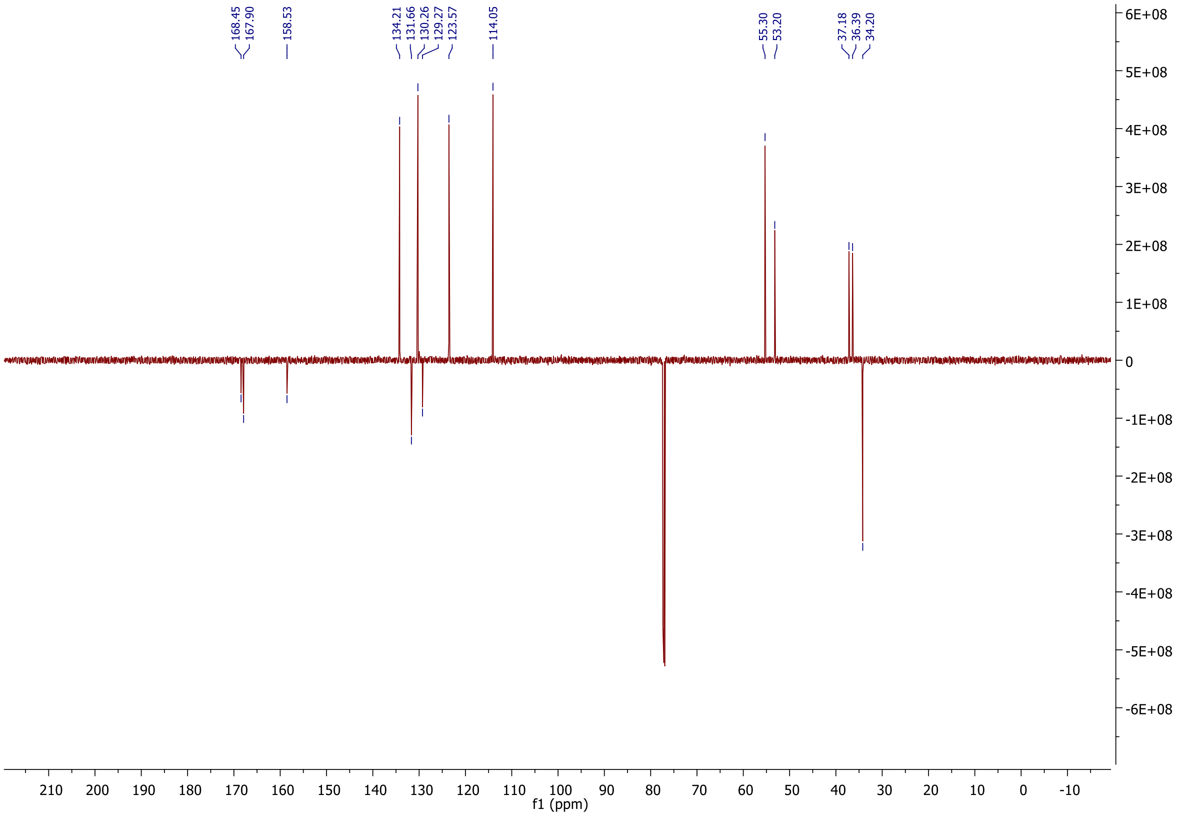
168.45
167.90
158.53
134.21
131.66
130.26
129.27
123.57
114.05
55.30
53.20
37.18
36.39
34.20
f1 (ppm)

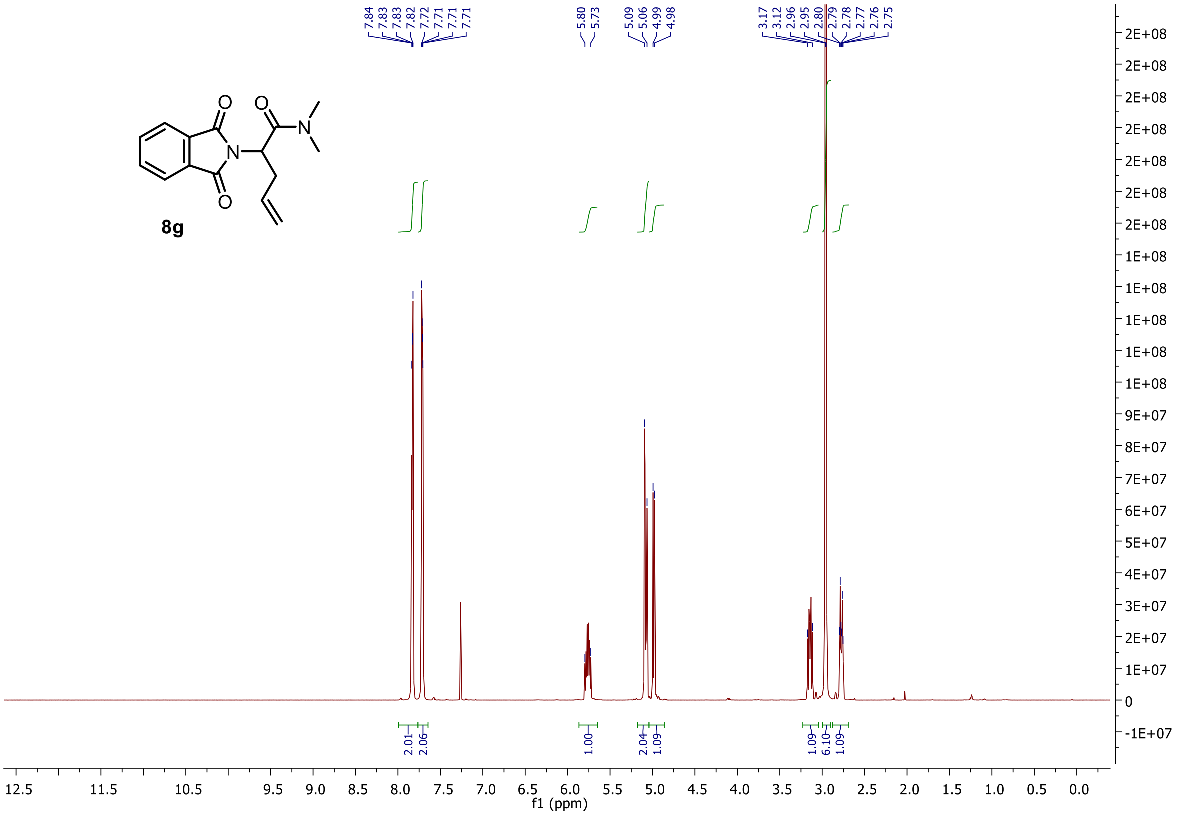
7.84
7.83
7.83
7.82
7.72
7.71
7.71
7.71
5.80
5.73
5.09
5.06
4.99
4.98
3.17
3.12
2.96
2.95
2.80
2.79
2.78
2.77
2.76
2.75
8g
2.01
2.06
1.00
2.04
1.09
1.09
6.10
1.09
f1 (ppm)

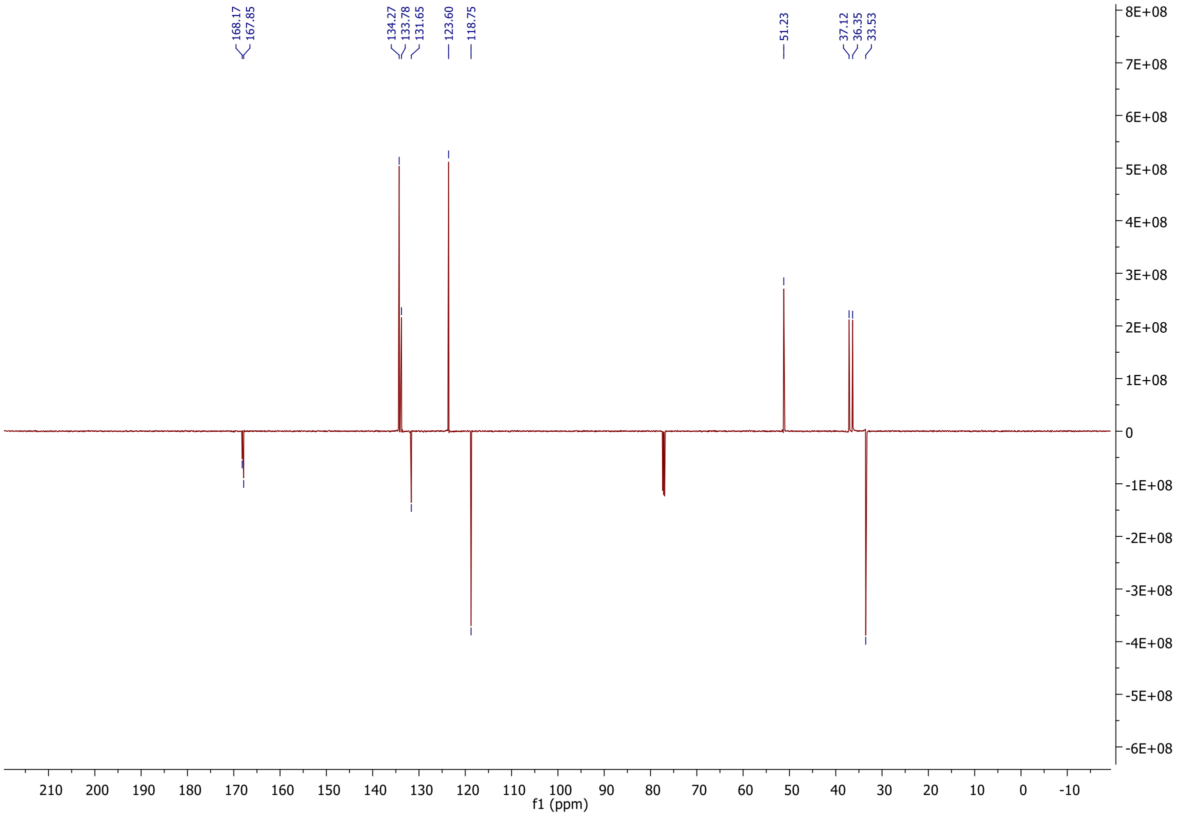
168.17
167.85
134.27
133.78
131.65
123.60
118.75
51.23
37.12
36.35
33.53
f1 (ppm)

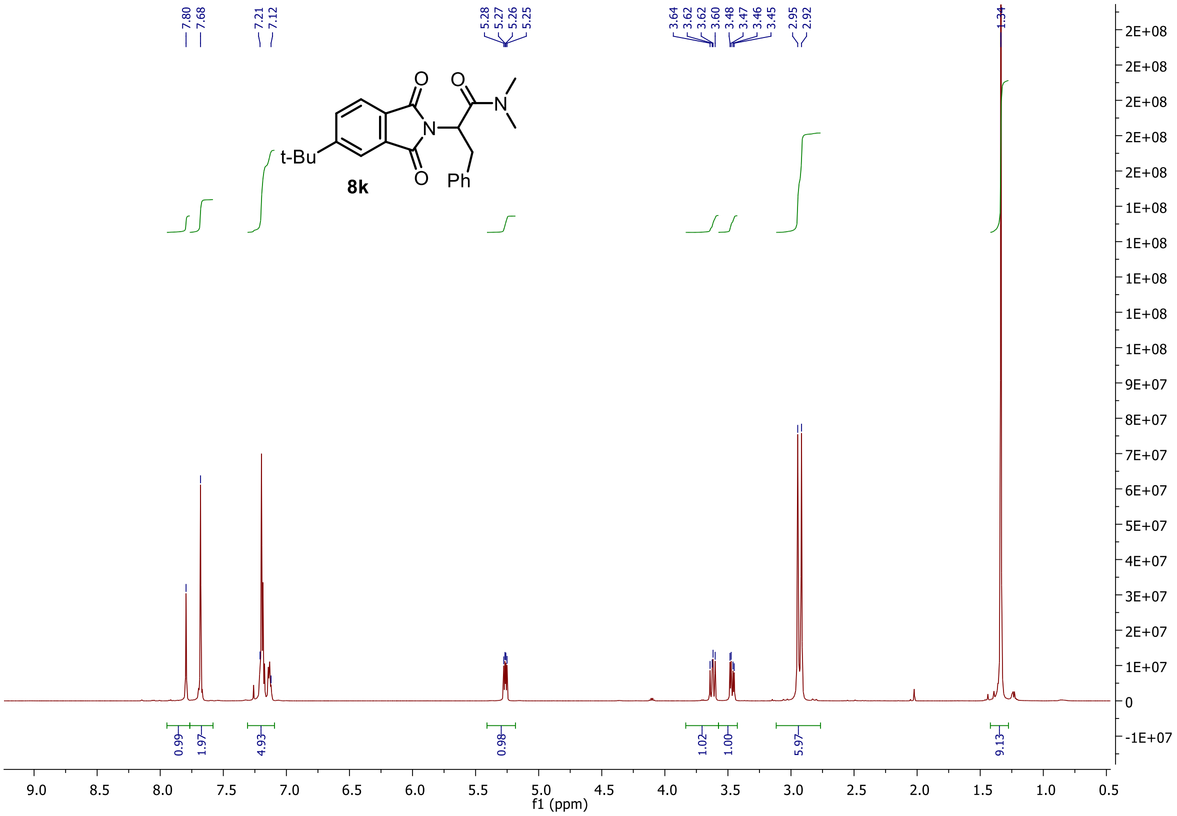
7.80
7.68
7.21
7.12
5.28
5.27
5.26
5.25
3.64
3.62
3.62
3.60
3.48
3.47
3.46
3.45
2.95
2.92
1.34
t-Bu
8k
Ph
0.99
1.97
4.93
0.98
1.02
1.00
5.97
9.13
f1 (ppm)

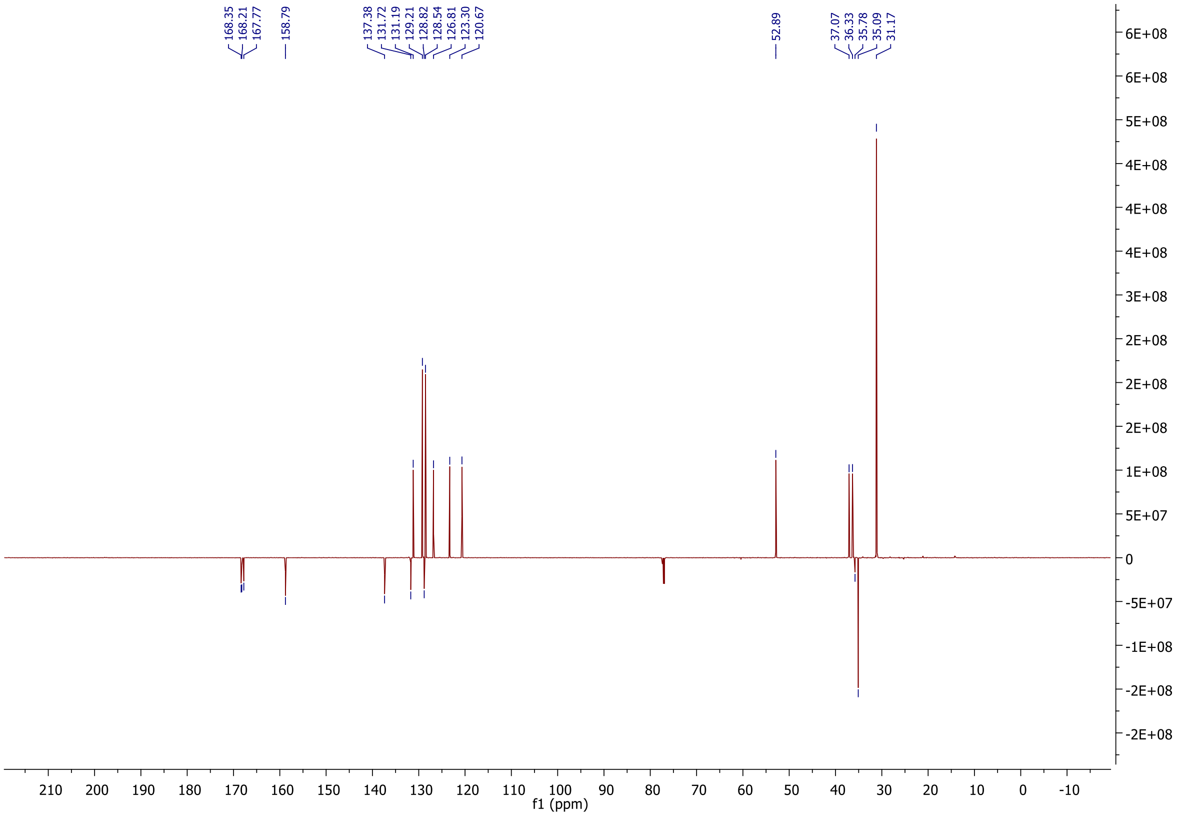
168.35
168.21
167.77
158.79
137.38
131.72
131.19
129.21
128.82
128.54
126.81
123.30
120.67
52.89
37.07
36.33
35.78
35.09
31.17
f1 (ppm)

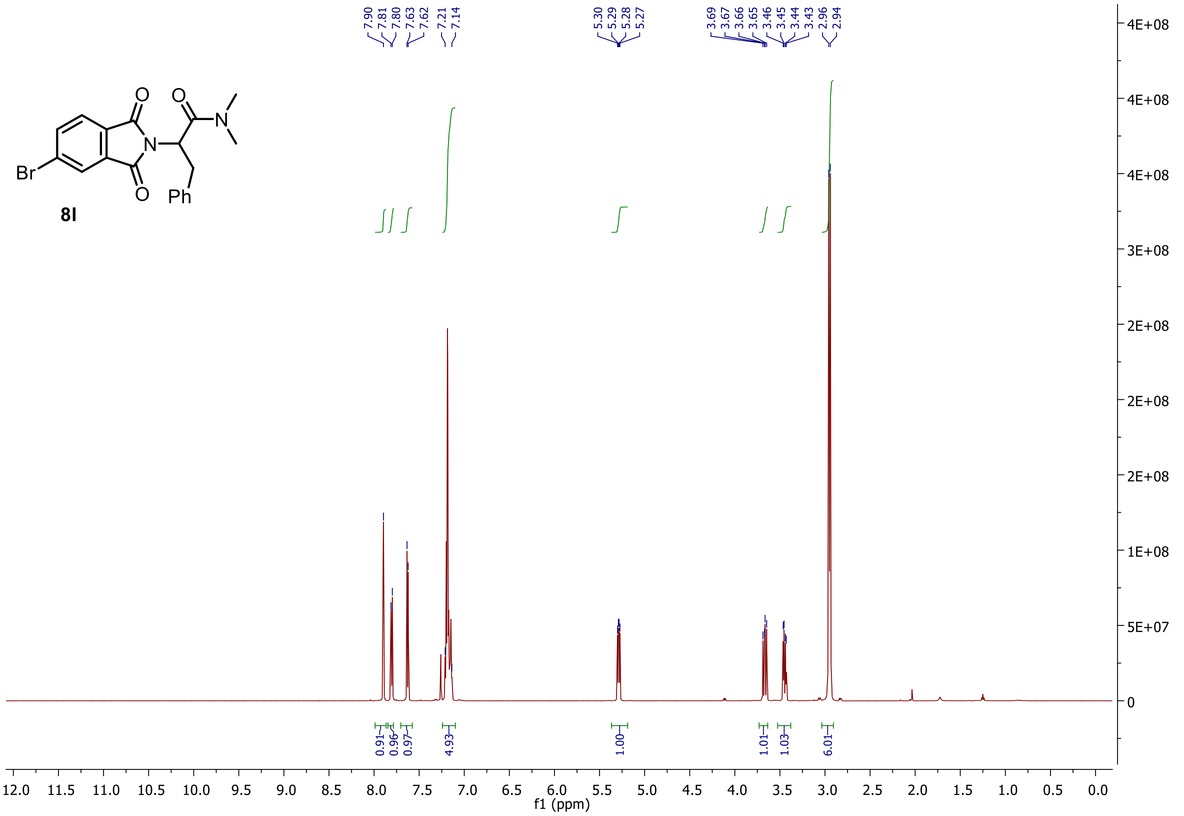
7.90
7.81
7.80
7.63
7.62
7.21
7.14
5.30
5.29
5.28
5.27
3.69
3.67
3.66
3.65
3.46
3.45
3.44
3.43
2.96
2.94
Br
O
O
N
O
N
Ph
8l
0.91
0.96
0.97
4.93
1.00
1.01
1.03
6.01
f1 (ppm)

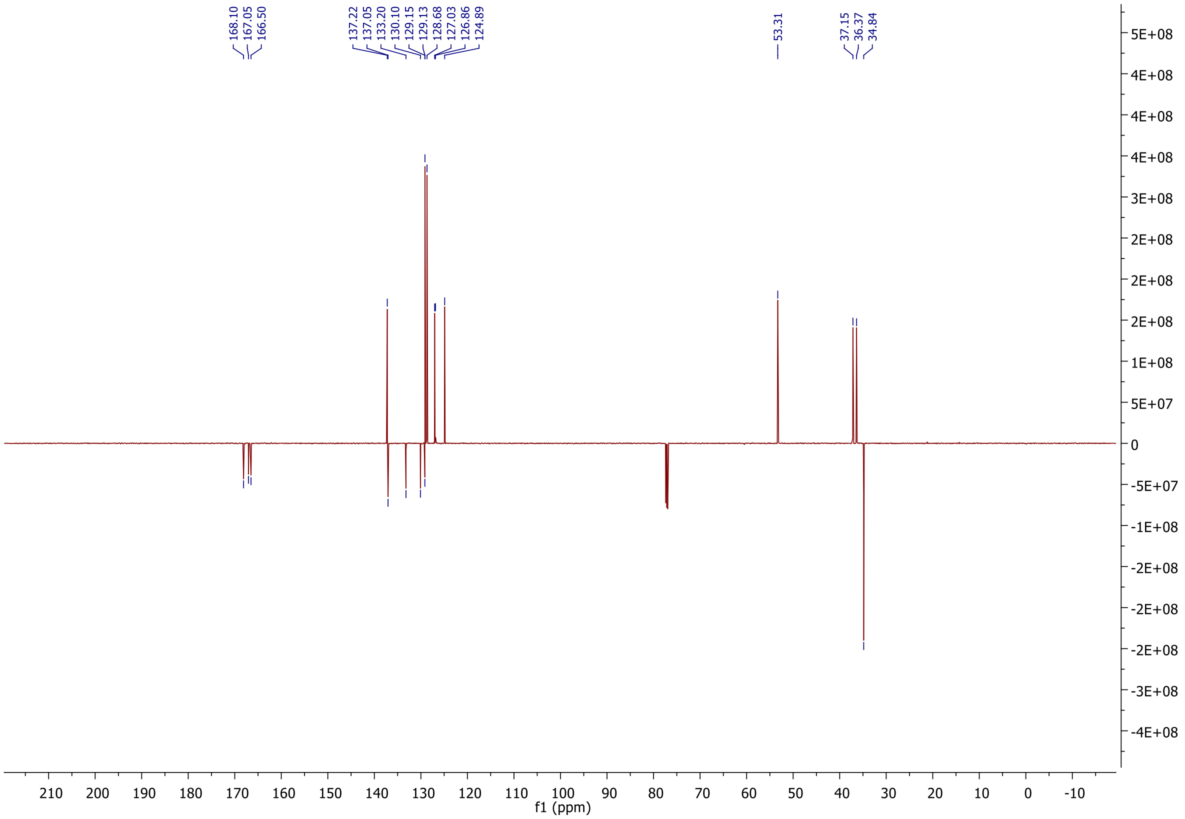
168.10
167.05
166.50
137.22
137.05
133.20
130.10
129.15
129.13
128.68
127.03
126.86
124.89
53.31
37.15
36.37
34.84
f1 (ppm)

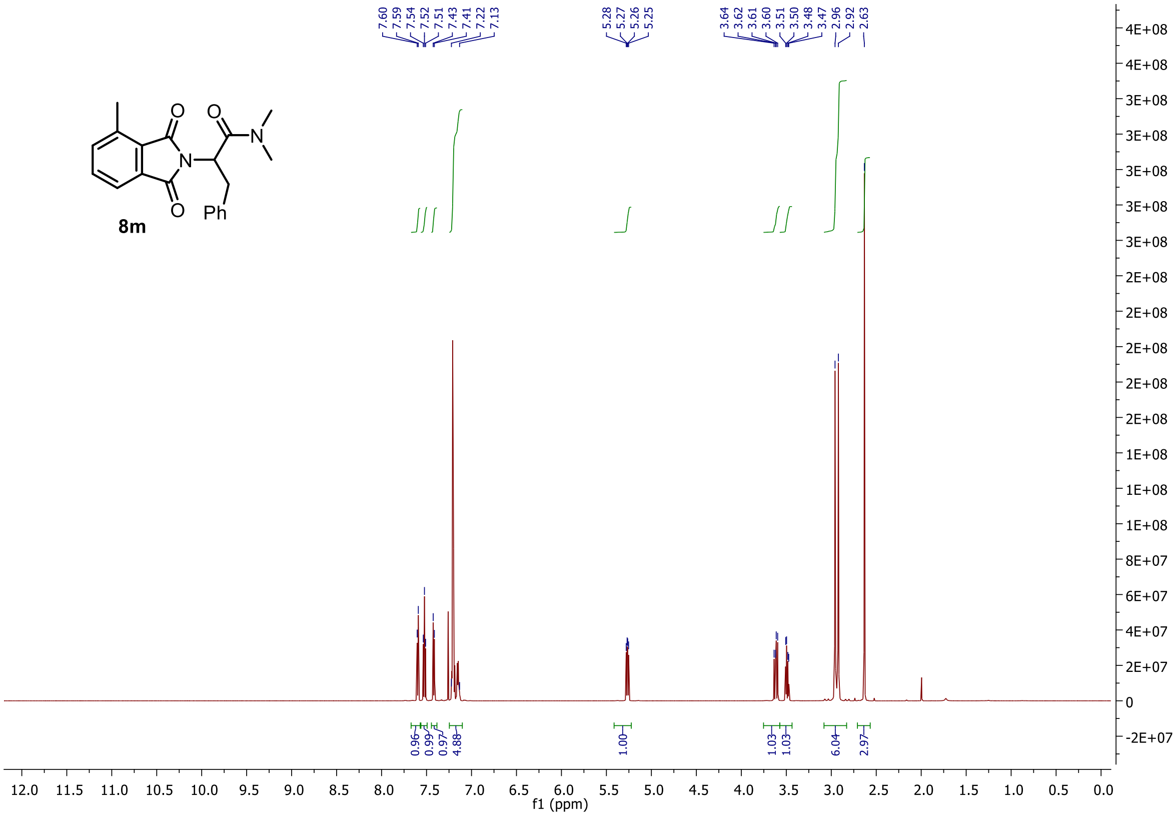
8m
Ph
7.60 7.59 7.54 7.52 7.51 7.43 7.41 7.22 7.13
5.28 5.27 5.26 5.25
3.64 3.62 3.61 3.60 3.51 3.50 3.48 3.47 2.96 2.92 2.63
0.96 0.99 0.97 4.88 1.00 1.03 1.03 6.04 2.97
f1 (ppm)

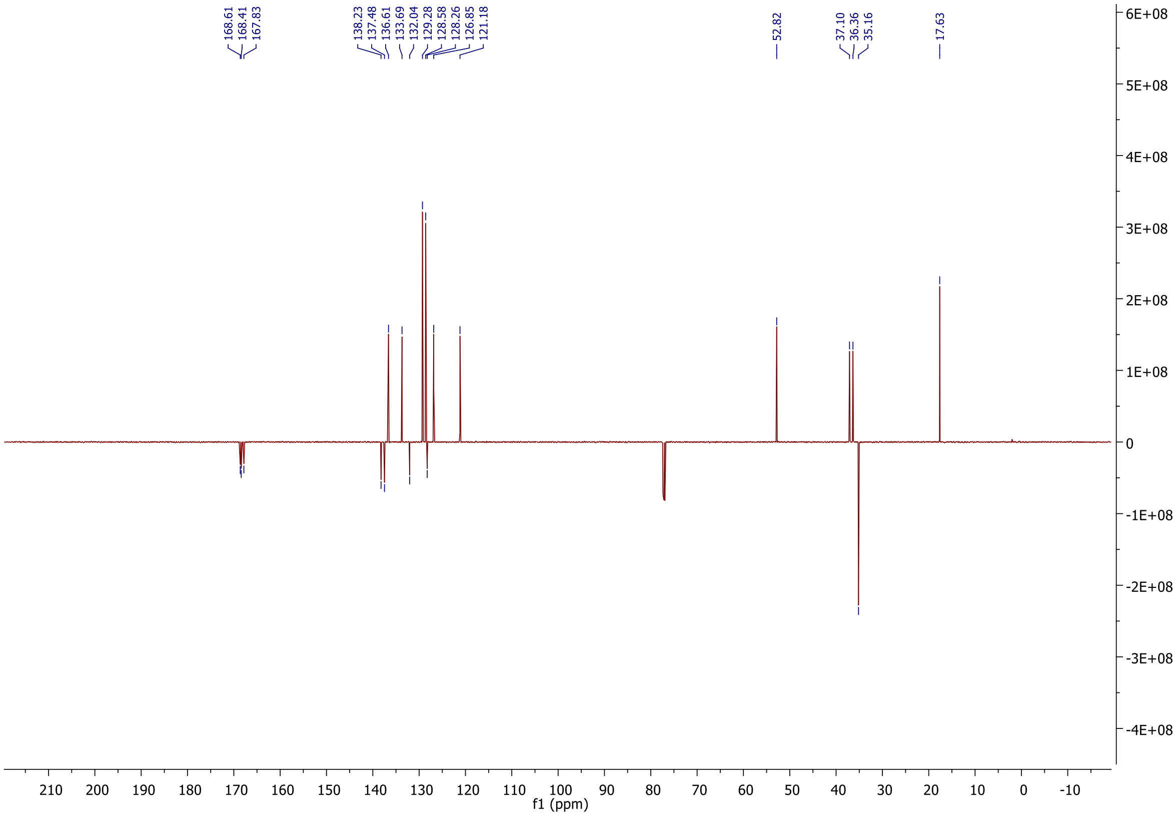
168.61 168.41 167.83
138.23 137.48 136.61 133.69 132.04 129.28 128.58 128.26 126.85 121.18
52.82
37.10 36.36 35.16
17.63
f1 (ppm)

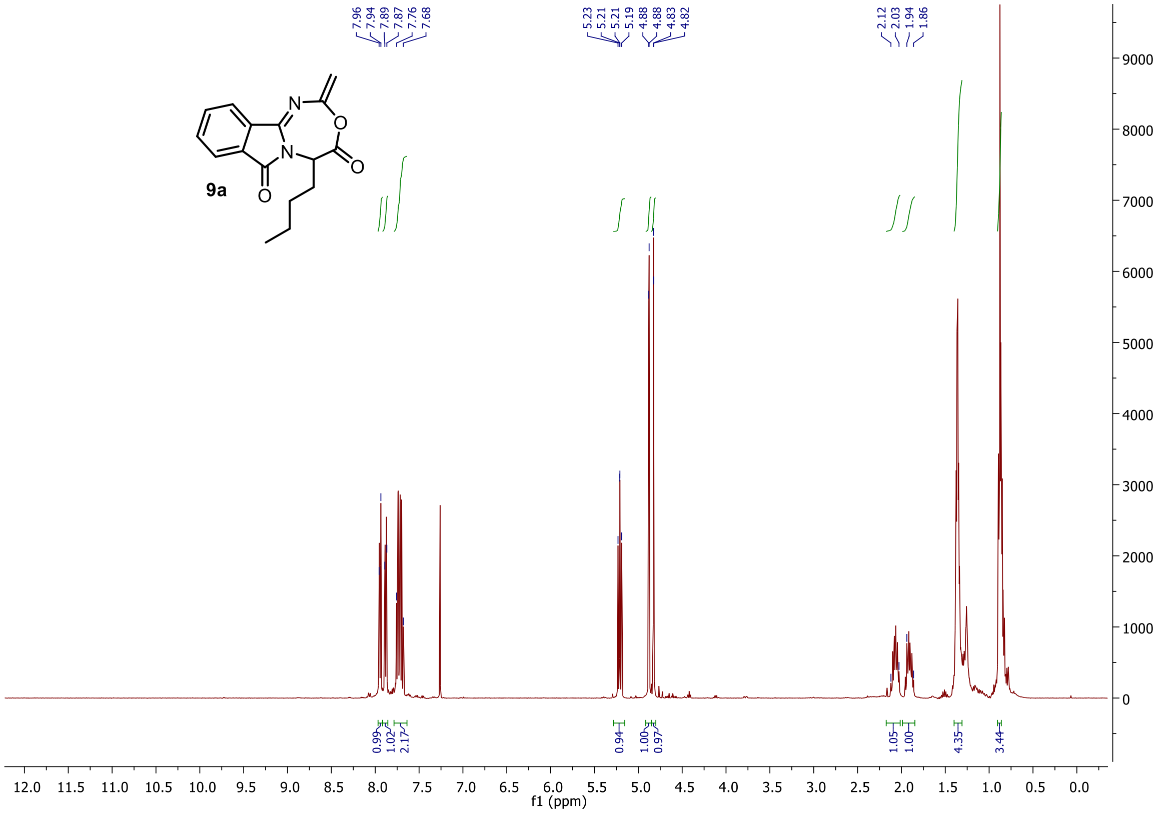
7.96
7.94
7.89
7.87
7.76
7.68
5.23
5.21
5.21
5.19
4.88
4.88
4.83
4.82
2.12
2.03
1.94
1.86
9a
0.99
1.02
2.17
0.94
1.00
0.97
1.05
1.00
4.35
3.44
f1 (ppm)

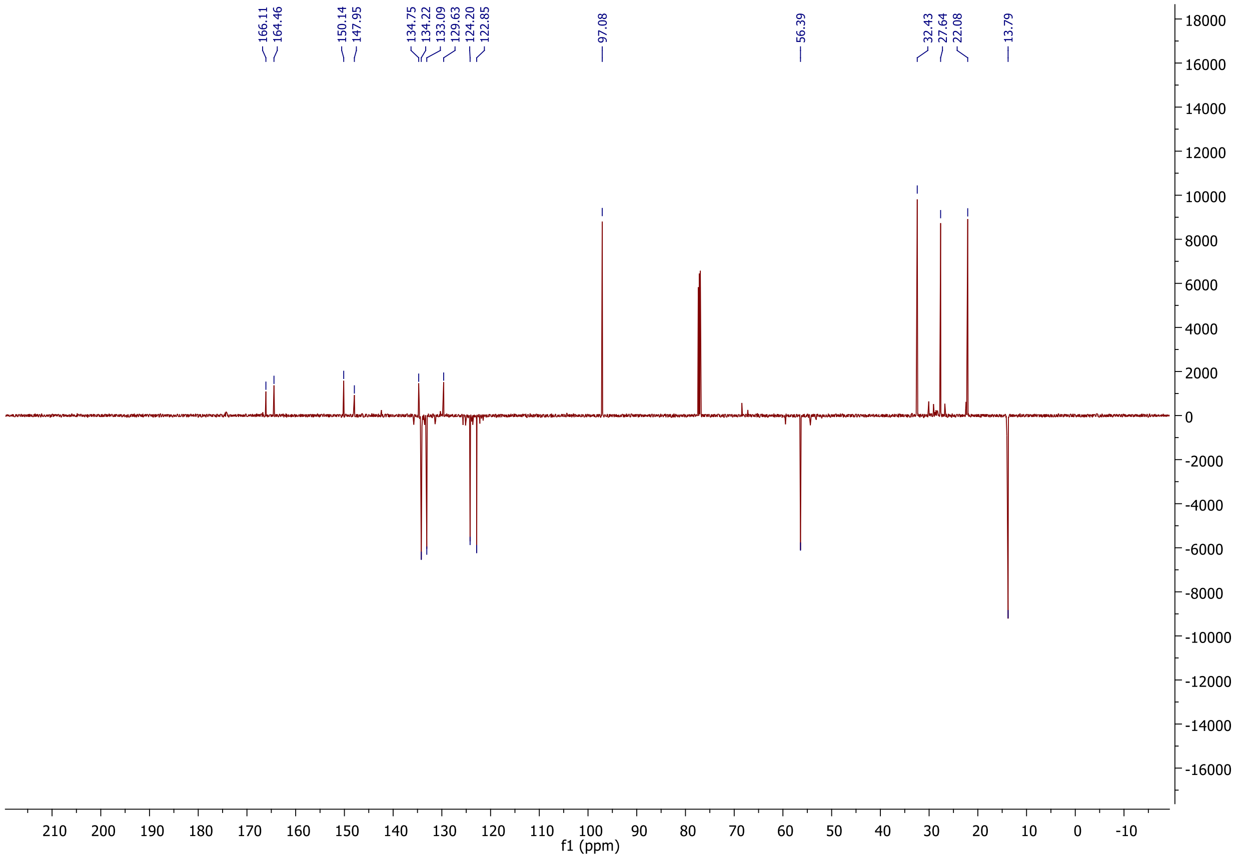
166.11
164.46
150.14
147.95
134.75
134.22
133.09
129.63
124.20
122.85
97.08
56.39
32.43
27.64
22.08
13.79
f1 (ppm)

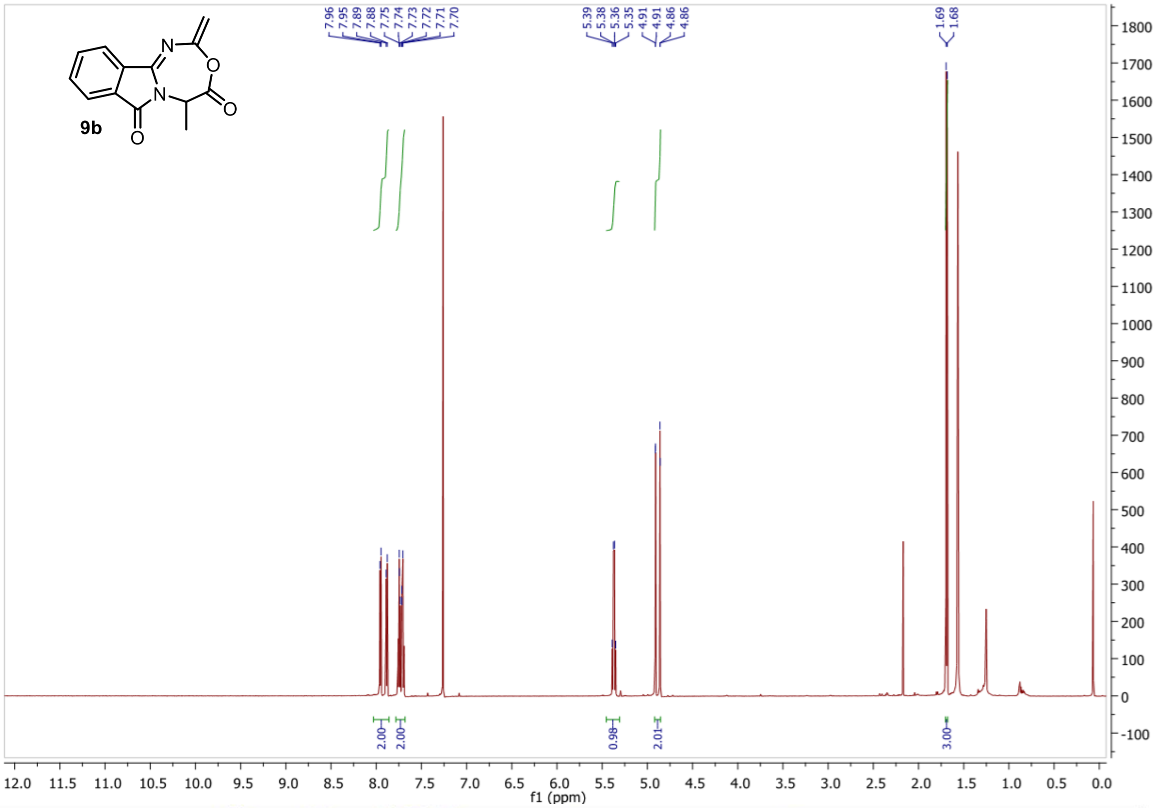
9b
N
O
O
N
O
7.96
7.95
7.89
7.88
7.75
7.74
7.73
7.72
7.71
7.70
5.39
5.38
5.36
5.35
4.91
4.91
4.86
4.86
1.69
1.68
2.00
2.00
0.98
2.01
3.00
f1 (ppm)

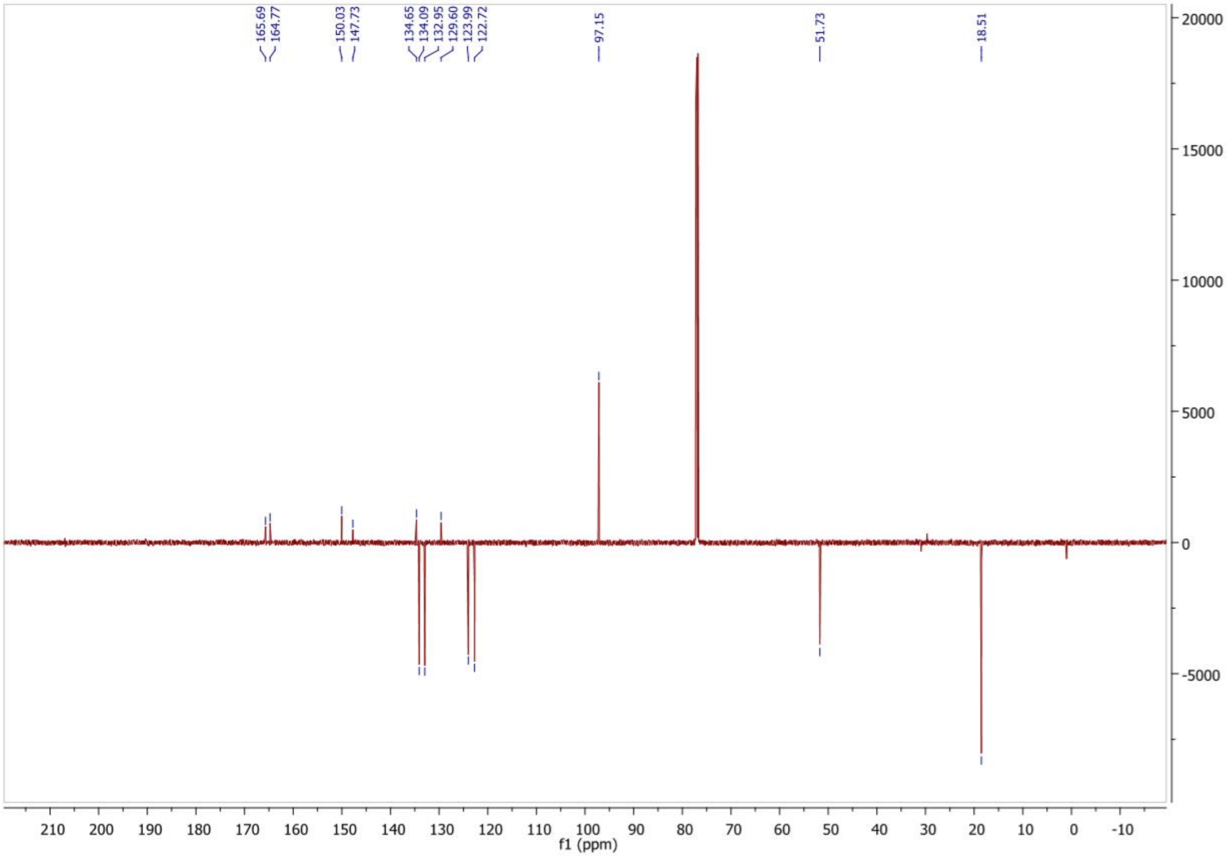
165.69
164.77
150.03
147.73
134.65
134.09
132.95
129.60
123.99
122.72
97.15
51.73
18.51
f1 (ppm)

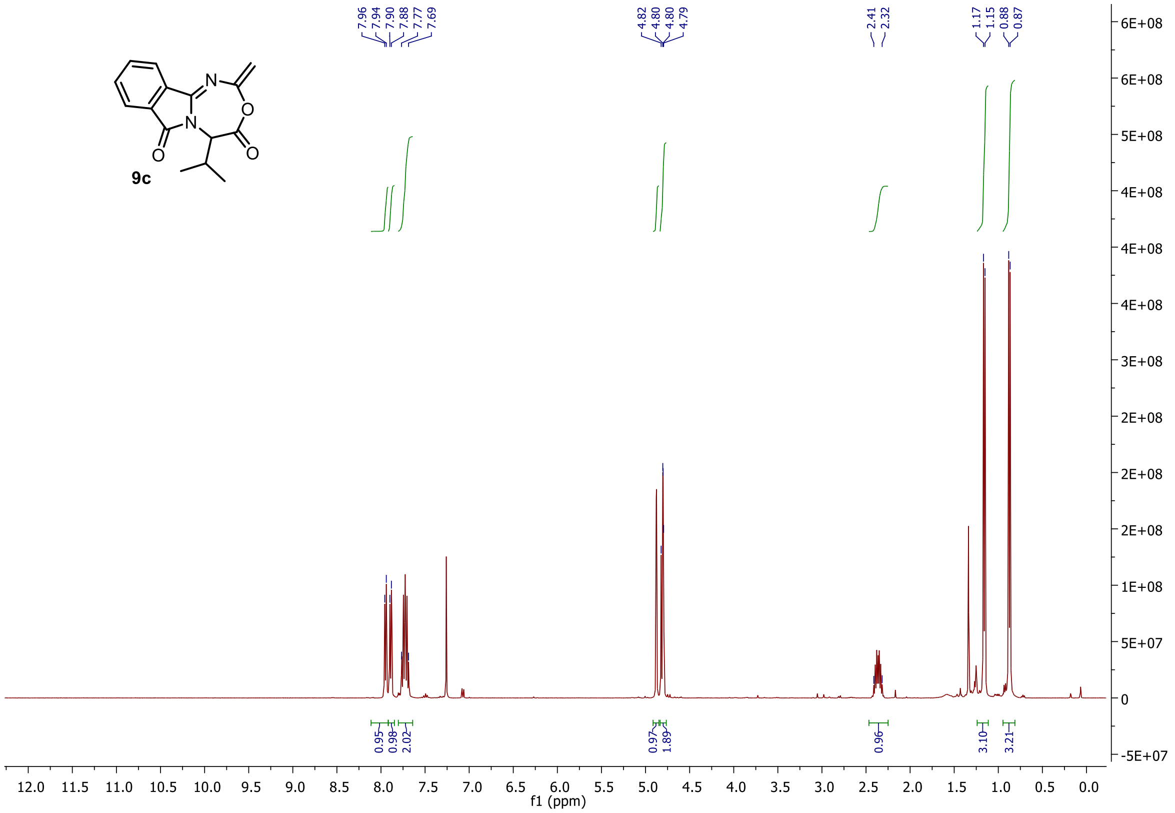
N
O
N
O
O
9c
7.96
7.94
7.90
7.88
7.77
7.69
4.82
4.80
4.80
4.79
2.41
2.32
1.17
1.15
0.88
0.87
0.95
0.98
2.02
0.97
1.89
0.96
3.10
3.21
f1 (ppm)

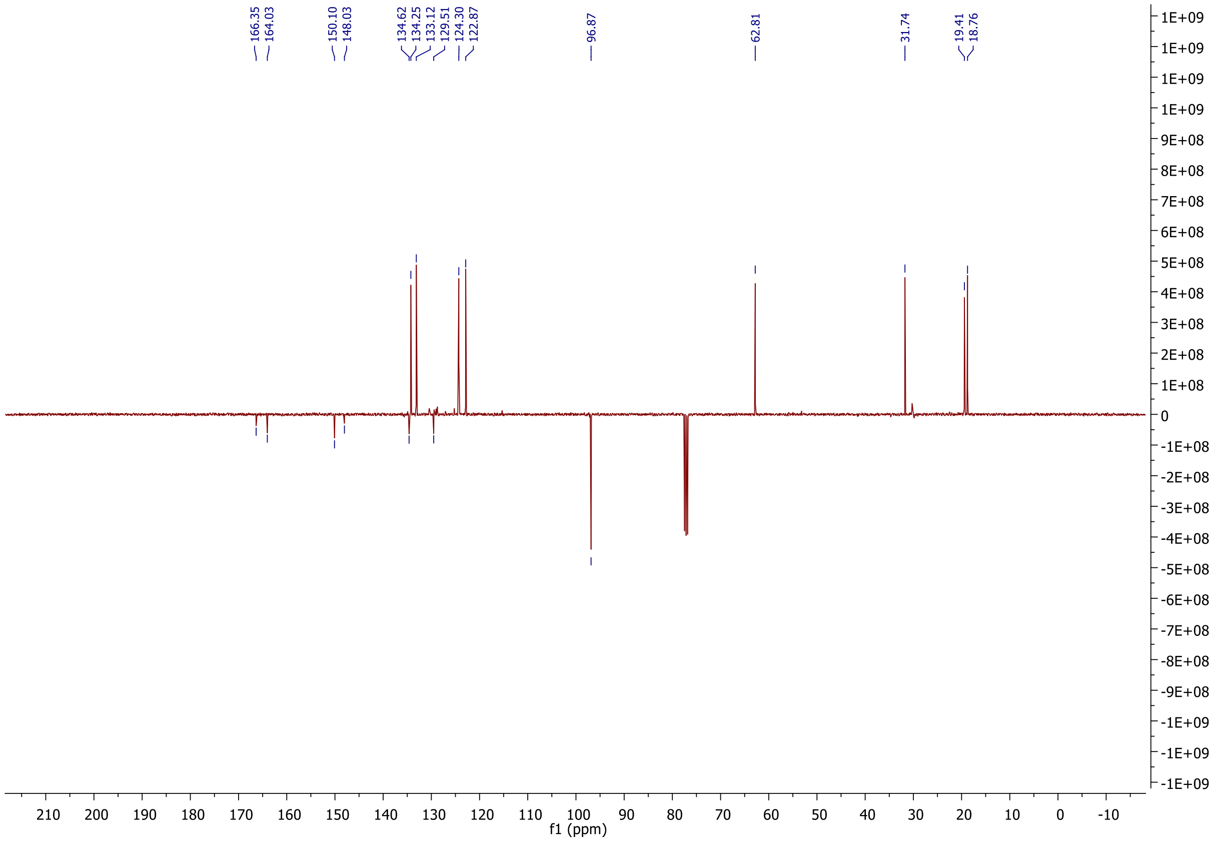
166.35
164.03
150.10
148.03
134.62
134.25
133.12
129.51
124.30
122.87
96.87
62.81
31.74
19.41
18.76
f1 (ppm)

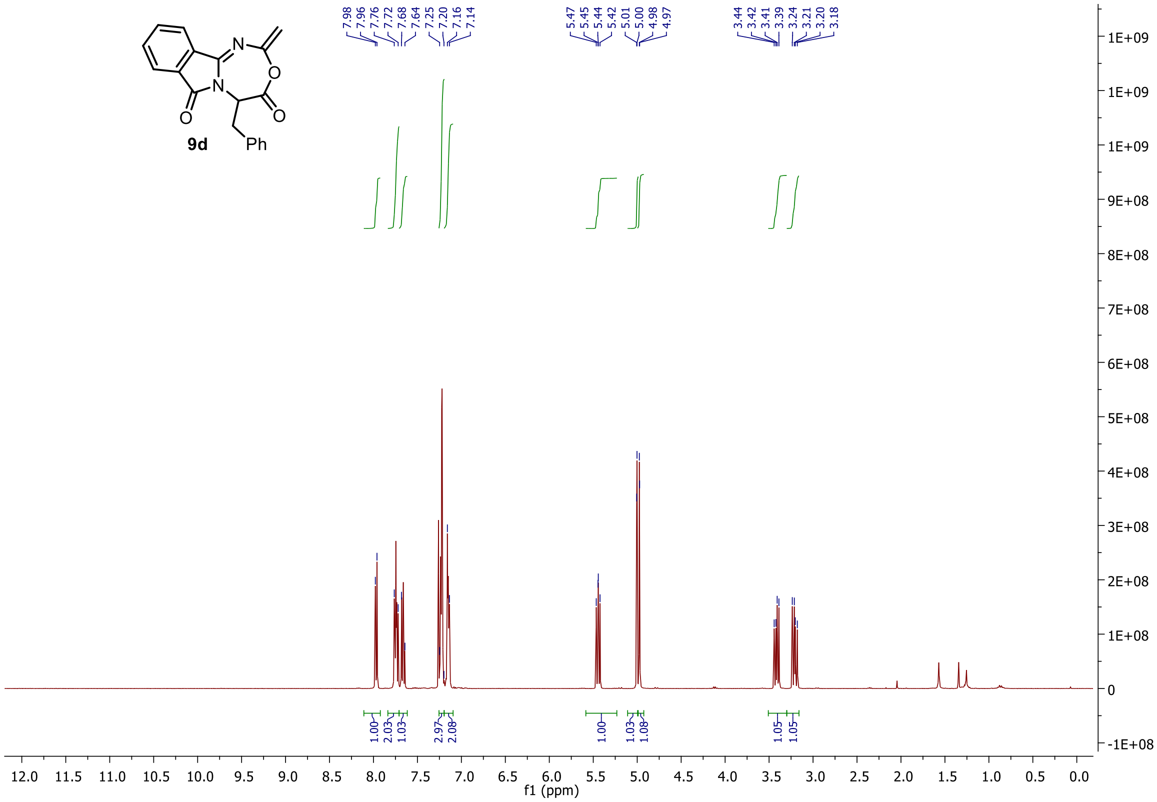

9d
Ph
N
O
7.98 7.96 7.76 7.72 7.68 7.64 7.25 7.20 7.16 7.14
5.47 5.45 5.44 5.42 5.01 5.00 4.98 4.97
3.44 3.42 3.41 3.39 3.24 3.21 3.20 3.18
1.00 2.03 1.03 2.97 2.08 1.00 1.03 1.08 1.05 1.05
f1 (ppm)

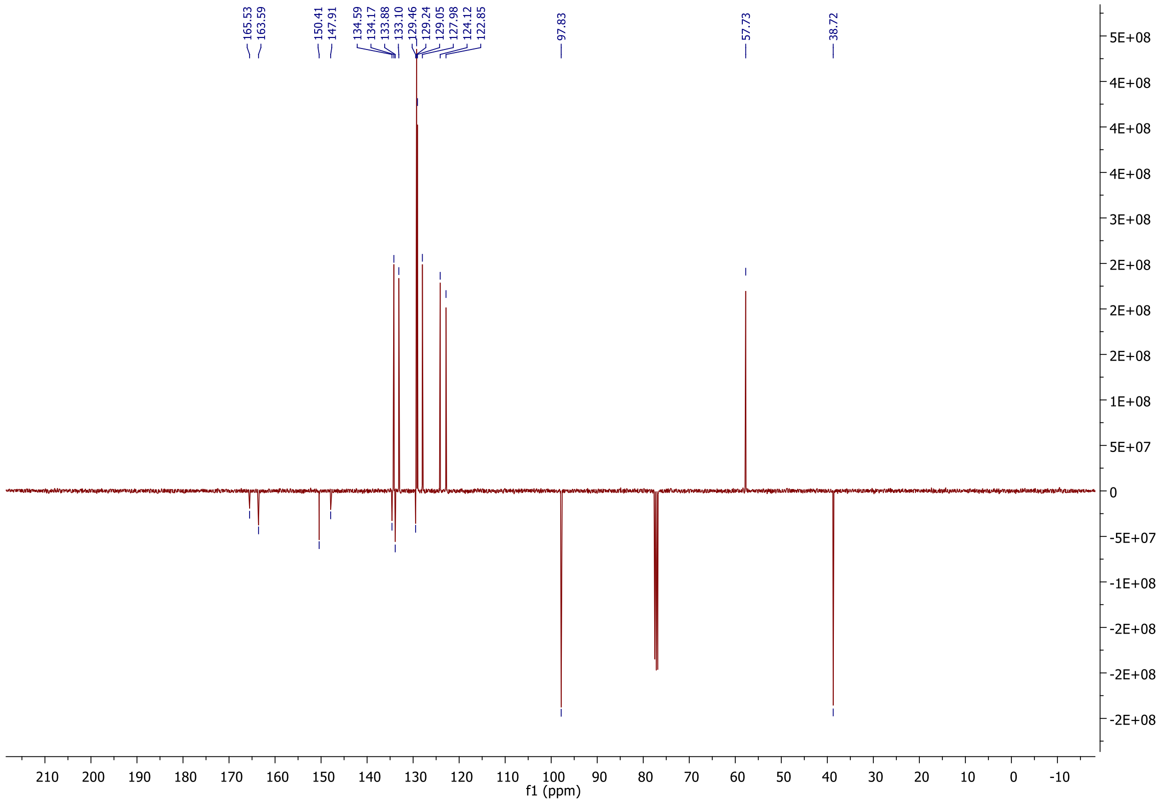

165.53 163.59 150.41 147.91 134.59 134.17 133.88 133.10 129.46 129.24 129.05 127.98 124.12 122.85 97.83 57.73 38.72
f1 (ppm)

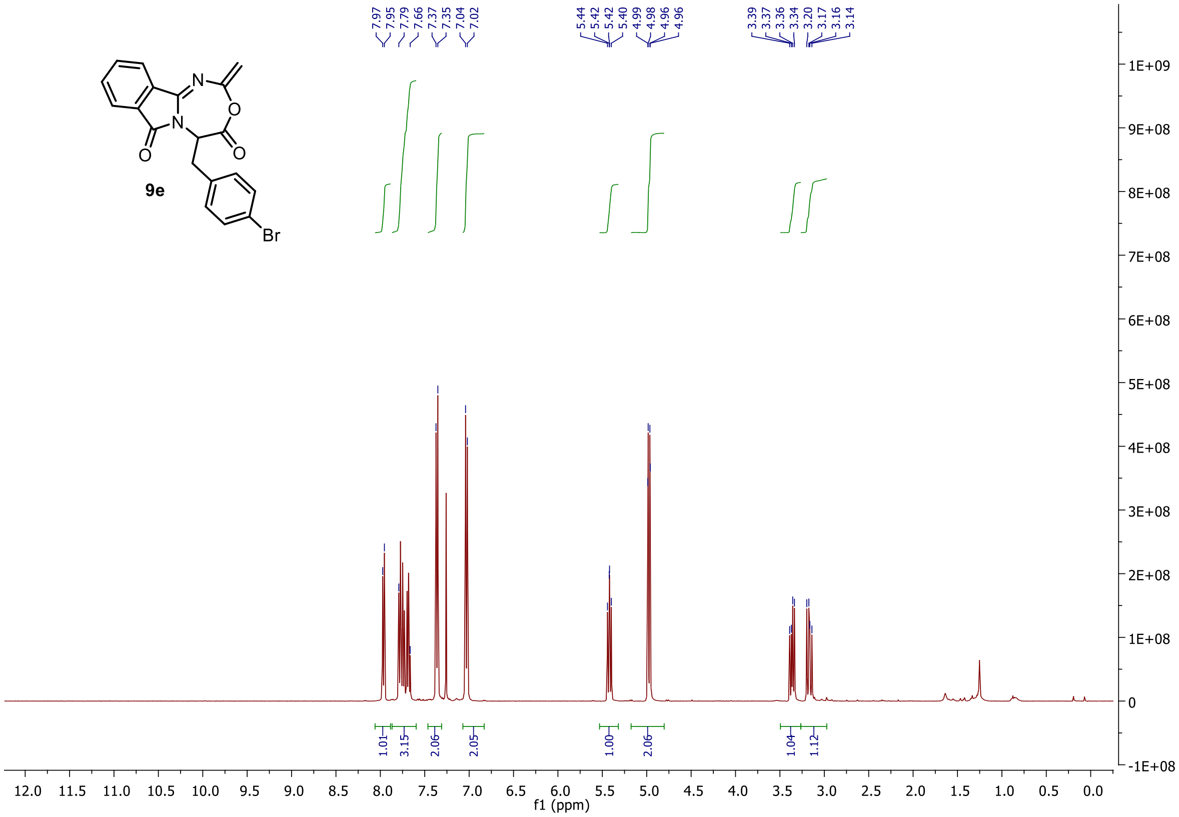
9e
Br
7.97
7.95
7.79
7.66
7.37
7.35
7.04
7.02
5.44
5.42
5.42
5.40
4.99
4.98
4.96
4.96
3.39
3.37
3.36
3.34
3.20
3.17
3.16
3.14
1.01
3.15
2.06
2.05
1.00
2.06
1.04
1.12
f1 (ppm)

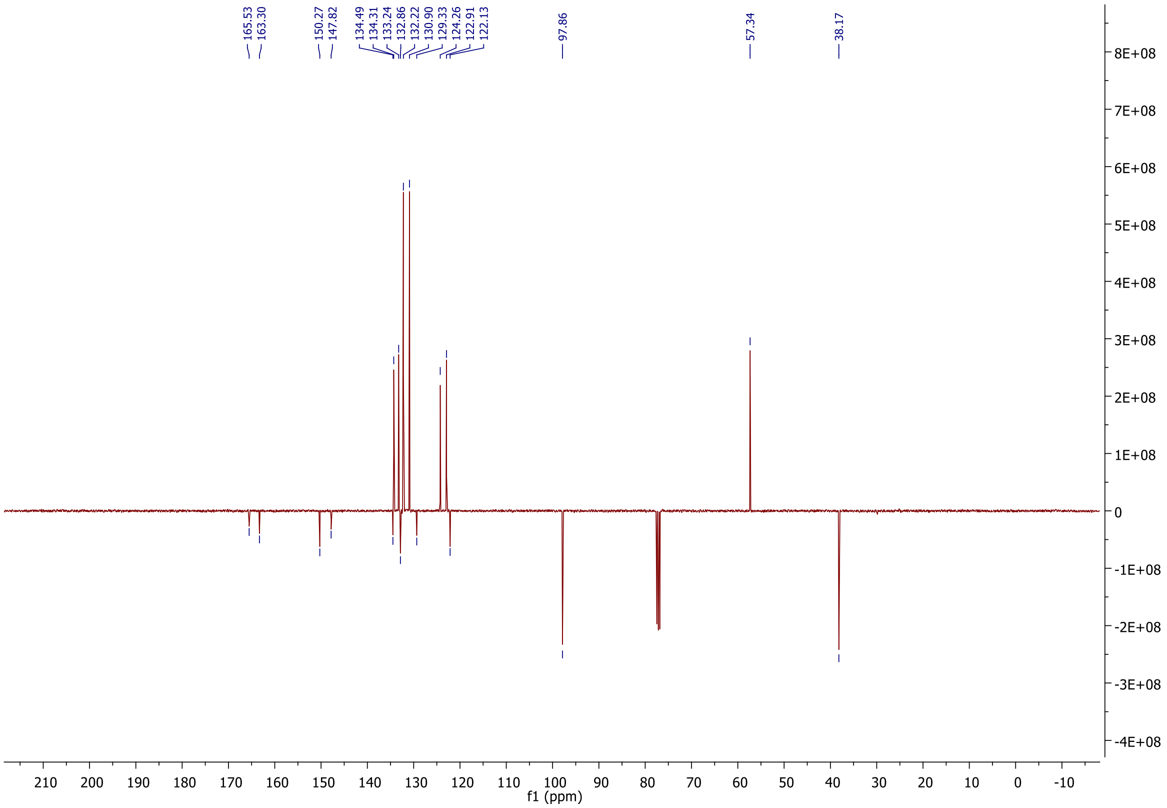
165.53
163.30
150.27
147.82
134.49
134.31
133.24
132.86
132.22
130.90
129.33
124.26
122.91
122.13
97.86
57.34
38.17
f1 (ppm)

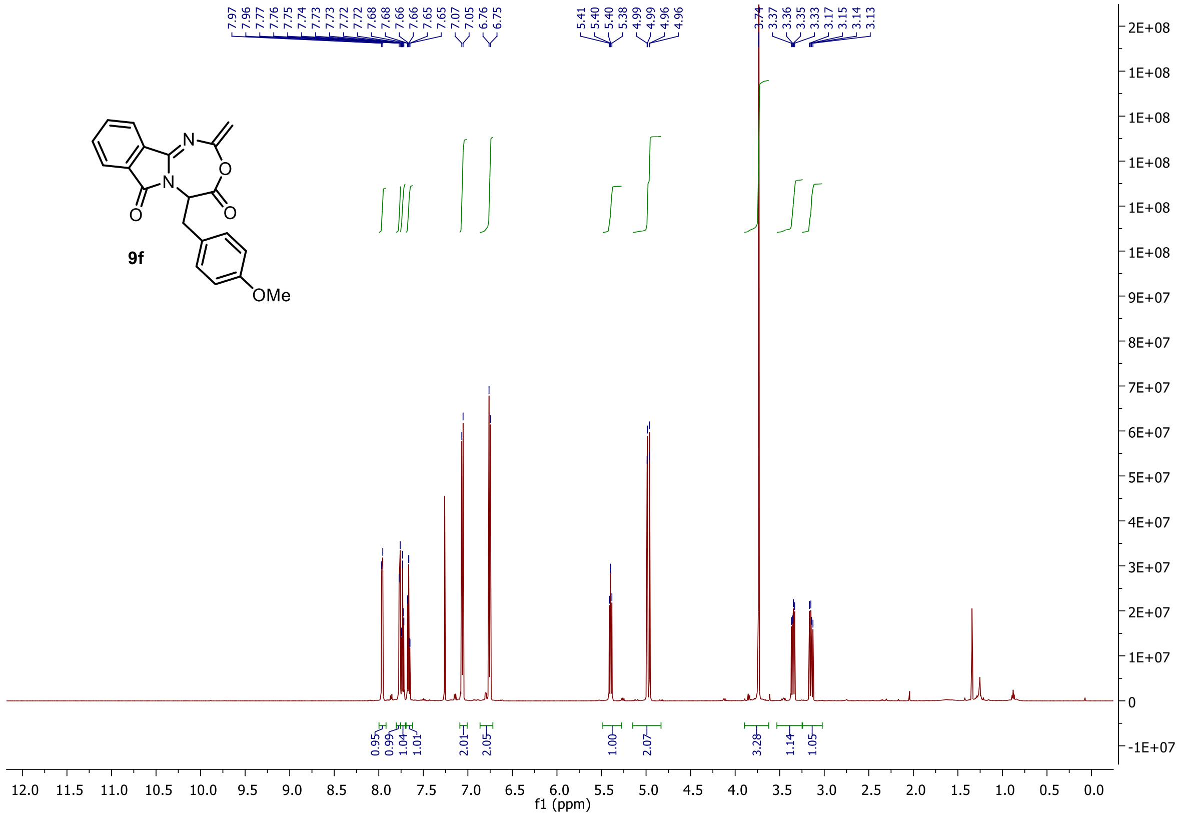
7.97 7.96 7.77 7.76 7.75 7.74 7.73 7.73 7.72 7.72 7.68 7.66 7.66 7.65 7.65 7.07 7.05 6.76 6.75
5.41 5.40 5.40 5.38 4.99 4.99 4.96 4.96
3.74 3.37 3.36 3.35 3.33 3.17 3.15 3.14 3.13
9f
OMe
0.95 0.99 1.04 1.01 2.01 2.05 1.00 2.07 3.28 1.14 1.05
f1 (ppm)

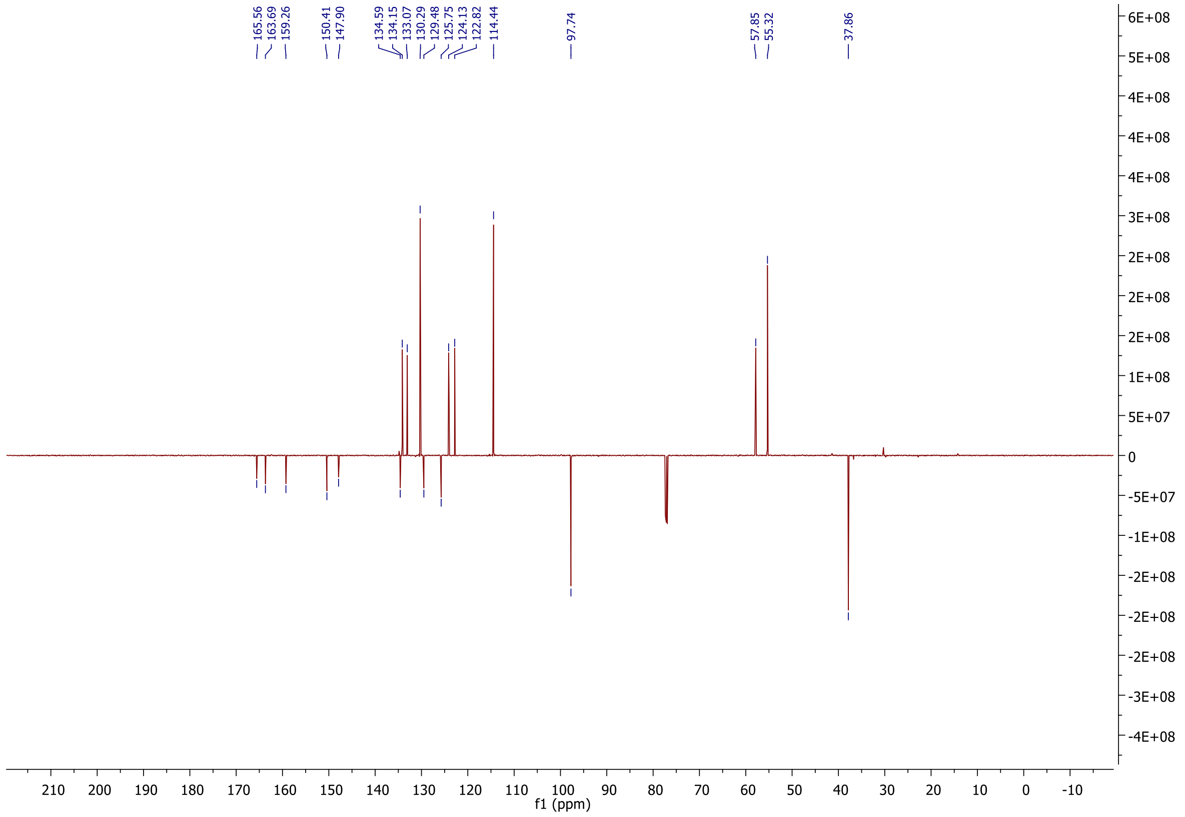
165.56 163.69 159.26 150.41 147.90 134.59 134.15 133.07 130.29 129.48 125.75 124.13 122.82 114.44 97.74 57.85 55.32 37.86
f1 (ppm)

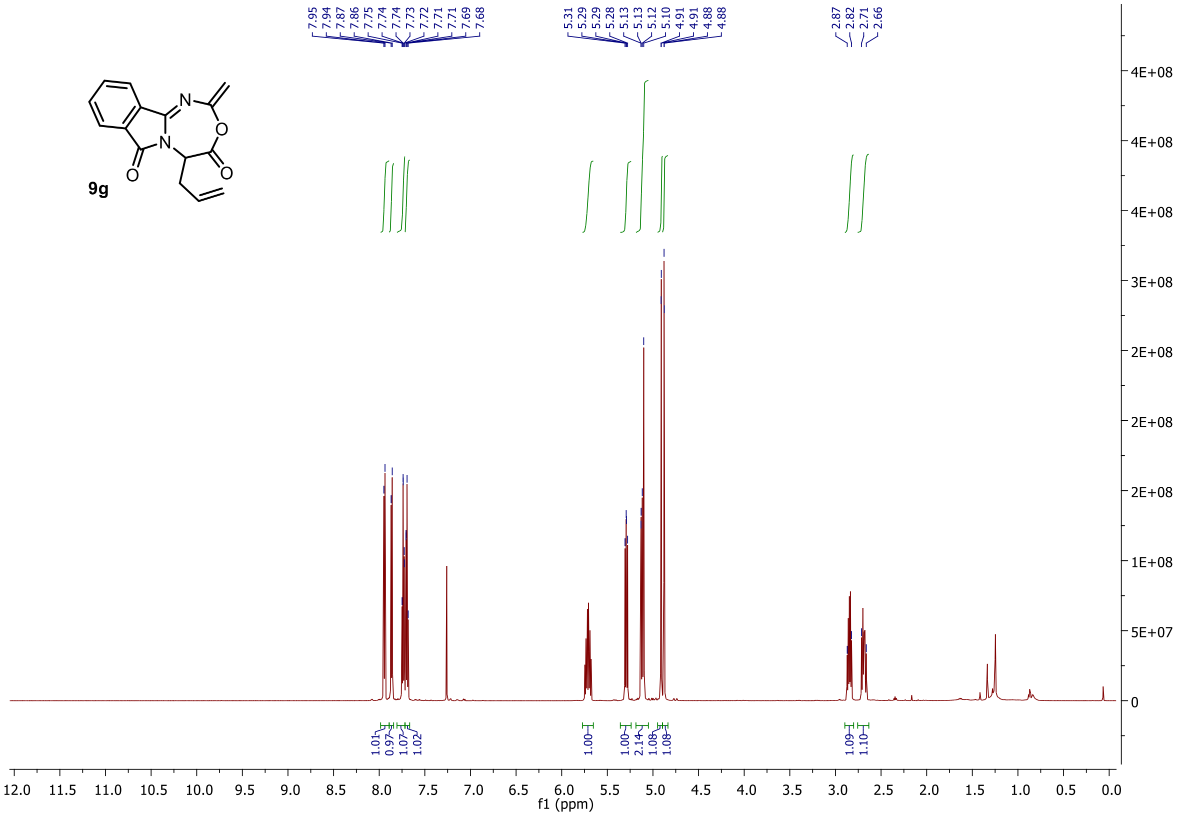
7.95 7.94 7.87 7.86 7.75 7.74 7.74 7.73 7.72 7.72 7.71 7.71 7.69 7.68
5.31 5.29 5.29 5.28 5.13 5.13 5.12 5.10 4.91 4.91 4.88 4.88
2.87 2.82 2.71 2.66
9g
1.01 0.97 1.07 1.02 1.00 1.00 2.14 1.08 1.08 1.09 1.10
f1 (ppm)

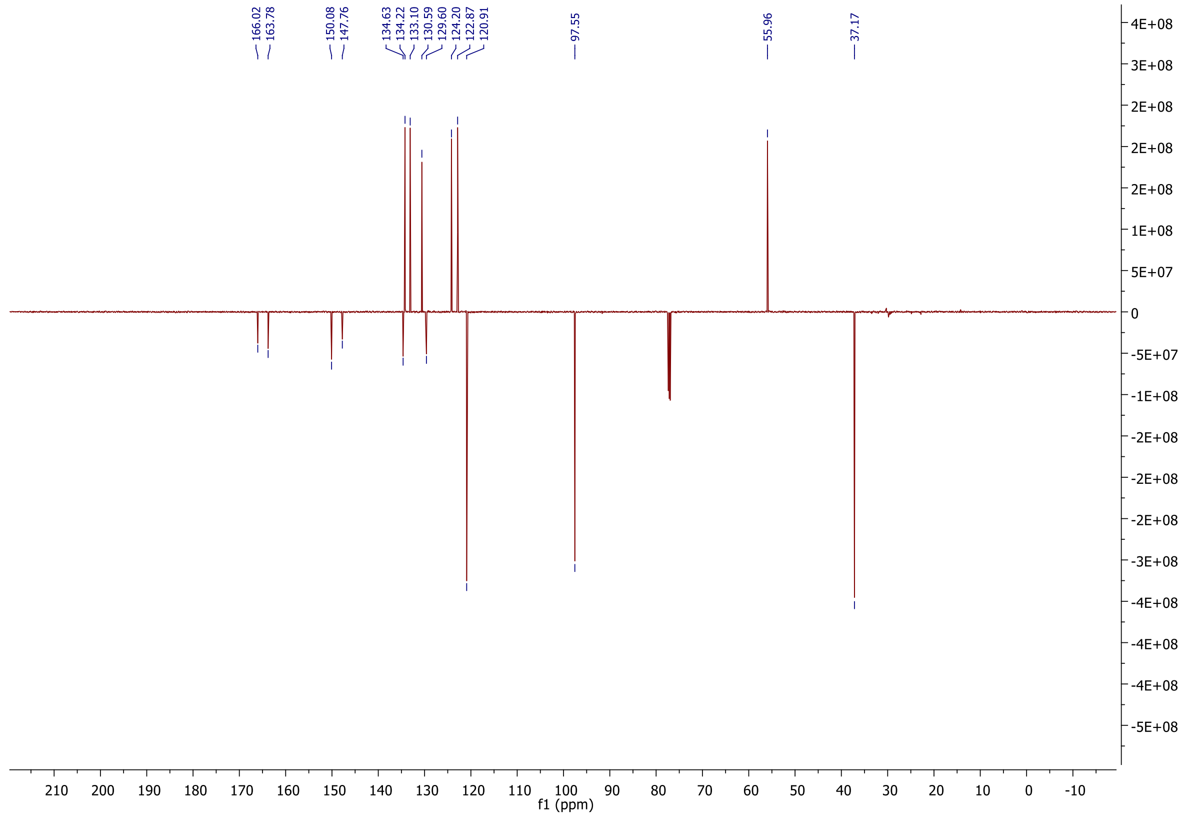
166.02 163.78 150.08 147.76 134.63 134.22 133.10 130.59 129.60 124.20 122.87 120.91 97.55 55.96 37.17
f1 (ppm)

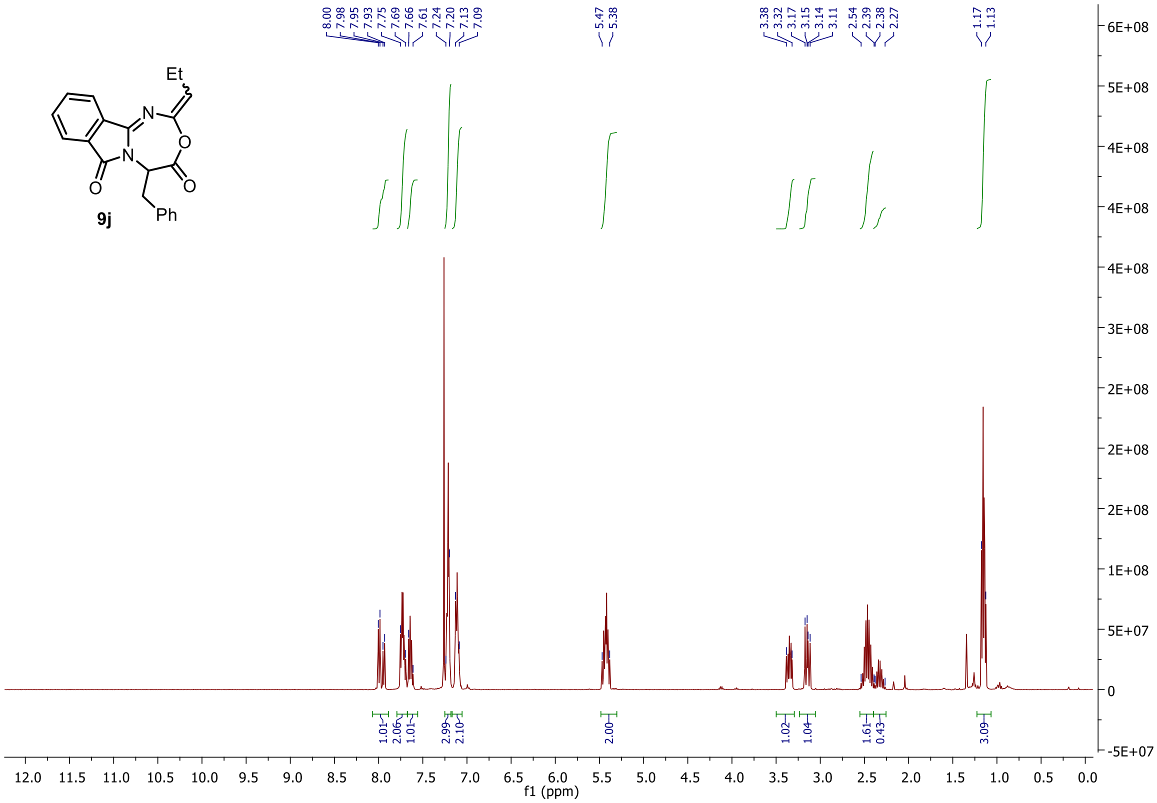
Et
N
O
N
O
O
Ph
9j
8.00
7.98
7.95
7.93
7.75
7.69
7.66
7.61
7.24
7.20
7.13
7.09
5.47
5.38
3.38
3.32
3.17
3.15
3.14
3.11
2.54
2.39
2.38
2.27
1.17
1.13
1.01
2.06
1.01
2.99
2.10
2.00
1.02
1.04
1.61
0.43
3.09
f1 (ppm)

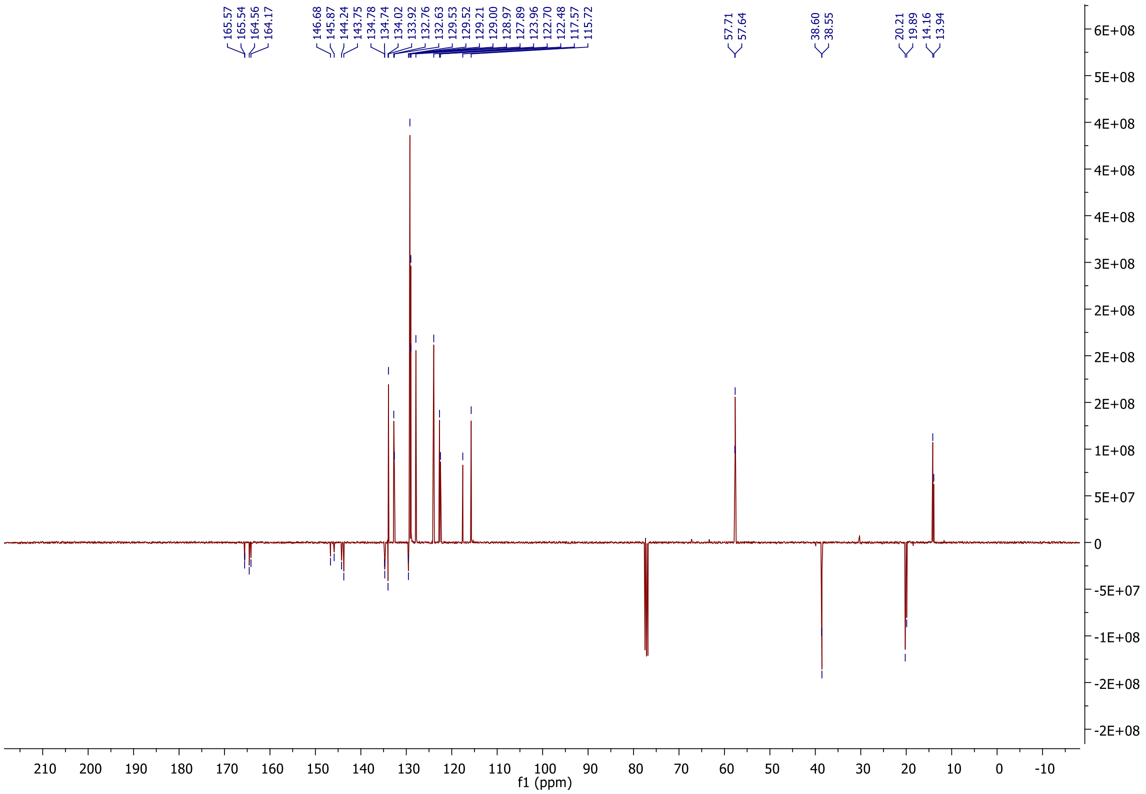
165.57
165.54
164.56
164.17
146.68
145.87
144.24
143.75
134.78
134.74
134.02
133.92
132.76
132.63
129.53
129.52
129.21
129.00
128.97
127.89
123.96
122.70
122.48
117.57
115.72
57.71
57.64
38.60
38.55
20.21
19.89
14.16
13.94
f1 (ppm)

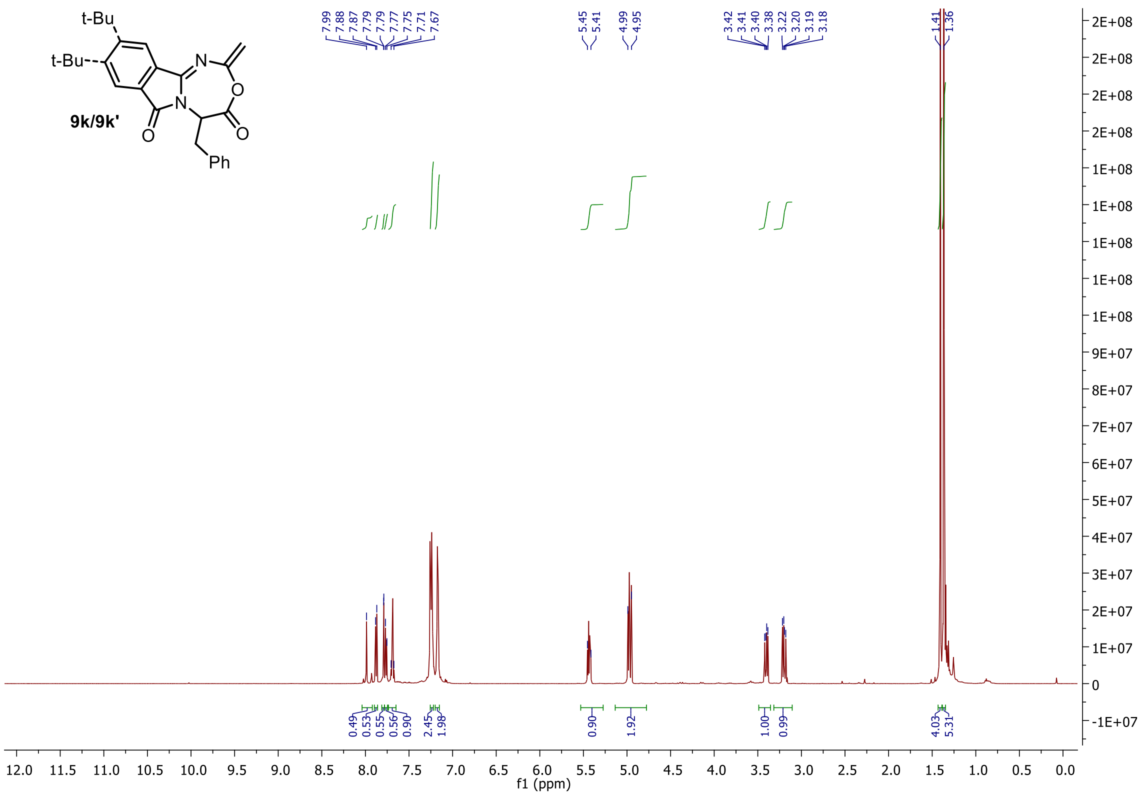
t-Bu
t-Bu
N
O
O
N
O
Ph
9k/9k'
7.99
7.88
7.87
7.79
7.79
7.77
7.75
7.71
7.67
5.45
5.41
4.99
4.95
3.42
3.41
3.40
3.38
3.22
3.20
3.19
3.18
1.41
1.36
0.49
0.53
0.55
0.56
0.90
2.45
1.98
0.90
1.92
1.00
0.99
4.03
5.31
f1 (ppm)

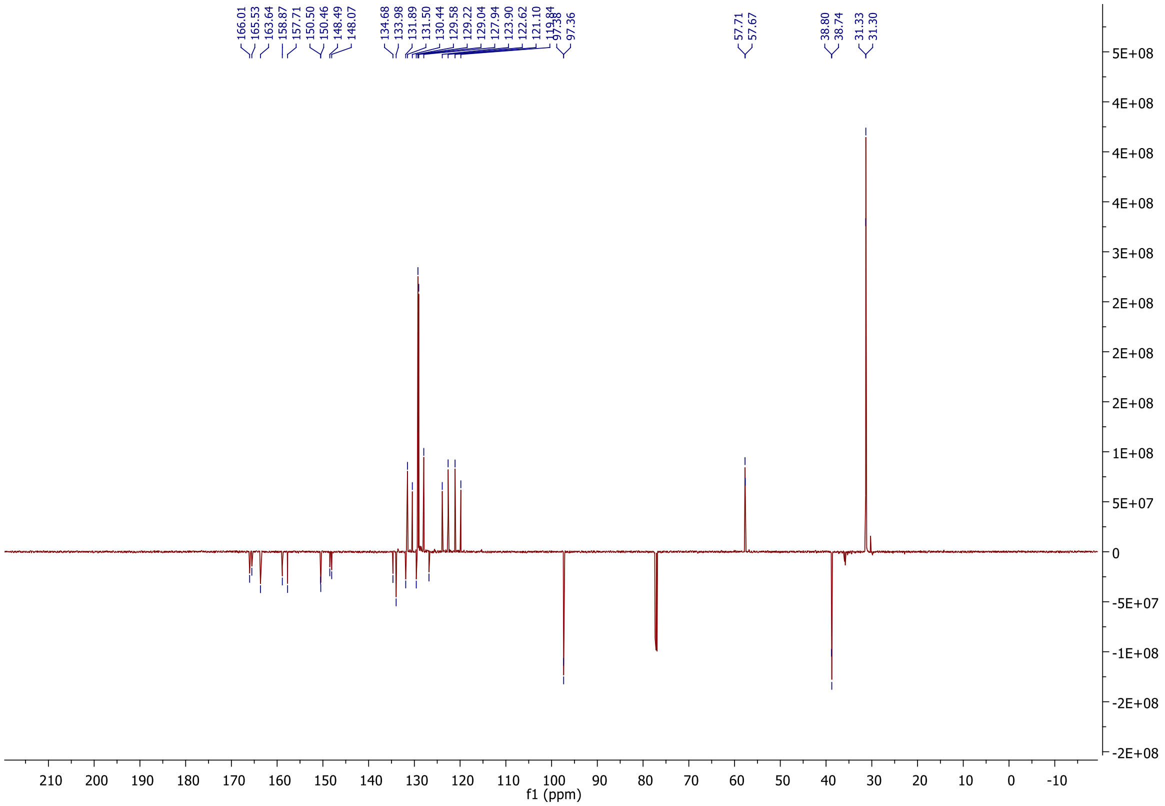
166.01
165.53
163.64
158.87
157.71
150.50
150.46
148.49
148.07
134.68
133.98
131.89
131.50
130.44
129.58
129.22
129.04
127.94
123.90
122.62
121.10
119.84
97.38
97.36
57.71
57.67
38.80
38.74
31.33
31.30
f1 (ppm)

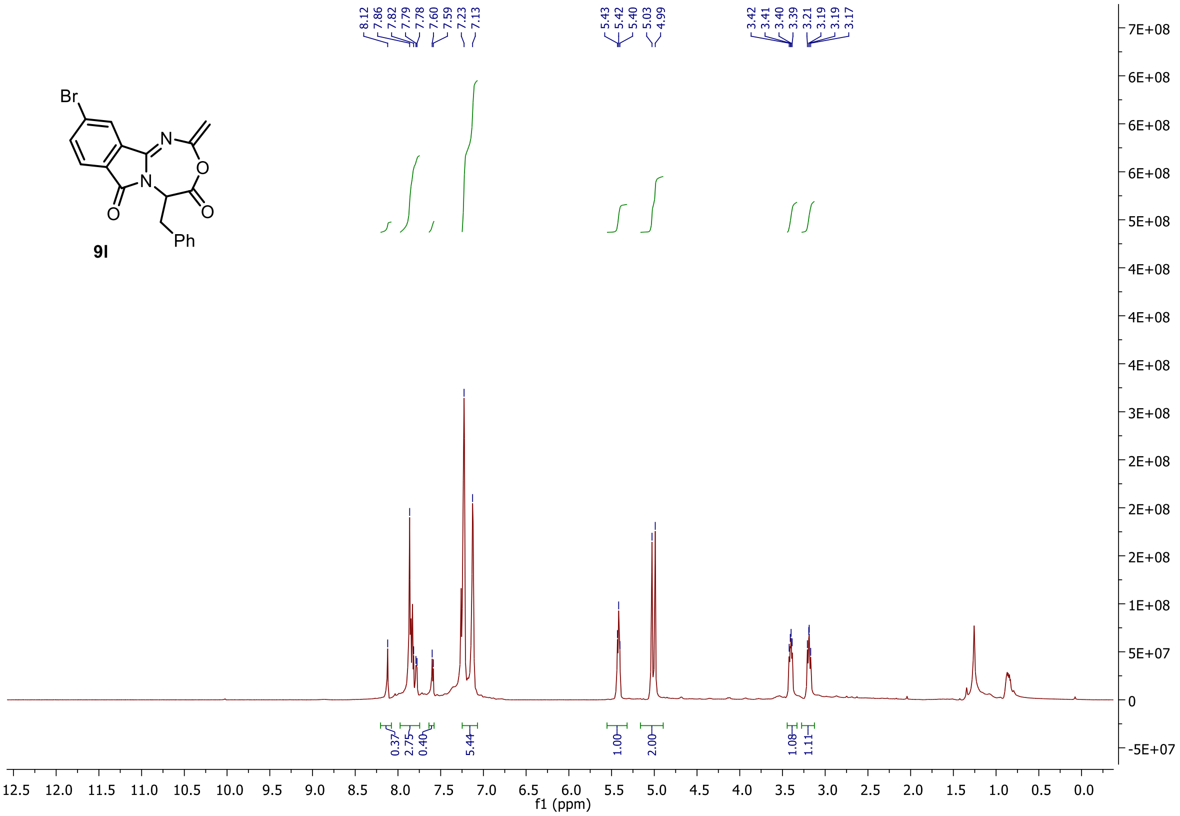
Br
N
O
N
O
O
Ph
9l
8.12
7.86
7.82
7.79
7.78
7.60
7.59
7.23
7.13
5.43
5.42
5.40
5.03
4.99
3.42
3.41
3.40
3.39
3.21
3.19
3.19
3.17
0.37
2.75
0.40
5.44
1.00
2.00
1.08
1.11
f1 (ppm)

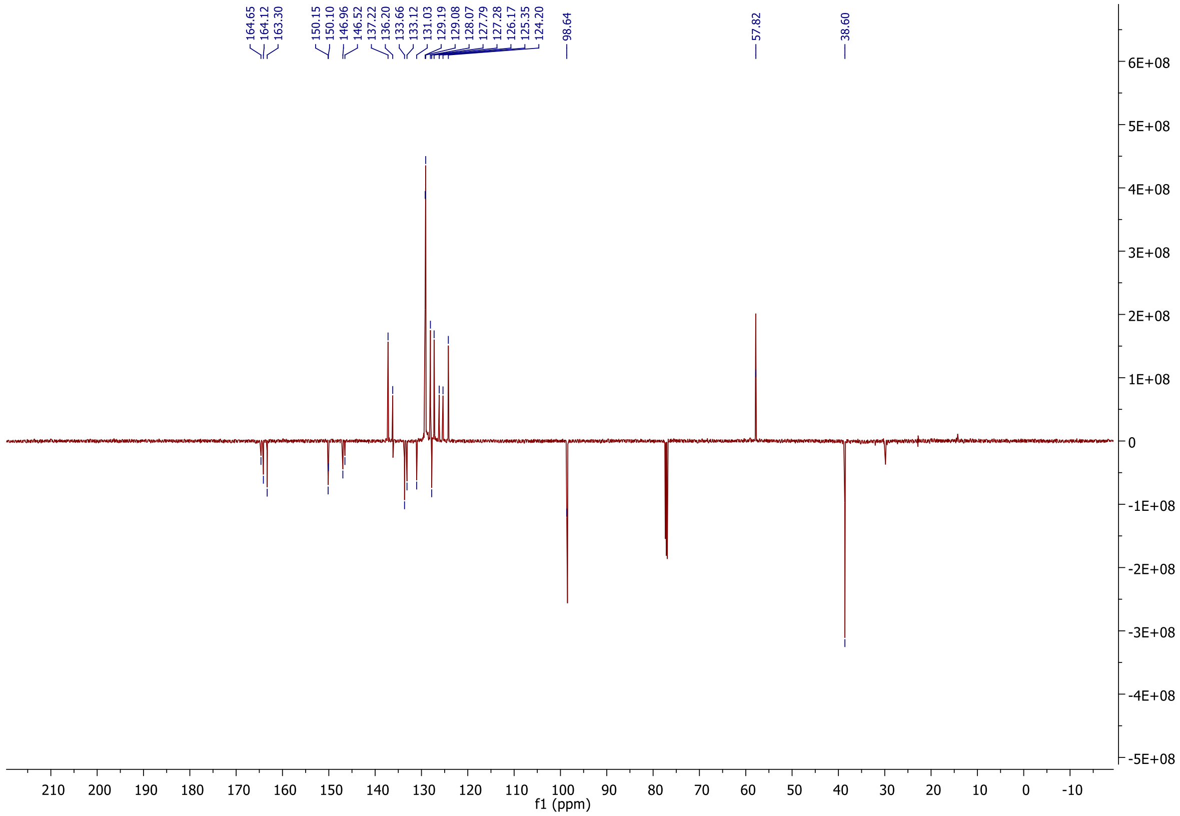
164.65
164.12
163.30
150.15
150.10
146.96
146.52
137.22
136.20
133.66
133.12
131.03
129.19
129.08
128.07
127.79
127.28
126.17
125.35
124.20
98.64
57.82
38.60
f1 (ppm)

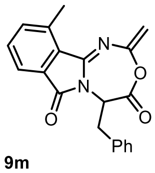

9m

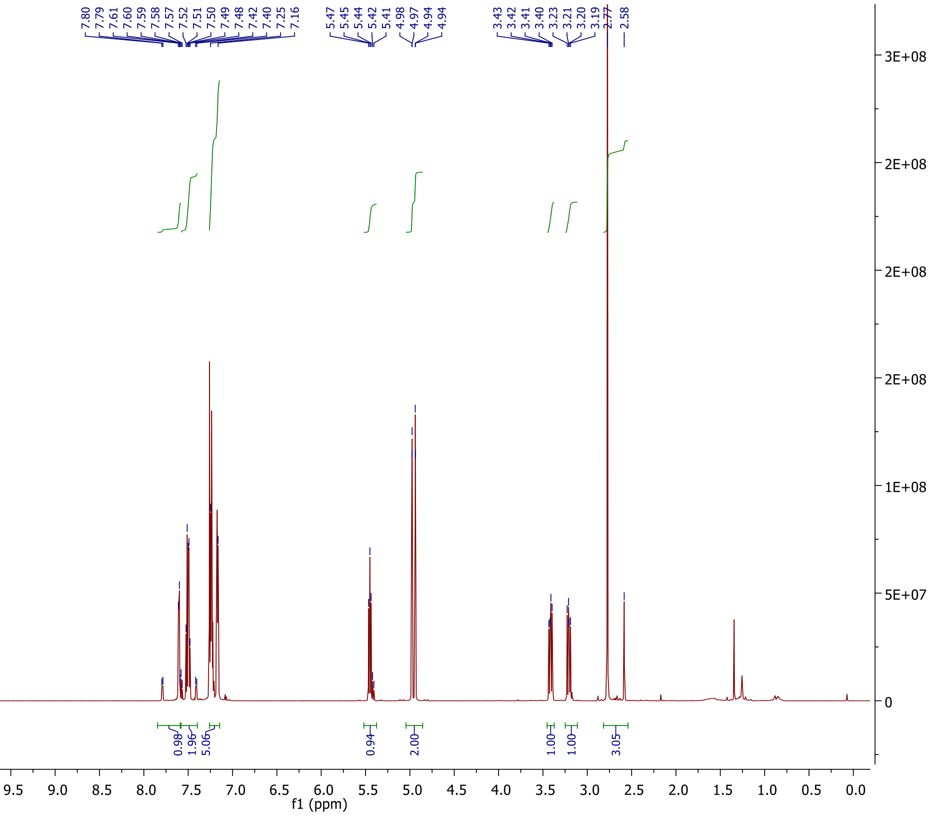
7.80
7.79
7.61
7.60
7.59
7.58
7.57
7.52
7.51
7.50
7.49
7.48
7.42
7.40
7.25
7.16
5.47
5.45
5.44
5.42
5.41
4.98
4.97
4.94
4.94
3.43
3.42
3.41
3.40
3.23
3.21
3.20
3.19
2.77
2.58
0.98
1.96
5.06
0.94
2.00
1.00
1.00
3.05
f1 (ppm)

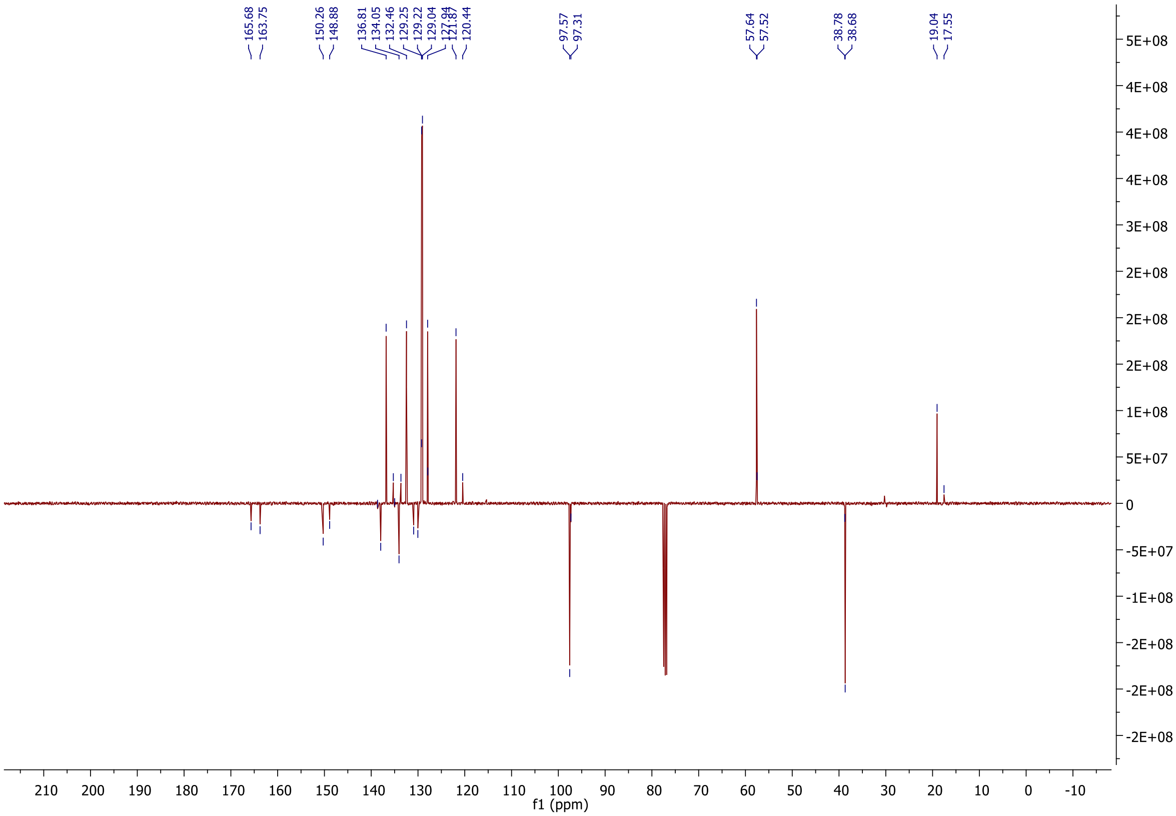
165.68
163.75
150.26
148.88
136.81
134.05
132.46
129.25
129.22
129.04
127.94
121.87
120.44
97.57
97.31
57.64
57.52
38.78
38.68
19.04
17.55
f1 (ppm)

VI. X-ray Analysis

The X-ray intensity data were measured on Bruker D8 Venture diffractometer equipped with multilayer monochromator, Mo K/α INCOATEC micro focus sealed tube and Oxford cooling system. The structure was solved by *direct methods* and refined by *full-matrix least-squares techniques*. Non-hydrogen atoms were refined with *anisotropic displacement parameters*. Hydrogen atoms were inserted at calculated positions and refined with riding model. The following software was used: *Bruker SAINT software package*[i] using a narrow-frame algorithm for frame integration, *SADABS*[ii] for absorption correction, *OLEX2*[iii] for structure solution, refinement, molecular diagrams and graphical user-interface, *Shelxle*[iv] for refinement and graphical user-interface *SHELXS-2015*[v] for structure solution, *SHELXL-2015*[vi] for refinement, *Platon*[vii] for symmetry check. Experimental data and CCDC-Codes Experimental data and CCDC-Code (Available online: http://www.ccdc.cam.ac.uk/conts/retrieving.html) can be found in Table 1. Crystal data, data collection parameters, and structure refinement details are given in Tables 2 & 3. Asymmetric unit visualized in Figure 1.

Table 1 Experimental parameter and CCDC-Code.

Sample	Machine	Source	Temp.	Detector Distance	Time/ Frame	#Frames	Frame width	CCDC
	Bruker		[K]	[mm]	[s]		[°]	
9b	D8	Mo	100	34	40	2171	0.500	1987479

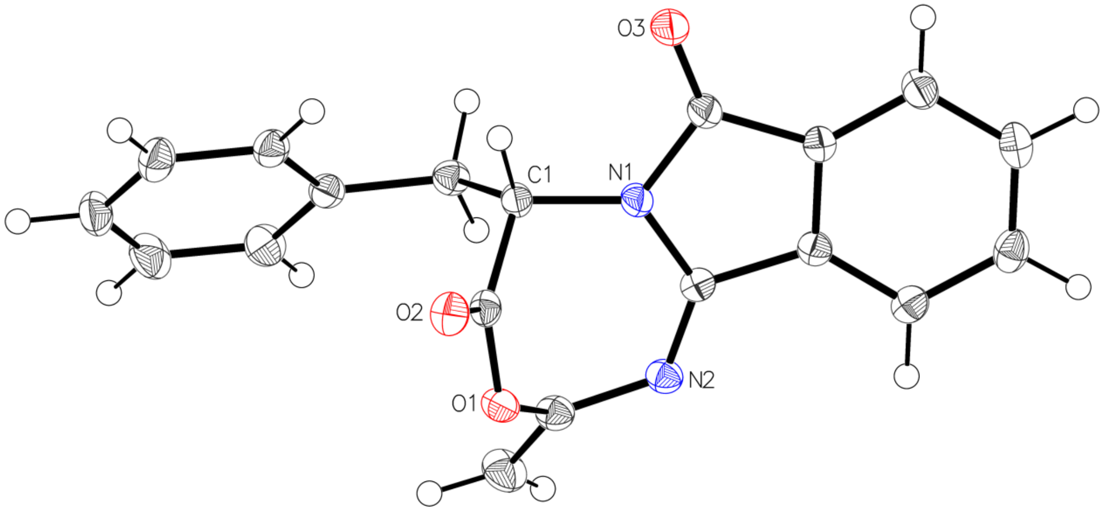

Figure 1 Asymmetric unit, drawn with 50% displacement ellipsoid. The bond precision for C-C single bonds is 0.0024Å. The chiral C_1 is defined in "S" by the starting material.

Table 2 Sample and crystal data.

**Chemical formula**	C19H14N2O3	**Crystal system**	orthorhombic	
**Formula weight [g/mol]**	318.32	**Space group**	*P21212*	
**Temperature [K]**	100	**Z**	4	
**Measurement method**	\f and \w scans	**Volume [Å^3]**	1512.6(2)	
**Radiation (Wavelength [Å])**	MoKα (λ = 0.71073)	**Unit cell dimensions [Å] and [°]**	6.7669(5)	90
**Crystal size / [mm^3]**	0.2 × 0.08 × 0.06		13.7940(11)	90
**Crystal habit**	clear colourless block		16.2050(13)	90
**Density (calculated) / [g/cm^3]**	1.398	**Absorption coefficient / [mm^{-1}]**	0.096	
**Abs. correction Tmin**	0.6955	**Abs. correction Tmax**	0.746	
**Abs. correction type**	multiscan	**F(000) [e$^-$]**	664	

Table 3 Data collection and structure refinement.

**Index ranges**	-9 ≤ h ≤ 9, -19 ≤ k ≤ 19, -22 ≤ l ≤ 22	**Theta range for data collection [°]**	5.028 to 60.146	
**Reflections number**	75023	**Data / restraints / parameters**	4463/0/217	
**Refinement method**	Least squares	**Final R indices**	all data	R1 = 0.0429, wR2 = 0.0888
**Function minimized**	Σ w(F_o^2 - F_c^2)^2		I>2σ(I)	R1 = 0.0359, wR2 = 0.0839
**Goodness-of-fit on F^2**	1.028	**Weighting scheme**	w=1/[σ^2(Fo^2)+(0.0403P)^2 +0.4070P]	
**Largest diff. peak and hole [e Å^{-3}]**	0.25/-0.21		where P=(F_o^2+2F_c^2)/3	

[i] Bruker SAINT v8.38B Copyright © 2005-2019 Bruker AXS
[ii] L. Krause, R. Herbst-Irmer, G. M. Sheldrick and D. Stalke. J. Appl. Cryst. (2015). 48, 3-10.
[iii] Dolomanov, O.V., Bourhis, L.J., Gildea, R.J, Howard, J.A.K. & Puschmann, H. , OLEX2, (2009), J. Appl. Cryst. 42, 339-341
[iv] C. B. Huebschle, G. M. Sheldrick and B. Dittrich, ShelXle: a Qt graphical user interface for SHELXL, J. Appl. Cryst., 44, (2011) 1281-1284
[v] Sheldrick, G. M.. Acta Cryst (2008). A64, 112-122
[vi] Sheldrick, G. M.. Acta Cryst. (2015). C71, 3-8
[vii] A. L. Spek, Acta Cryst. 2009, D65, 148-155.
